# Comparative genomics of monotremes provides insights into the early evolution of mammalian epidermal differentiation genes

**DOI:** 10.1038/s41598-024-51926-7

**Published:** 2024-01-16

**Authors:** Julia Steinbinder, Attila Placido Sachslehner, Karin Brigit Holthaus, Leopold Eckhart

**Affiliations:** Department of Dermatology, Medical University of Vienna, Vienna, Austria

**Keywords:** Molecular evolution, Evolutionary biology, Differentiation

## Abstract

The function of the skin as a barrier against the environment depends on the differentiation of epidermal keratinocytes into highly resilient corneocytes that form the outermost skin layer. Many genes encoding structural components of corneocytes are clustered in the epidermal differentiation complex (EDC), which has been described in placental and marsupial mammals as well as non-mammalian tetrapods. Here, we analyzed the genomes of the platypus (*Ornithorhynchus anatinus*) and the echidna (*Tachyglossus aculeatus*) to determine the gene composition of the EDC in the basal clade of mammals, the monotremes. We report that mammal-specific subfamilies of EDC genes encoding small proline-rich proteins (SPRRs) and late cornified envelope proteins as well as single-copy EDC genes such as involucrin are conserved in monotremes, suggesting that they have originated in stem mammals. Monotremes have at least one gene homologous to the group of filaggrin (*FLG*), *FLG2* and hornerin (*HRNR*) in placental mammals, but no clear one-to-one pairwise ortholog of either *FLG*, *FLG2* or *HRNR*. Caspase-14, a keratinocyte differentiation-associated protease implicated in the processing of filaggrin, is encoded by at least 3 gene copies in the echidna. Our results reveal evolutionarily conserved and clade-specific features of the genetic regulation of epidermal differentiation in monotremes.

## Introduction

Mammals have evolved a skin that is unique among terrestrial vertebrates^1^. The superficial epithelial layer of the skin, the epidermis is soft and pliable, yet resistant to mechanical stress and protective against excessive water loss in most mammals. Characteristically, epithelial-stromal interactions lead to the development of hair follicles on most parts of the typical mammalian body surface, generating a protective coat of hair, made of cornified dead epithelial cells. This organisation of the skin is similar to the skin of birds, in which feathers cover a soft interfollicular epidermis. Mammalian skin contains various glands, among which sebaceous glands are generally most numerous due their association with hair follicles^2^.

Like in other amniotes, the mammalian epidermis and the epithelial components of skin appendages are formed by keratinocytes. As many skin functions are exerted by cornified structures and the epidermis is continuously regenerated, keratinocytes are genetically programmed to undergo cell division in the basal layer of the epidermis and differentiation towards cornification in the suprabasal layers of the epidermis^3^. The critical steps of cornification occur in the outermost living layers of the epidermis where a large array of proteins are covalently cross-linked by transglutaminases to form the so-called cornified envelope adjacent to the cell membrane of keratinocytes which thereby are converted to corneocytes, the cell remnants building the stratum corneum^4^. Research over the past 45 years has revealed that many protein components of the cornified envelope, proteins interacting with keratin intermediate filaments and antimicrobial proteins are encoded by a cluster of genes, called the epidermal differentiation complex (EDC)^5–7^. The genes of the EDC were first reported for humans^8,9^ and the main biomedical model species, the mouse, and later, in partial or complete form, also for the sheep^10^, whales^11,12^, and other mammals^13^. Comparative genomics and transcriptomics revealed that equivalents of the mammalian EDC also exist in reptiles, birds and amphibians^13–21^.

The EDC consists of four types of genes which are characterized by the organisation of their exons and by specific features, such as presence or absence of folded domains and highly biased amino acid compositions, of the proteins they encode^6,22^. S100A genes encode proteins folding into an S100 domain. Most of them have one non-coding and two protein-coding exons and their expression is not strictly confined to keratinocytes^23,24^. A closely related EDC gene type encodes S100 fused-type proteins (SFTPs), which consist of an amino-terminal S100 domain and a presumably intrinsically disordered carboxy-terminal domain of up to 4000 amino acid residues^6,7,25^. Like S100A genes, SFTP genes have one non-coding and two protein-coding exons. By contrast, single-coding-exon EDC (SEDC) genes have only one exon with protein-coding sequence and one 5′-terminal non-coding exon. SEDC proteins such as loricrin are likely intrinsically disordered^26^, although some secondary structure elements are possible^27^. The fourth type of EDC genes are peptidoglycan recognition protein (PGLYRP) genes, which have more than 3 protein coding exons. PGLYRP3 and 4 are structurally clearly distinct from other EDC proteins^22^. Relatively common mutations of the SEDC genes, *late cornified envelope (LCE) 3B* and *LCE3C*, and of the SFTP gene, *Filaggrin* (*FLG*), are associated with elevated risk of psoriasis and atopic dermatitis, respectively^28,29^. Polymorphisms of other EDC genes are implicated in the genetics of human skin and hair traits^30–32^. Despite conservation of the basic types of EDC genes and conservation of a few 1:1 orthologs such as cornulin^33^, the EDCs of mammals and sauropsids (reptiles and birds) differ substantially with regard to their gene content. One-to-one orthology means that a gene in the last common ancestor gave rise to only one gene in the first taxon and only one gene in the other taxon. Duplications of genes after speciation lead to more complex orthology relationships^34^. Important examples of differences in the EDCs of mammals and sauropsids are the genes encoding corneous beta-proteins, also known as beta-keratins, which are critical for scales and feathers in sauropsids and absent in mammals^18^, and LCE genes, which exist in mammals but not in sauropsids^22,35^. However, the comparison between the two main clades of amniotes has been incomplete so far, because the EDC has not been analyzed in the basal clade of mammals, the monotremes.

The platypus (*Ornithorhynchus anatinus*) and four species of echidnas, i.e. the short-beaked echidna (*Tachyglossus aculeatus*) and long-beaked echidnas (*Zaglossus bruijni*, *Z. attenboroughi*, *Z. bartoni*), are grouped in the taxon Monotremata, also referred to as protherians. Monotremes diverged from therians (marsupials and eutherians) 180–190 million years ago^35^. They have several features that distinguish them from other mammals. Most notably they lay eggs, but they also develop two types of special sensory skin structures that largely consist of keratinocytes interacting with nerves: so-called push rods, which act as mechanoreceptors, and sensory mucous glands, which provide an electric sense^36,37^. There are also phenotypic differences between the platypus and the echidnas, which diverged approximately 55 million years ago^35^. The platypus is semi-aquatic and catches its prey in rivers. It has a very dense coat of hair and a duck bill-like snout. Echidnas feed on ants and termites, and they develop spines^38^. The genomes of the platypus (*O. anatinus*) and the short-beaked echidna (*T. aculeatus*), hereafter referred to as echidna, have been sequenced^36,39^. Keratin intermediate filament proteins implicated in epidermal differentiation were reported to be conserved in the platypus^40^.

Here, we analyzed the genomes of the platypus and the echidna and annotated EDC genes that had not been predicted previously. The data on the EDCs of monotremes, as the phylogenetically basal mammals, together with data on other mammalian EDCs and non-mammalian EDCs, enable us to map the evolutionary origins of EDC genes with orthologs in humans.

## Results

### Identification of the epidermal differentiation complex (EDC) in monotremes

**Figure 1:**
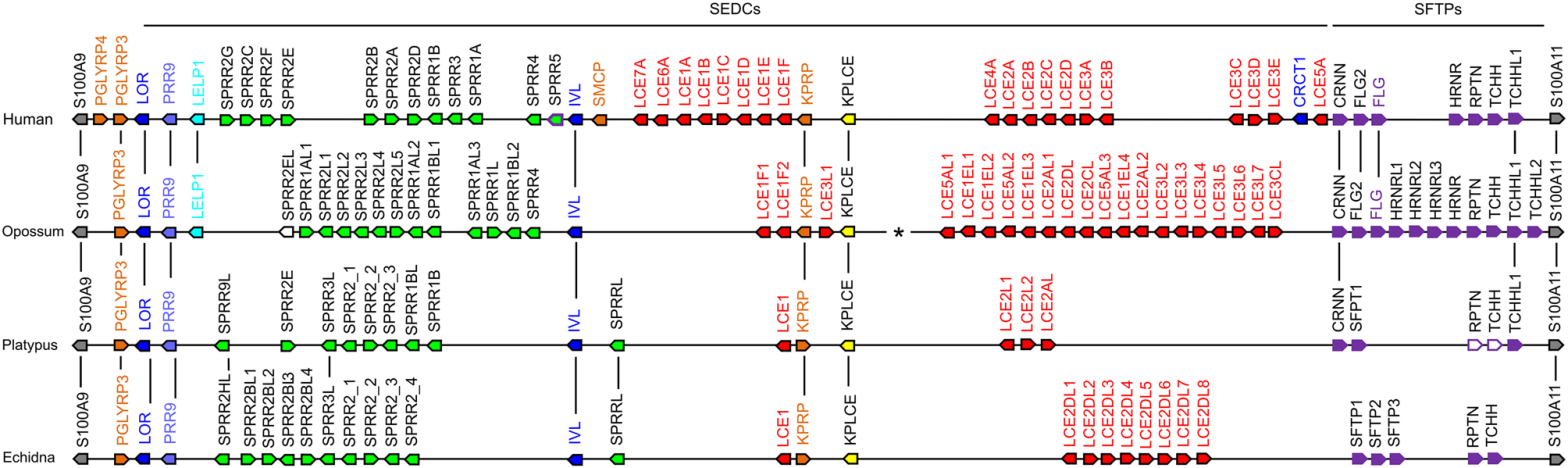
Comparison of EDC genes in monotremes, marsupial and placental mammals. The genes between *S100A9* and *S100A11*, representing the core region of the EDC, are illustrated as arrows pointing in the direction of transcription. Gene orthologies are marked by vertical lines, and gene families are depicted as identically colored arrows. White arrows indicate genes with a disrupted coding sequence by either premature stop codons or frameshifts. The opossum EDC locus is split and non-EDC genes are located at the position indicated by an asterisk. Species: Human (*Homo sapiens*), opossum *(Monodelphis domestica*), platypus (*Ornithorhynchus anatinus*) and echidna (*Tachyglossus aculeatus*). SEDC, single-coding-exon EDC gene; SFTP, S100 fused-type protein.

To define the organisation of the EDC in monotremes, we focused our investigation on the core region of the EDC which is flanked by orthologs of *S100A9* and *S100A11* in the genomes of humans and other amniotes reported so far^6–18^ (Fig. 1). S100A genes located outside of this core region in the peripheral segments of the EDC were not investigated here. We identifed SEDC and SFTP genes of the platypus and the echidna (*T. aculeatus*) using a published approach that depends on iterative BLAST searches and comparison of conserved splice sites^15,22^. The resulting lists of EDC genes included multiple genes that were not annotated prior to this study (Supplementary Tables S1, S2; Supplementary Figs. S1, S2). To facilitate the comparison of the EDC in monotremes and marsupials, we also identified the EDC genes of the opossum (*Monodelphis domestica*) (Supplementary Table S3; Supplementary Fig. S3). The arrangement of EDC genes was compared between echidna, platypus, opossum and human (Fig. 1).

The overall organisation of the EDC with regard to the arrangement of the main gene types is conserved in monotremes and therian mammals. Located next to *S100A9*, *PGLYRP3* is the only gene that does not belong to the SEDC or SFTP classes within the core region of the monotreme EDC. Humans and cattle have an additional member of the PGLYRP gene family, *PGLYRP4*, in the EDC^12^, suggesting that *PGLYRP4* has originated by tandem duplication of *PGLYRP3* in placentals. Eighteen and twenty-five SEDC genes were identified in platypus and echidna, respectively, which are numbers comparable to those in human (Fig. 1). However, we cannot exclude that some genes were not identified in our search. Finally, 3 and 5 apparently protein-coding SFTP genes were identified in platypus and echidna, respectively (Fig. 1, for more detailed information see the section “The content of SFTP genes differs between monotremes and other mammals”). The platypus has 2 further SFTP genes, the nucleotide sequence of which suggests that they are pseudogenes. *TCHHL1*, one of the 3 candidate SFTP genes of the platypus, has an open reading frame distinctly shorter than that of other *SFTP*s (Supplementary Fig. S1). Due to the possibility of errors of sequencing or genome sequence assembly, corrections of sequences may become necessary in the future^36,39^.

**Figure 2:**
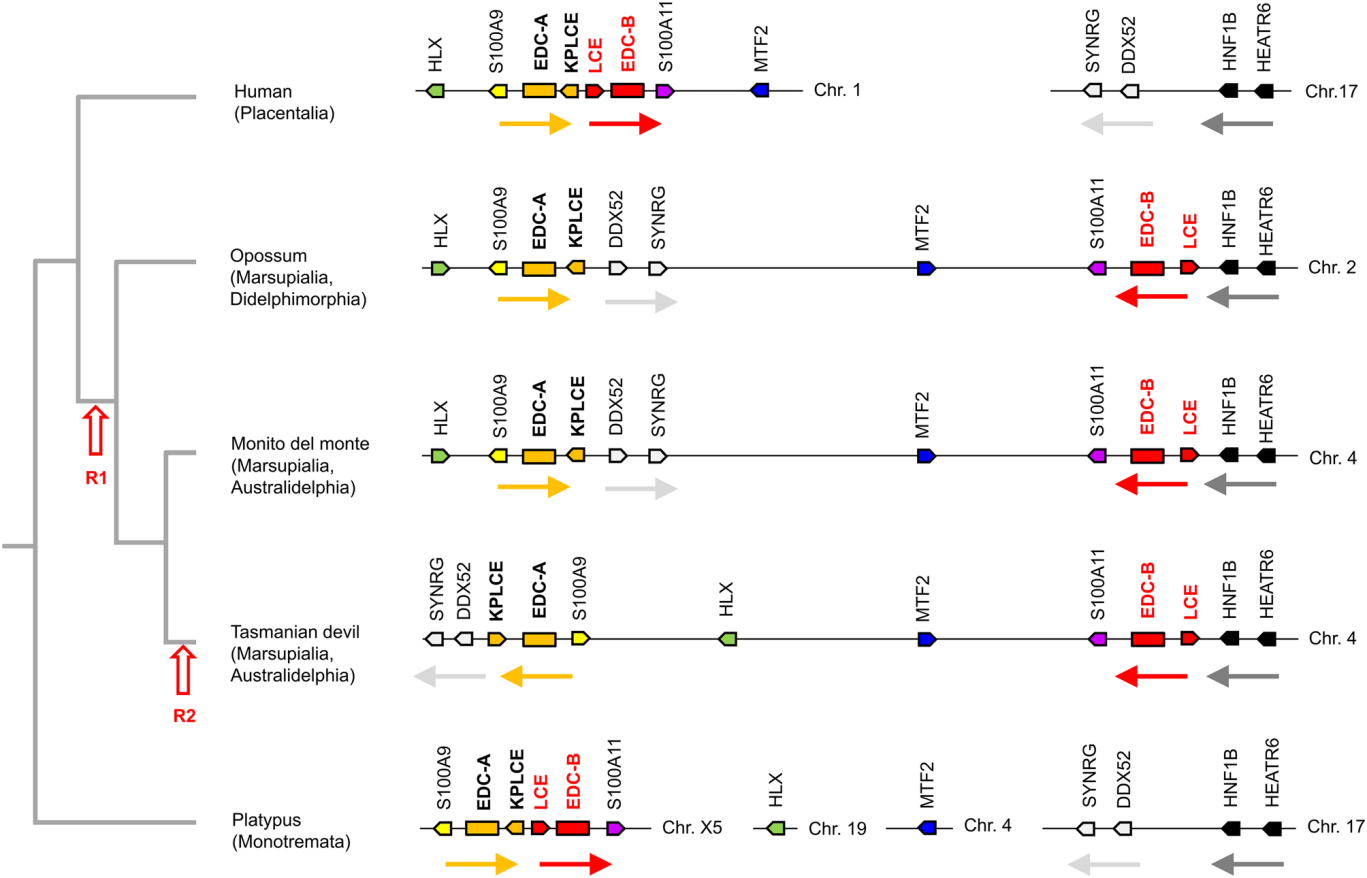
Rearrangement of the EDC in marsupials. Schematic overview of the EDC and neighboring genes of marsupials compared to orthologous genes of human and platypus. Genes are illustrated as arrows with black frames pointing in the direction of transcription. Rectangles represent segments of the EDC. Colors highlight orthology of genes and EDC segments. Arrows below groups of genes indicate the orientation of these conserved chromosomal regions. Recombination events (R1 and R2) of the EDC are indicated on a phylogenetic tree of species, shown on the left. Species: Human (*Homo sapiens*), monito del monte (*Dromiciops gliroides*), opossum (*Monodelphis domestica*), Tasmanian devil (*Sarcophilus harrisii*), platypus (*Ornithorhynchus anatinus*). Chr., chromosome.

Remarkably, the opossum^13^ and other marsupials (Fig. 2) (Supplementary Fig. S3) do not have a continuous EDC, but two EDC segments that are separated by non-EDC genes. The first EDC segment (denoted EDC-A in Fig. 2) is flanked by *S100A9* and contains EDC genes up to *KPLCE*. The second EDC segment (denoted EDC-B in Fig. 2) is flanked by *S100A11* contains *LCE*s and *SFTP*s (Fig. 2). Synteny analysis of the EDC and genes located on both sides of the EDC suggests a model for the evolution of the EDC in marsupials that is depicted on the left of Fig. 2. According to this model, the EDC split in two segments in stem marsupials, and a second recombination event changed the relative orientation of the EDC segments in a subclade (Australidelphia) of marsupials, including the Tasmanian devil (Fig. 2).

### Major types of SEDC proteins previously characterized in placentals are conserved in monotremes

**Figure 3:**
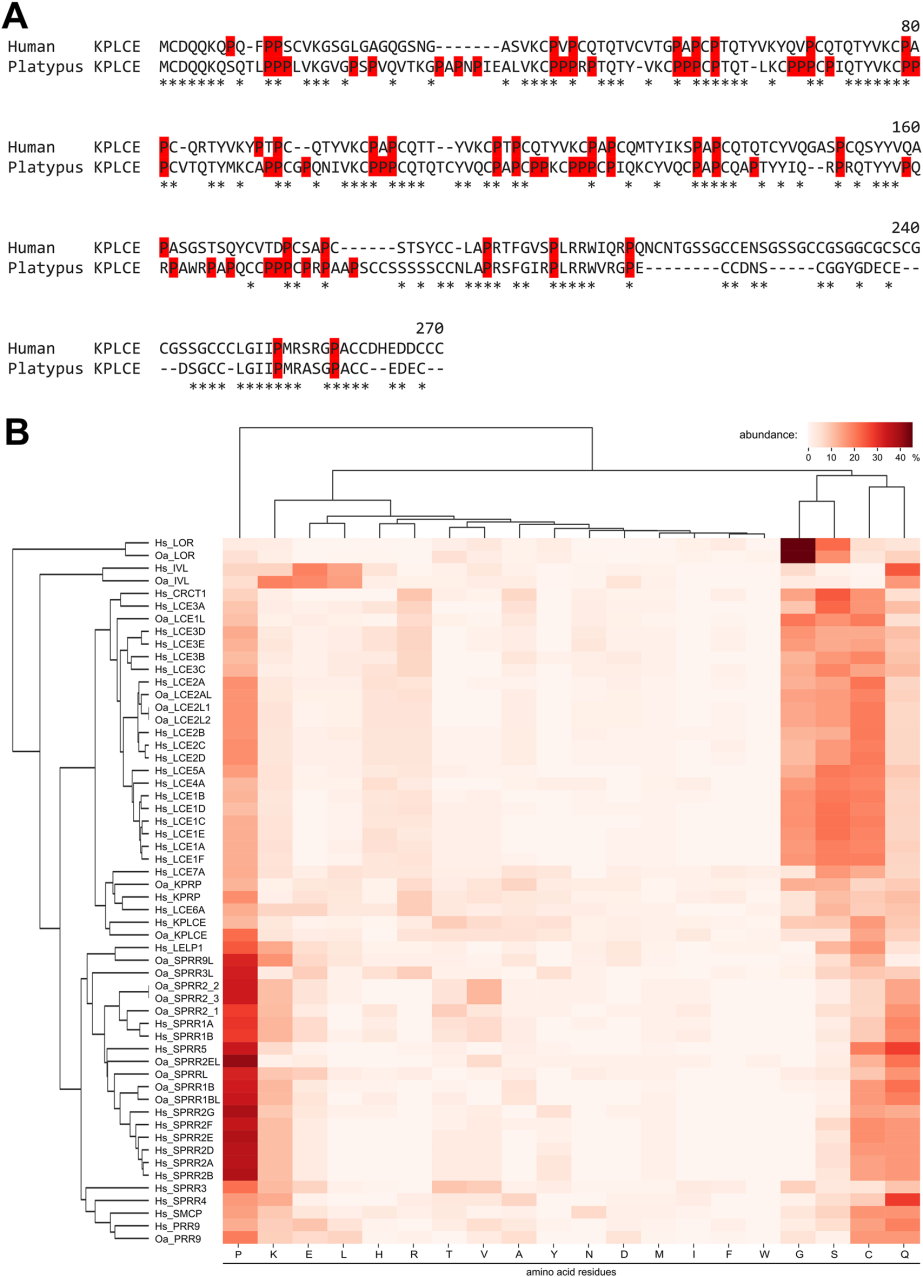
Conservation of SEDC genes in placentals and monotremes. (**A**) Amino acid sequence alignment of *KPLCE* proteins of human and platypus. Amino acid identity is marked with an asterisk, and proline (P) is highlighted in red. (**B**) Clustermap of the SEDC amino acid composition in placentals and monotremes, visualizing the similarities of amino acid composition of the SEDCs. The amino acid residues of the complete SEDCs were used for construction of the clustermap. Species: Human (Hs, *Homo sapiens*) and platypus (Oa, *Ornithorhynchus anatinus*).

SEDC genes predominate by numbers in the EDC of all species investigated. The conserved SEDC genes among monotremes and therian mammals are *LOR*, *IVL*, *PRR9*, *KPRP* and *KPLCE* (*KPRP N-terminal and LCE C-terminal like protein*). KPLCE is a protein previously termed C1orf68, LEP7, or XP32^6^. KPLCEs of human and platypus display 47% amino acid sequence identity (Fig. 3A). Furthermore, the *SPRR* and *LCE* gene families are conserved in all species examined, although with a variable number of genes belonging to these families.

As a reliable phylogenetic analysis based on sequence alignments is not feasible for most SEDC proteins because of their low sequence complexity and presence of multiple sequence repeats^6,33^, SEDCs are classified based on their amino acid composition (Fig. 3B). Based on hierarchical clustering according to amino acid contents (Fig. 3B), the SEDCs of platypus and human can be roughly subdivided in proteins highly enriched for G and S (loricrin), Q and E (involucrin), G, S, C and P (CRCT1, LCEs, KPRP, KPLCE), and P, C and Q (SPRRs, LELP1, SMCP, PRR9). Monotremes and humans have orthologs of each SEDC gene or gene family, with the exception of *Cysteine-rich C-terminal 1* (*CRCT1*), *Late cornified envelope like proline rich 1* (*LELP1*) and *Sperm mitochondria associated cysteine rich protein* (*SMCP*).

### *CRCT1*,* LELP1* and* SMCP* evolved in therian mammals after the divergence from monotremes

**Figure 4:**
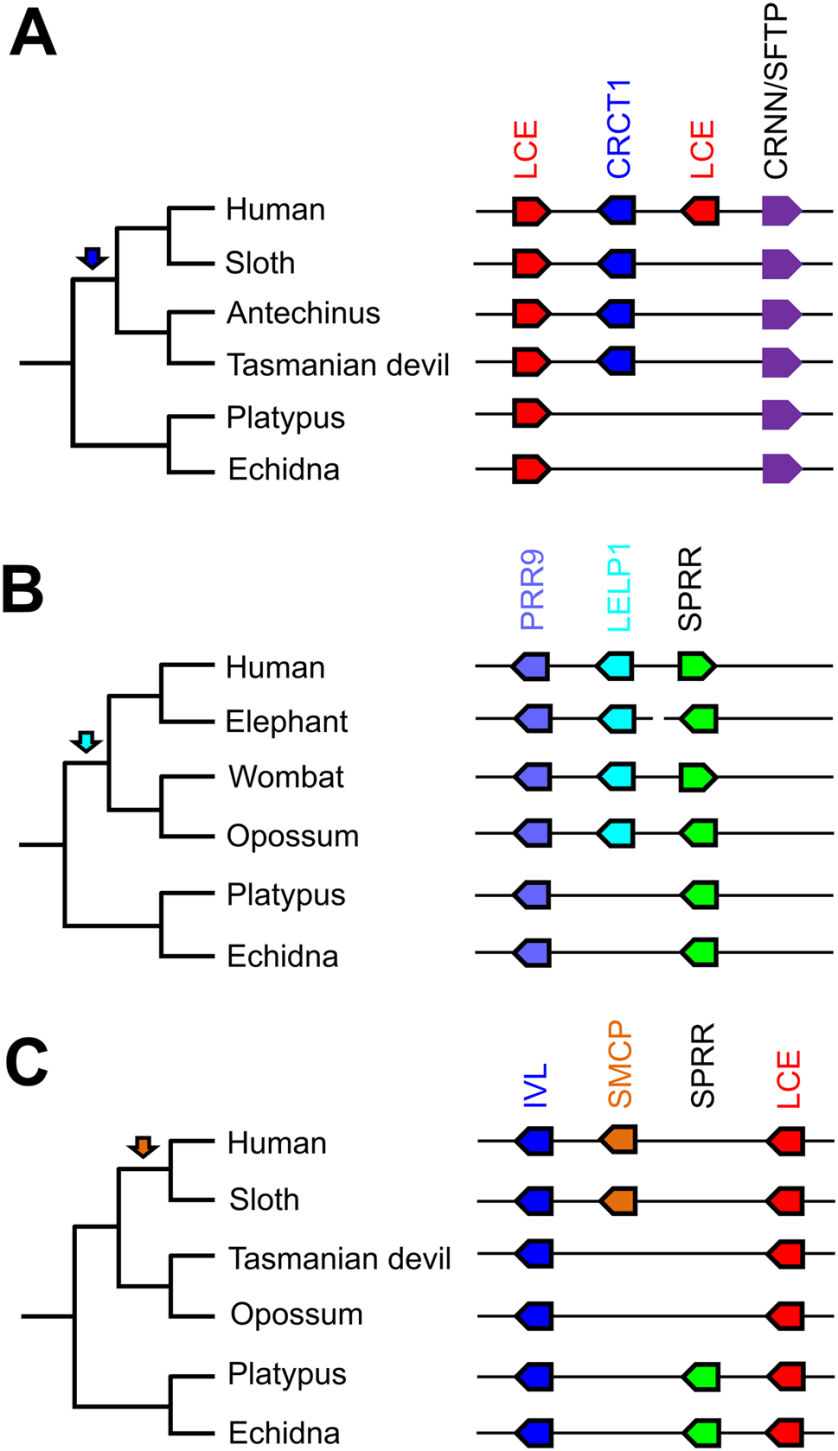
Comparison of *CRCT1*, *LELP1* and *SMCP* loci of monotremes to marsupials and placentals. *CRCT1*, *LELP1* and *SMCP* are absent in the last common ancestor of therian and placental mammals, respectively. (**A**) Schematic overview of *CRCT1* locus. (**B**) Schematic overview of *SMCP* locus. (**C**) Schematic overview of *LELP1* locus. Cladograms show the relation of the investigated species and arrows indicate the origin of the genes in the respective color. Species: Human (*Homo sapiens*), sloth (*Choloepus didactylus*), elephant (*Elephas maximus indicus*), opossum (*Monodelphis domestica*), antechinus (*Antechinus flavipes*), Tasmanian devil (*Sarcophilus harrisii*), wombat (*Vombatus ursinus*), platypus (*Ornithorhynchus anatinus*) and echidna (*Tachyglossus aculeatus*).

To determine the time of the evolutionary origin of *CRCT1*, *LELP1* and *SMCP*, which are absent from the EDC of monotremes (Fig. 1), we extended the comparative analysis of subregions of the EDC to other mammalian species (Supplementary Table S4) and mapped the presence or absence of genes onto the phylogenetic tree of mammals (Fig. 4).

*CRCT1* is absent not only from EDC of monotremes, but also from the EDC of the opossum (Fig. 1). However, other marsupial species like the antechinus (*Antechinus flavipes*) and the Tasmanian devil (*Sarcophilus harrisii*) have a *CRCT1* ortholog (Fig. 4A), suggesting that *CRCT1* originated in a common ancestor of therian mammals (marsupials and placentals), and that it was lost in the lineage leading to the opossum. *LELP1* is present in the EDC of representatives of marsupials and placentals, suggesting that it was also present in the last common ancestor of therian mammals (Fig. 4B). *SMCP* is present neither in monotremes nor marsupials, but present in both humans and the sloth (*Choloepus didactylus*), a basal placental mammal^41^ (Fig. 4C). This distribution in extant species indicated that *SMCP* originated after the divergence of placentals from other mammals.

### The content of SFTP genes differs between monotremes and other mammals

**Figure 5:**
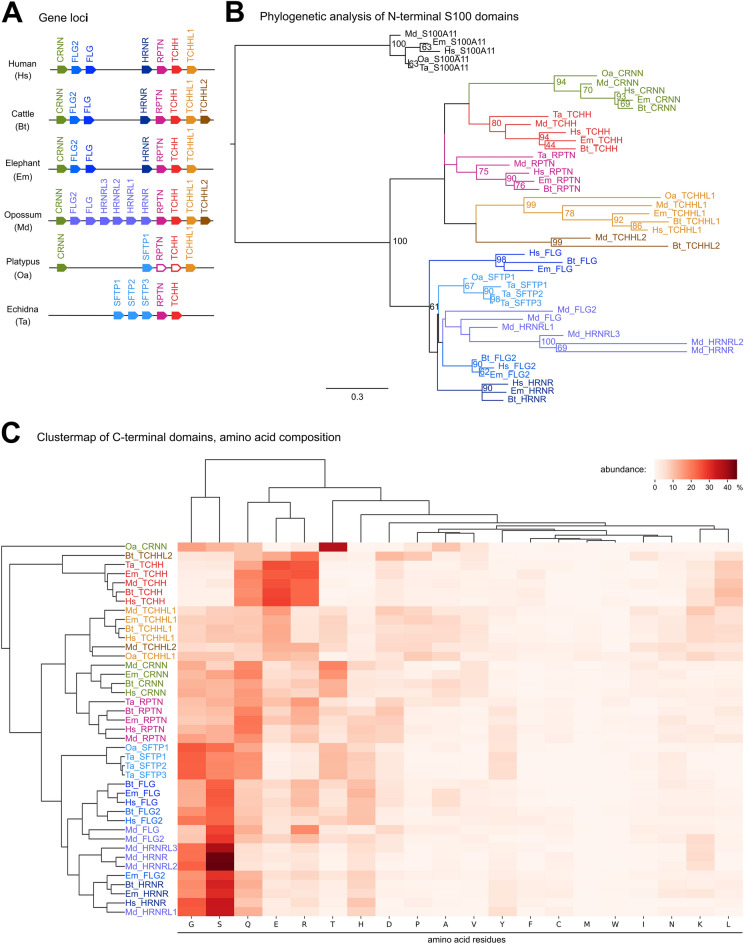
Comparison of SFTP genes of monotremes to placentals and marsupials. (**A**) Schematic overview of SFTP genes in placentals, marsupials and monotremes. Filaggrin is absent in monotremes. White arrows indicate genes with a disrupted coding sequence by either premature stop codons or frameshifts. (**B**) Phylogenic analysis of placental, marsupial and monotreme SFTPs. The S100 domains were used for the construction of the phylogenetic tree and *S100A11* was used as outgroup. (**C**) Clustermap of the SFTP amino acid composition in placentals, marsupials and monotremes, showing the similarities of amino acid composition among the investigated SFTPs. The amino acid residues of the C-terminal ends of SFTPs after the S100 domains were used for the construction of the clustermap. Orthologues are marked in the same color and *FLG* and *FLG*-related proteins are highlighted in different shades of blue. Species: Human (Hs, *Homo sapiens*), cattle (Bt, *Bos taurus*), elephant (Em, *Elephas maximus indicus*), opossum (Md, *Monodelphis domestica*), platypus (Oa, *Ornithorhynchus anatinus*) and echidna (Ta, *Tachyglossus aculeatus*).

SFTPs of monotremes, marsupials and placentals were analyzed by comparing gene arrangements (synteny) in the genomes of representative species (Fig. 5A), phylogenetics of the amino-terminal S100 domain (Fig. 5B; Supplementary Fig. S4) and cluster analysis of amino acid compositions of the carboxy-terminal domain of low sequence complexity (Fig. 5C). We found that SFTP genes display considerable differences between monotremes, marsupials and placentals (Fig. 5). Three representatives of Placentalia, i.e. human, cattle and elephant, have a conserved arrangement of SFTP genes, with bovine *TCHHL2* being the only SFTP that is not conserved in the other species (Fig. 5A). All SFTPs including *TCHHL2* have been annotated in the genome of the opossum, with 3 additional copies of *HRNR* (*HRNR-like 1–3*) being present between *FLG* and *HRNR* (Fig. 5A). Monotremes have fewer SFTP genes than therian mammals, with 5 SFTPs in the echidna and 3 apparently functional and 2 pseudogenized SFTPs in the platypus (Fig. 5A; Supplementary Fig. S5). Remarkably, the types of SFTPs differ substantially between echidna (*SFTP1*, *SFTP2*, *SFTP3*, *RPTN*, *TCHH*) and platypus (*CRNN*, *SFTP1*, *TCHHL1*).

The phylogenetic analysis of the S100 domains showed good support for orthology of CRNN and TCHHL1 (bootstraps > 0.9) in monotremes, marsupials and placentals (Fig. 5B). Orthology of TCHH and RPTN in monotremes and therian mammals was less well supported by this phylogenetic analysis (Fig. 5B), but received independent support from conserved synteny (Fig. 5A) and cluster analysis of the carboxy-terminal domain (Fig. 5C). The remaining SFTPs of monotremes (echidna *SFTP1*, *SFTP2*, *SFTP3* and platypus *SFTP1*) are located at the same position as the group of *FLG2*, *FLG* and *HRNR* in placental mammals and *FLG2*, *FLG*, *HRNR*, *HRNRL1*, *HRNRL2* and *HRNRL3* in the opossum (Fig. 5A), with which they were also grouped by phylogenetic analysis of S100 domains (Fig. 5B) and cluster analysis of the carboxy-terminal domain (Fig. 5C). However, none of the SFTPs of monotremes was identified as a 1:1 ortholog of human FLG, which is a major skin barrier protein^42,43^, or of the human proteins FLG2 or HRNR. The analysis of the sequence repeats in the carboxy-terminal domains suggested that human filaggrin differs clearly from FLG2 and HRNR, and monotreme SFTP1 proteins have sequence repeats similar to those of FLG2 and HRNR (Fig. 5A; Supplementary Fig. S6).

### The gene encoding the filaggrin-processing protease, caspase-14, is amplified in the echidna

**Figure 6:**
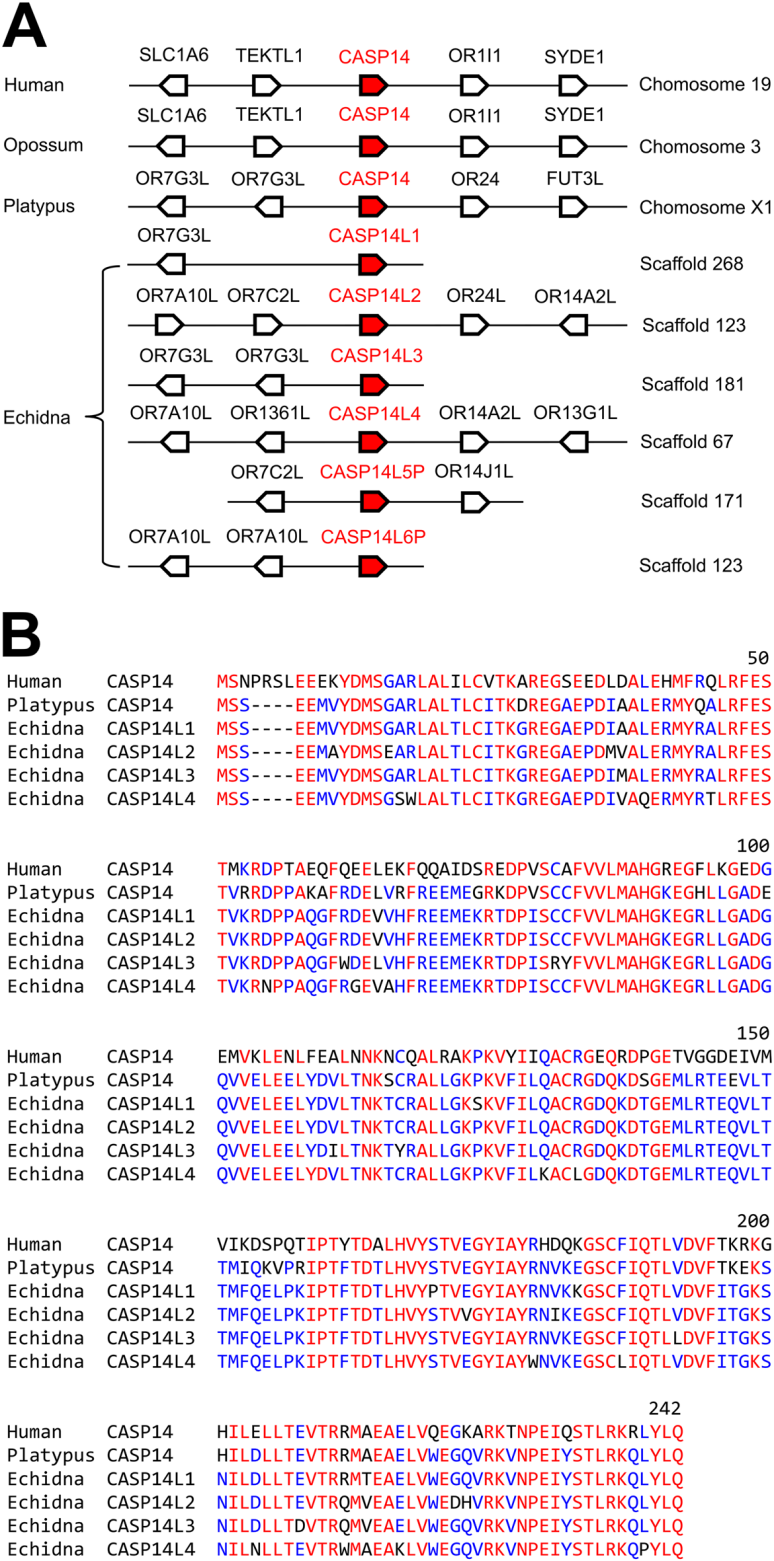
The echidna has multiple copies of *CASP14*. (**A**) Schematic overview of the *CASP14* loci in human, opossum, platypus and echidna. *CASP14* and neighboring genes are depicted as arrows pointing in the direction of transcription. The *CASP14* genes of the echidna have not yet been annotated to chromosomes. Scaffold numbers refer to genome assembly mTacAcu1.pri (accession number GCF_015852505.1). Genes flanking *CASP14* are labeled with gene symbols. OR means olfactory receptor. (**B**) Amino acid sequence alignment of caspase-14 proteins of human, opossum, platypus and echidna. Amino acid residues conserved in all species are colored in red and amino acid residues conserved in > 50% of the examined species are highlighted in blue. Species: human (*Homo sapiens*), opossum (*Monodelphis domestica*), platypus (*Ornithorhynchus anatinus*) and echidna (*Tachyglossus aculeatus*). Accession numbers: Human caspase-14, NP_036246.1; opossum caspase-14, NP_001087242.1; platypus caspase-14, XP_028906221.1; echidna caspase-14-like 1, XP_038598963.1; echidna caspase-14-like 2, XP_038598364.1; echidna caspase-14-like 3, XP_038598727.1; caspase-14-like 4, XP_038599033.1.

Filaggrin is proteolytically processed during cornification of human and mouse keratinocytes. Aspartic peptidase retroviral like 1 (ASPRV1)^44,45^ and caspase-14 (CASP14)^46,47^ were reported to cleave filaggrin^48,49^. No other substrates of ASPRV1 and CASP14 have been identified yet. In monotremes, both ASPRV1 (Supplementary Fig. S7) and CASP14 (Fig. 6, Supplementary Table S5) are conserved despite absence of a 1:1 ortholog of FLG. The echidna has 6 copies of the *CASP14* gene (Fig. 6A), of which 3 encode for proteins that contain all residues required for catalytic activity, 1 copy encodes a protein with peculiar amino acid residue changes close to the catalytic site (Fig. 6B) and 2 gene copies are pseudogenes (Supplementary Fig. S8). Additional “caspase-14-like” genes of the echidna correspond to the orthologs of *CASP15*^50^ and *CASP16*^51^, which are not associated with keratinocyte differentiation. The discordance between the evolutionary trajectories of filaggrin, ASPRV1 and CASP14 suggests that their roles in mammalian epidermal differentiation are not strictly interdependent.

## Discussion

**Figure 7:**
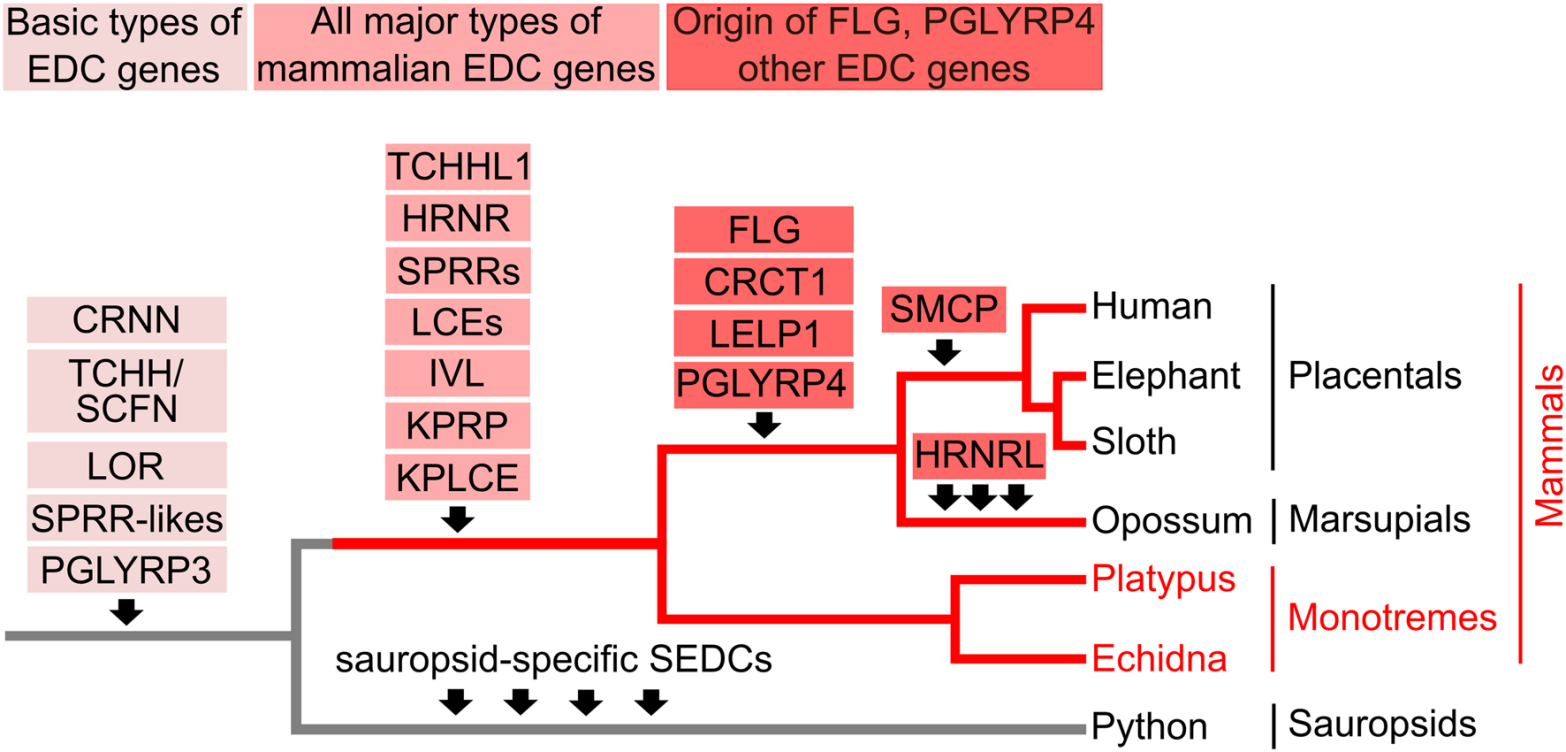
Schematic model of EDC gene evolution in mammals. The origins of genes are indicated with arrows on the simplified phylogenetic tree of amniotes, which also shows the relation of the investigated species. Red branches indicate mammals. Red fonts indicate newly investigated species. Species: Human (*Homo sapiens*), elephant (*Elephas maximus indicus*), sloth (*Choloepus didactylus*), opossum (*Monodelphis domestica*), platypus (*Ornithorhynchus anatinus*), echidna (*Tachyglossus aculeatus*) and python (*Python bivittatus*).

This research closes an important gap in the comparative analysis of the EDC by revealing the organisation and gene composition of the EDC in phylogenetically basal mammals, i.e. monotremes. Previous studies have demonstrated that homologs of *LOR*, *CRNN*, *TCHH*, *PLGYRP3* and possibly *SPRR*s are present in the EDC of sauropsids^13–18,22^, indicating that these genes have originated in stem amniotes. The results of this study show that *TCHHL1*, *IVL*, *PRR9*, *KPRP*, *KPLCE*, and *LCE*s are present in monotremes, suggesting that they have originated in stem mammals (Fig. 7). By contrast, *LELP1*, *CRCT1*, *SMCP* and *PLGYRP4* are not present in the EDC of monotremes, pointing to evolutionary origins in therian mammals. The important human skin barrier gene *FLG* does not have a single-gene ortholog, but members of a phylogenetically defined group of *FLG*-related genes are present in monotremes. Thus, the major organisation of the EDC is conserved in monotremes and therian mammals, but some individual EDC genes underwent a complex evolution in early mammals.

The phylogenetic analysis of the EDC is hampered by the fact that reliable multiple sequence alignments, as required for standard methods of molecular phylogenetics, are not possible for protein segments of low sequence complexity present in SEDC proteins and the carboxy-terminal part of SFTPs. In the present study, we have introduced the method of hierarchical clustering of proteins (Fig. 3B) or protein segments (Fig. 5C) according to amino acid contents as an approach for analyzing the relationships of EDC proteins. This approach aims to take into consideration protein features that appear to be evolutionarily conserved without strictly depending on specific positions within intrinsically disordered proteins, such as many of the EDC proteins^23,24^. For example, glutamine and lysine residues are sites of transglutamination, and cysteine residues are sites of disulfide bonds. Arginine residues are deiminated in an enzymatically catalyzed reaction known as citrullination, which causes the loss of positive charges^6^. The similarity between the phylogenetic tree of the N-terminal S100 domain (Fig. 5B) and the tree generated by cluster analysis of the C-terminal segment (Fig. 5C) of SFTPs seems to support the validity of this approach, but its conceptual foundation needs to be improved.

All well-characterized SEDC genes of humans have homologs in monotremes, suggesting that their function as components of the cornified envelope evolved in early mammals or even in non-mammalian ancestors in the case of loricrin. Three human SEDC genes lack homologs in monotremes: *LELP1*, *SMCP* and *CRCT1*. The roles of late cornified envelope-like proline-rich 1 (LELP1) and sperm mitochondria-associated cysteine rich protein (SMCP) have not been characterized yet^52,53^. Interestingly, the expression levels of both *LELP1* and *SMCP* are much higher in the testis than in the skin (Supplementary Fig. S9A–D), suggesting that they function primarily or exclusively outside of the skin^54,55^. *CRCT1* is expressed in human skin and esophagus, but not in the testis (Supplementary Fig. S9E). The absence of *LELP1*, *SMCP* and *CRCT1* from monotremes indicates that these genes are not required for traits that are shared by all mammals.

The differences in the conservation of SFTP genes in platypus and echidna and the ambiguity of phylogenetic relationships of SFTP in monotremes, marsupials and placentals indicate a complex, and yet incompletely resolved, pattern of evolution of SFTPs in early mammals. The difference in the set of SFTPs in platypus and echidna may indicate that either some of the SFTPs are functionally redundant in monotremes or the evolutionary divergence of skin phenotypes, such as the quills in the echidna and the cornified beak in the platypus, was associated with lineage-specific fates of SFTPs. Due to lengths of up to more than 10.000 nucleotides and a highly repetitive organisation, the second coding exon of *SFTP*s is generally prone to errors in sequencing and sequence assembly. Therefore, apparent truncations of the coding sequence of *SFTP*s in the platypus should be investigated further. Accordingly, we consider the evidence for loss of *TCHH* and *RPTN* preliminary. Our study focused on the evolution of filaggrin and filaggrin-like proteins. Molecular phylogenetics suggests that one SFTP of the platypus and three SFTPs of the echidna are equally closely related to filaggrin, filaggrin 2 and hornerin of humans. Analysis of carboxy-terminal sequence repeats indicates that these proteins of monotremes are more similar to hornerin than filaggrin. Interestingly, filaggrin-like SFTPs of marsupials are also more similar to hornerin than to filaggrin, suggesting that a protein with the features characteristic for filaggrin appeared in placental mammals but not in a common ancestor with monotremes or marsupials. We conclude that filaggrin is a member of a subfamily of SFTPs comprised of filaggrin, filaggrin 2 and hornerin, which may be able to functionally substitute each other.

Proteases, such as ASPRV1 and caspase-14, implicated in proteolytically processing of filaggrin appear to target substrate proteins that lack filaggrin-specific sequence features in monotremes. It is likely that substrates, besides filaggrin, of ASPRV1^44^ and caspase-14^46,47^ are conserved in placental mammals including humans. These putative substrate proteins, either encoded by genes of the EDC or unrelated genes, should be considered in future studies. The impact of the lineage-specific duplications of *CASP14* in the echidna is currently not known.

The results of the present study provide the basis for investigations of the spatio-temporal expression patterns of EDC genes in monotremes, which will be important to fully evaluate the functions of these genes. Previously, histological and immunohistochemical studies have shown similarities between the epidermis of monotremes and therian mammals^56–58^, but comprehensive investigations of the molecular structure of the epidermis and specialized skin appendages of monotremes^37,38,59^ remain to be performed. The design of specific probes and antibodies against EDC proteins (this study), keratins^40^ and other epidermal proteins will help to clarify the molecular evolution of the mammalian skin barrier and skin appendages.

## Material and methods

### Ethics statement

Genome and transcriptome data were obtained from public databases. This study did not involve investigations of humans or animals.

### Identification of EDC genes in genomic sequences

EDC genes were identified by comparative analysis of genomic region between the genes *S100A9* and *S100A11* in the genomes of platypus (*Ornithorhynchus anatinus*, GenBank accession number NC_041753.1, submitted by the Vertebrates Genomes Project^60^), echidna (*Tachyglossus aculeatus*, GenBank accession number NC_052096.1, submitted by the Vertebrates Genomes Project) and opossum (*Monodelphis domestica*, GenBank accession number NC_008802.1, submitted by Vertebrates Genomes Project). The EDC regions around the loci of *CRCT1*, *SMCP* and *LELP1* were analyzed in the genome sequences of sloth (*Choloepus didactylus*, GenBank accession number NC_051308.1, submitted by the Vertebrates Genomes Project), antechinus (*Antechinus flavipes*, GenBank accession number NC_067401.1, submitted by Nanjing Normal University, China)^61^, Tasmanian devil (*Sarcophilus harrisii*, GenBank accession number NC_045429.1, submitted by Wellcome Sanger Institute)^62^ and wombat (*Vombatus ursinus*, GenBank accession number NW_020954576.1, submitted by MRC Institute of Genetics and Molecular Medicine, University of Edinburgh, UK). Additionally, the SFTP genes in the genome of the elephant (*Elephas maximus indicus*, GenBank accession number NC_064821.1, submitted by the Vertebrates Genomes Project) were analyzed.

All human EDC genes and some EDC genes of the aforementioned species were obtained from available annotations in the genome sequence assemblies in the NCBI GenBank. To identify additional EDC genes, tBLASTn searches were performed using human EDC proteins as queries and default settings of NCBI for parameters, but deactivating the filter for low-complexity regions. Amino acid sequences were determined by translating the open reading frames of the regions identified by tBLASTn searches. The orthology of genes was assessed by using the criteria of belonging to the same group in phylogenetic trees, if available, shared local synteny and best reciprocal sequence similarity in BLAST^34^. Of note, none of these criteria alone was considered sufficient for inferring orthology. Human proteins were chosen as queries in tBLASTn searches, because the human EDC is characterized in more detail than the EDC of any other mammal. However, it is possible and even likely that the search strategy applied in this study does not lead to the identification of the entire set of EDC genes in other species.

### Analysis of amino acid sequences encoded by EDC genes

For amino acid sequence alignments the MUSCLE^63^ or MultAlin^64^ programs were used. The ProtParam software tool at the ExPASy portal was used to calculate amino acid percentages of EDC gene encoded proteins^65^. Amino acid compositions were visualized in a clustermap using the python package seaborn (version: 0.12.2^66^).

### Molecular phylogenetics

Sequences belonging to the SFTP family were collected from NCBI GenBank for each species of interest. Multiple sequence alignments and phylogenetic analyses were performed according to an approach described previously^21^. In brief, a multiple sequence alignment of the amino-terminal S100-domain was used as input matrix for phylogenetic analysis. The amino acid substitution model was calculated with prottest (Version 3.0)^67^, and the best fitting model was HIVb^68^. Maximum likelihood analysis with 100 bootstrap replicates was used to infer the phylogenetic tree which was calculated with PHYML (Version 3.3.20220408). The tree was visualized and edited with FigTree (http://tree.bio.ed.ac.uk/software/figtree/, last accessed on July 28th 2023), and inkscape (version: 1.0.0.0; https://inkscape.org/de/, accessed on July 28th 2023) respectively.

### Supplementary Information

Supplementary Information.

## Data Availability

All data generated or analyzed during this study are included in this published article and its Supplementary Information files. We analyzed genomic nucleotide sequences that are available in GenBank at the following accession numbers: NC_041753.1 (platypus, *O. anatinus*), NC_052096.1 (echidna, *T. aculeatus*), NC_008802.1 (opossum, *M. domestica*), NC_051308.1 (sloth, *C. didactylus*), NC_064821.1 (elephant, *E. maximus indicus*), NC_067401.1 (antechinus, *A. flavipes*), NC_045429.1 (Tasmanian devil, *S. harrisii*), NW_020954576.1 (wombat, *V. ursinus*).
