# Supplementary material for: Comparative genomics of monotremes provides insights into the early evolution of mammalian epidermal differentiation genes
Source: Sci Rep. 2024 Jan 16;14:1437. doi: 10.1038/s41598-024-51926-7 (PMC10791643; doi:10.1038/s41598-024-51926-7)
Supplement: Supplementary file 1 — Supplementary Information. [file 41598_2024_51926_MOESM1_ESM.pdf]

Supplementary Data: Supplementary Tables and Figures

Comparative genomics of monotremes provides insights into the early evolution of mammalian epidermal differentiation genes

Julia Steinbinder, Attila Placido Sachslehner, Karin Brigit Holthaus, Leopold Eckhart

Content

Supplementary Tables S1-S5

Supplementary Figures S1-S9

Supplementary Table S1. Platypus (*Ornithorhynchus anatinus*) EDC genes

Gene name	Genomic DNA accession nr.	CDS start	CDS end	Sequence complete	Expression confirmed by RNA-seq	GenBank Gene ID*	Notes
S100A9	NC_041753.1	1462198	1460138	yes	yes	114808162	intron interrupts CDS: see GenBank
PGLYRP3	NC_041753.1	1471001	1479972	yes	no	114808147	introns interrupt CDS: see GenBank
LOR	NC_041753.1	1489992	1489390	yes	yes	114808056	
PRR9	NC_041753.1	1515784	1515245	yes	no	120638162	
SPRR9L	NC_041753.1	1521102	1520755	yes	yes	114808177	record withdrawn from GenBank
SPPR2EL	NC_041753.1	1533529	1534337	yes	no	120638142	
SPRR3L	NC_041753.1	1535878	1535081	yes	yes	n. a.	not predicted by GenBank
SPRR2_1	NC_041753.1	1549573	1549364	yes	yes	n. a.	not predicted by GenBank
SPRR2_2	NC_041753.1	1558052	1558297	yes	yes	n. a.	not predicted by GenBank
SPRR2_3	NC_041753.1	1570427	1570236	yes	yes	114808057	differently predicted by GenBank
SPRR1BL	NC_041753.1	1580196	1579939	yes	yes	114808057	differently predicted by GenBank
SPRR1B	NC_041753.1	1593099	1592749	yes	yes	114808058	differently predicted by GenBank
IVL	NC_041753.1	1604635	1604015	yes	no	n. a.	not predicted by GenBank
SPRRL	NC_041753.1	1611433	1611188	yes	yes	n. a.	not predicted by GenBank
LCE1L	NC_041753.1	1619089	1618679	yes	yes	n. a.	not predicted by GenBank
KPRP	NC_041753.1	1636928	1638505	yes	yes	114808060	
KPLCE	NC_041753.1	1647438	1646704	yes	yes	100681800	
LCE2L1	NC_041753.1	1655080	1654703	yes	yes	114807948	differently predicted by GenBank
LCE2L2	NC_041753.1	1661991	1662368	yes	yes	100089382	differently predicted by GenBank
LCE2AL	NC_041753.1	1668467	1668096	yes	yes	114807964	
CRNN	NC_041753.1	1695432	1701191	yes	no	n. a.	intron: 1695570-1697501<br>not predicted by GenBank
SFTP1	NC_041753.1	1716442	1725763	yes	yes	n. a.	intron: 1716580-1717441<br>not predicted by GenBank
RPTN	NC_041753.1	1748434	1748571	no	no	n. a.	pseudogene; premature stop codon in first coding exon; differently predicted by GenBank
TCHH	NC_041753.1	1773891	1773965	no	no	n. a.	pseudogene; premature stop codon in first coding exon; differently predicted by GenBank
TCHHL1	NC_041753.1	1786007	1786869	yes	no	n. a.	intron: 1786145-1786401<br>not predicted by GenBank
S100A11	NC_041753.1	1814721	1818318	yes	yes	100089848	intron interrupts CDS: see GenBank

Notes: “Expression confirmed by RNA-seq” refers to RNA-seq peaks shown in the section “Genomic regions, transcripts, and products” of the Gene view in NCBI GenBank. CDS, coding sequence (presence of introns between CDS start and end is indicated in the Notes column); n. a., not applicable. *, GenBank ID refers to the most similar gene prediction in GenBank. Differences between gene predictions in this study and in GenBank are indicated in the column “Notes”.

Supplementary Table S2. Australian Echidna (*Tachyglossus aculeatus*) EDC genes

Gene name	Genomic DNA accession nr.	CDS start	CDS end	Sequence complete	Expression confirmed by RNA-seq	GenBank Gene ID*	Notes
S100A9	NC_052096.1	10922408	10925241	yes	yes	119947338	intron interrupts CDS: see GenBank
PGLYRP3	NC_052096.1	10913655	10902775	yes	no	119947327	introns interrupt CDS: see GenBank
Loricrin	NC_052096.1	10891662	10892564	yes	no	n. a.	not predicted by GenBank
PRR9	NC_052096.1	10858457	10859044	yes	no	n. a.	not predicted by GenBank
SPRR2HL	NC_052096.1	10852258	10852605	yes	no	119947002	differently predicted by GenBank
SPRR2BL1	NC_052096.1	10845371	10845147	yes	no	119947001	differently predicted by GenBank
SPRR2BL2	NC_052096.1	10838431	10838207	yes	no	n. a.	not predicted by GenBank
SPRR2BL3	NC_052096.1	10833395	10833589	yes	no	n. a.	not predicted by GenBank
SPRR2BL4	NC_052096.1	10830687	10830487	yes	no	n. a.	not predicted by GenBank
SPRR3L	NC_052096.1	10828172	10829452	yes	no	n. a.	not predicted by GenBank
SPRR2_1	NC_052096.1	10815743	10815988	yes	no	n. a.	not predicted by GenBank
SPRR2_2	NC_052096.1	10808129	10807854	yes	no	n. a.	not predicted by GenBank
SPRR2_3	NC_052096.1	10799930	10800124	yes	no	n. a.	not predicted by GenBank
SPRR2_4	NC_052096.1	10788457	10788666	yes	no	n. a.	not predicted by GenBank
IVL	NC_052096.1	10763197	10763961	yes	no	119947000	differently predicted by GenBank
SPRRL	NC_052096.1	10755731	10755976	yes	no	n. a.	not predicted by GenBank
LCE1L	NC_052096.1	10745514	10745840	yes	no	n. a.	not predicted by GenBank
KPRP	NC_052096.1	10726118	10724499	yes	no	n. a.	not predicted by GenBank
KPLCE	NC_052096.1	10712308	10713177	yes	no	119946852	
LCE2DL1	NC_052096.1	10703387	10703689	yes	no	119946862	
LCE2DL2	NC_052096.1	10690910	10690647	yes	no	119946821	
LCE2DL3	NC_052096.1	10682762	10682472	yes	no	119946817	
LCE2DL4	NC_052096.1	10675079	10674789	yes	no	119946818	
LCE2DL5	NC_052096.1	10667406	10667092	yes	no	119946815	
LCE2DL6	NC_052096.1	10662950	10662612	yes	no	119946819	
LCE2DL7	NC_052096.1	10658456	10658118	yes	no	119946816	
LCE2DL8	NC_052096.1	10651278	10650964	yes	no	119946857	
SFTP1	NC_052096.1	10598727	10581597	yes	no	119946998	intron: 10598589-10597725 differently predicted by GenBank
SFTP2	NC_052096.2	10568140	10556308	yes	no	n. a.	intron: 10568002-10567141 not predicted by GenBank
SFTP3	NC_052096.2	10542843	10533536	yes	no	n. a.	intron: 10542705-10541843 not predicted by GenBank
RPTN	NC_052096.1	10510100	10505629	yes	no	119946997	intron: 10509962-10508908
TCHH	NC_052096.1	10481535	10474690	yes	no	119946996	intron: 10481397-10479514 differently predicted by GenBank
S100A11	NC_052096.1	10439882	10435252	yes	yes	119947258	intron interrupts CDS: see GenBank

Notes: "Expression confirmed by RNA-seq" refers to RNA-seq peaks shown in the section "Genomic regions, transcripts, and products" of the Gene view in NCBI GenBank. CDS, coding sequence (presence of introns between CDS start and end is indicated in the Notes column); n. a., not applicable. *, GenBank ID refers to the most similar gene prediction in GenBank. Differences between gene predictions in this study and in GenBank are indicated in the column "Notes".

Supplementary Table S3. Opossum (*Monodelphis domestica*) EDC genes

Gene name	Genomic DNA accession nr.	CDS start	CDS end	Sequence complete	Expression, RNA-seq	GenBank Gene ID*	Notes
S100A9	NC_008802.1	187669498	187671576	yes	no	100019177	intron interrupts CDS: GenBank
PGLYRP3	NC_008802.1	187632813	187614636	no	no	100019140	introns interrupt CDS: GenBank
LOR	NC_008802.1	187574345	187575694	yes	no	100019077	differently predicted by GenBank
PRR9	NC_008802.1	187478785	187479243	yes	no	n. a.	not predicted by GenBank
LELP1	NC_008802.1	187457476	187457757	yes	no	103097902	record withdrawn from GenBank
SPRR2EL	NC_008802.1	187191344	187192111	yes	no	107651172	pseudogene
SPRR1AL1	NC_008802.1	187164920	187165447	yes	yes	103097554	differently predicted by GenBank
SPRR2L1	NC_008802.1	187147131	187147334	yes	no	n. a.	not predicted by GenBank
SPRR2L2	NC_008802.1	187133443	187133892	yes	no	n. a.	not predicted by GenBank
SPRR2L3	NC_008802.1	187124163	187124564	yes	no	n. a.	not predicted by GenBank
SPRR2L4	NC_008802.1	187110819	187111073	yes	no	n. a.	not predicted by GenBank
SPRR2L5	NC_008802.1	187102044	187101676	yes	no	n. a.	not predicted by GenBank
SPRR1AL2	NC_008802.1	187095883	187096371	yes	no	n. a.	not predicted by GenBank
SPRR1BL1	NC_008802.1	187000467	187000748	yes	yes	107651345	
SPRR1AL3	NC_008802.1	186984304	186984669	yes	yes	100018715	
SPRR1L	NC_008802.1	186942943	186942530	yes	yes	100018686	
SPRR1BL2	NC_008802.1	186906790	186907146	yes	yes	100018648	
SPRR4	NC_008802.1	186884536	186884775	yes	yes	100018614	
IVL	NC_008802.1	186796173	186797150	yes	yes	103096090	
LCE1F1	NC_008802.1	186746776	186747108	yes	yes	103095325	
LCE1F2	NC_008802.1	186684922	186685410	yes	no	130456908	
KPRP	NC_008802.1	186660356	186662080	yes	yes	107651344	
LCE3L1	NC_008802.1	186643224	186642907	yes	no	n. a.	not predicted by GenBank
KPLCE	NC_008802.1	186622089	186623045	yes	no	100618972	
LCE5AL1	NC_008802.1	498496862	498497263	yes	no	n. a.	record withdrawn from GenBank
LCE1EL1	NC_008802.1	498464042	498464464	yes	no	103098023	record withdrawn from GenBank
LCE1EL2	NC_008802.1	498453465	498453890	yes	no	n. a.	record withdrawn from GenBank
LCE5AL2	NC_008802.1	498423442	498423615	yes	no	130457020	pseudogene
LCE1EL3	NC_008802.1	498413017	498413439	yes	yes	130457019	
LCE2AL1	NC_008802.1	498402949	498403392	yes	yes	103097443	
LCE2DL	NC_008802.1	498393418	498393834	yes	yes	103097328	
LCE2CL	NC_008802.1	498385673	498386089	yes	yes	103097215	
LCE5AL3	NC_008802.1	498358345	498358728	yes	yes	103096878	
LCE1EL4	NC_008802.1	498349205	498349609	yes	yes	107651024	
LCE2AL2	NC_008802.1	498322733	498323047	yes	no	103096569	record withdrawn from GenBank
LCE3L2	NC_008802.1	498287133	498287444	yes	no	n. a.	not predicted by GenBank
LCE3L3	NC_008802.1	498273489	498273800	yes	no	n. a.	not predicted by GenBank
LCE3L4	NC_008802.1	498253326	498253024	yes	no	n. a.	not predicted by GenBank
LCE3L5	NC_008802.1	498244429	498244740	yes	no	n. a.	not predicted by GenBank
LCE3L6	NC_008802.1	498235136	498234834	yes	no	n. a.	not predicted by GenBank
LCE3L7	NC_008802.1	498230068	498230379	yes	no	n. a.	not predicted by GenBank
LCE3CL	NC_008802.1	498221020	498220718	yes	yes	103096160	
CRNN	NC_008802.1	498013410	498009667	yes	no	103095863	intron: 498013272-498012118
FLG	NC_008802.1	497947213	497939263	yes	yes	103106047	intron: 497947075-497945758<br>differently predicted by GenBank
FLG2	NC_008802.1	497812400	497808648	yes	yes	103105843	intron: 497812262-497810556
HRNRL1	NC_008802.1	497763900	497756035	yes	no	100019988	intron: 497763762-497761933
HRNRL2	NC_008802.1	497672244	497660012	yes	no	103105680	intron: 497672106-497670182<br>differently predicted by GenBank
HRNRL3	NC_008802.1	497629422	497621790	yes	no	103093607	intron: 497629284-497627574
HRNR	NC_008802.1	497589690	497585535	yes	no	103093297	intron: 497589552-497588469
RPTN	NC_008802.1	497488086	497484078	yes	no	100019364	intron: 497487948-497486631
TCHH	NC_008802.1	497447799	497443415	yes	yes	100019329	intron: 497447661-497445275<br>differently predicted by GenBank
TCHHL1	NC_008802.1	497398205	497394602	yes	yes	100617572	intron: 497398067-497397050
TCHHL2	NC_008802.1	497363668	497360971	yes	yes	103105566	intron: 497363530-497361832<br>record withdrawn in GenBank
S100A11	NC_008802.1	497330944	497322695	yes	yes	100016326	intron interrupts CDS: GenBank

Notes: "Expression confirmed by RNA-seq" refers to RNA-seq peaks shown in the section "Genomic regions, transcripts, and products" of the Gene view in NCBI GenBank. CDS, coding sequence (presence of introns between CDS start and end is indicated in the Notes column); n. a., not applicable. *, GenBank ID refers to the most similar gene prediction in GenBank. Differences are indicated in the column "Notes".

Supplementary Table S4. CRCT1, LELP1 and SMCP genes

Gene name	GenBank Gene ID	Genomic DNA accession nr.	Gene start	Gene end
Hs_CRCT1	54544	NC_000001.11	152514482	152516008
Cd_CRCT1	119517646	NC_051308.1	113824553	113822998
Sh_CRCT1	n. a.	NC_045429.1	427947250	427947615
Hs_LELP1	149018	NC_000001.11	153203430	153205120
Em_LELP1	126073628	NC_064821.1	189252129	189252434
Vu_LELP1	114049637	NW_020954576.1	2161697	2161996
Hs_SMCP	4184	NC_000001.11	152878322	152885047
Cd_SMCP	n. a.	NC_051308.1	113363067	113362813

Notes: n. a., not applicable; Hs, *Homo sapiens*; Cd, *Choloepus didactylus*; Sh, *Sarcophilus harrisii*; Em, *Elephas maximus*; Vu, *Vombatus ursinus*

Supplementary Table S5. CASP14-like genes of the Australian echidna (*Tachyglossus aculeatus*)

Gene name	GenBank Gene name	Genomic DNA accession nr.	Gene start	Gene end	GenBank Gene ID	Note
CASP14L1	LOC119923804	NW_024044983.1	34094	39458	119923804	
CASP14L2	LOC119922773	NW_024044852.1	891398	886058	119922773	
CASP14L3	LOC119923380	NW_024044901.1	298412	303799	119923380	
CASP14L4	LOC119923953	NW_024045091.1	102697	110405	119923953	
CASP14L5P	LOC119923317	NW_024044893.1	267752	259027	119923317	pseudogene
CASP14L6P	LOC119922765	NW_024044852.1	1065150	1073209	119922765	pseudogene

Notes: The numbers under Gene start and Gene end indicate nucleotide positions corresponding to the start of the first exon and the end of the last exon, as provided in GenBank (last accessed on 27 November 2023).

A

>Oa_LOR (XP_028911301.1)
MSYQQHQTTQPTPIPPVGCGGGSGGGGGGGSGGGSGGCGGGCGGGSGGGSGGGGYFPSQQQTQPQGNLPSGGGGGGGGGGCGG
SSGGGKASGGGSWSGGGVVIGGGGGKSSGGGGSSGGCGGSGSGGSSGGCGGGGGSYTQQTTYVPVQTSGGGCSGGGSSGGGSS
SGGCSGGGSSSGGCSGGGSAPQTQQKQPTSWPTK

>Oa_PRR9 (XP_039766785.1)
MPFHAQQRKQPCLPPPCLQQQQERLKSQEACPPPACKEQEPAKCQDLCPPLCQEQGPAKCPPACQEQGPVKCQDLCPPVCQEQ
GPAKCQDLCPPPCQEQGPAKCQDLCPPPCQEQGPAKCPDLCPPVCLDQGPAKCQDLCPPVCLEQGPAKCQDLCPPECKEQGSV
KCQDLCPPKCQGK

>Oa_SPRR9L
MAEDKDHSSCPPKPTCPATPKCSEPSKVSNCLRKLLGCSAPSPPKCPPRDPPKCPHKCPPKEPPKCPPKEPPKCLPKCQPKEP
PKCLPKCQPAEPPKCPPKEPPKCPPPDKAENP

>Oa_SPPR2EL (XP_039766675.1)
MSYQDQQQVKQPCPPPPPIPQPCPPPVQCPQPCPPPVQCPQPCPPPVQCPQPCPPPAQNPQPCSPTCPEPPVPWQQQE

>Oa_SPRR3L
MSFQHYQCKQVCQPPPPCQPKCPPTSSEPCPPRHPEPYQPQNPESCHPGYPEPSQPRYPEPSYPRYPEPTYPKYPQPYQPRYP
EQNYPKHPEPCQPRYPEPCPPRSPNSRYPEPCHPRHPQPCPPRSPEPCPPRHPEPCHPRHPELCPPRSPEPCPSRHPEPCHPR
RPELCPPRSPEPCPPRHQEPCPPRSPVSGQPHFPEPCPPRSPESCSPGRHRHRPKYPAQCSEPCPLQCPEPRPSQPQQWKCTP
VQPSPCCQGKFPQEYK

>Oa_SPRR2_1
MSQQYQQQTKQPCPPPPVIKEPCPSPPVIKDPCHPKTPEPCHPKTSTPCPHPSQQKAPPTQQVPQTKQK

>Oa_SPRR2_2
MSQQYQQQTKQPCPPPPVIKEPCPPPPVVKEPCLPPPVVKEPCPPAPVVKEPCPPKNPTPCPPTSQQKVPPVQQVPQTKQK

>Oa_SPRR2_3
MSQQAKQPTQVPPKEPCHPKTPAPTPCHPKTPEPCHPKTPTPSPAPTQQKAPPAQQVPKTKQK

>Oa_SPRR1BL
MSQQHQQPCPPTQQTKQPCQPPPKCPEPCQAKCPPPAHQKCPEPCQPKCPPPAQQKCPEPCQPKCPPPAQQKCPEPCQPKCPP
PK

>Oa_SPRR1B
MSQQQQQPCPPLQQTKQPCQPPPKCPEPCHPKCPPPAQQKCPQPCQPKCPPPAQQKCPEPCQPKCPPPAQQKCPEPCQPKCPP
PAQQKCPEPCQPKCPPPAQQKCPPPAQGCQKSK

>Oa_IVL
MSQHVQETCQQQRKQPTVLPPTPRPEPCPPPQMESQLKVPQKQSSQLPGKTPQVKSPNPVQVSPDPHLDQKQGKKVNLKDDQH
LEKERQQLEKELEEKKEQLKQQLEEQLEKELEQKEPKDDQHLEKKQQLLAKELEDKKGGLKQQLEEQLEKELSLKDRKDAQHL
EKKQQLLEKELEDKKERLKQQLEEQLAKELEQKDAKDAQHLEKETQLLEKEMEKKKEQLKKQLEEQLAKELEQKEPKYDQLLE
KEKQLLEKEMEKKKEQLKKQLEEQLEKELEQKGAKDAQQLGKKELLGKGPGEKKQLE

>Oa_SPRRL
MADHQQRKQQNLPPPVSCPEPQPQPKCPEPQPKCPEPQPKCPEPQPKCLEPQPKCPEPQPKCPEPQPNCPSAPGGQSKRPQ

>Oa_LCE1L
MSCQQSQQQCPPPPKCLPSKVPTPSPAPCPAPAPAPAPSCCGSGSGGCCGDSNSSGCCGSGGCCGSNSGGCCGSGGCCGSNSG
GCCGSGGCCGSSSGGCCLTSPFHRRRRRRQRRSGCCGGQRDQECEESGSGCCC

>Oa_KPRP (XP_028911306.1)
MCDHQQLQPCPPVPVCCVKGLGLGPSADQGKIFPGSAPGSAQAQAGFTLCQSSSQVSSTQSQRVSLTPGQDSSVGYQCPPQAG
YVMCQSSSQVSSTQGQDSSAGCQPPCPARTGYETCQSSSSAATFDIQDSGLIQAFQGPGPALASYVHCSPVYYKETYYVPCAA
PCPVHTYYVECPVQTSYVQCPALCQSQTSYVRCPAPCQPQASARSHAALCQPQAPAGSHPALNQPQAPARSRPALDQPQVPPR
RCPGLRPAQSCQWCPPRLPRSASTVLPAPAPGASLGLCGPPRCALQGPWRRCGPKYRAEICSPNYPQQVPPRRPPVRIPPIRR
SVSCSDQQFPVLDPGFSRCRVPSPPPRGSRPSWWDLGAGDTSDLSPKARGSGFGRESGGSGCAREEFQGSGNSGCGQRGECRG
SGNSGGGNGGECQGSGNSGCGRRGEVQEAENSGGSSGGECQGSGNSGGGSGGEYQGSENSGCGCEEAPRGLGNSGGSSCRGED
RLIPGNSECLPEDCKKPANNNCEKQRP

>Oa_KPLCE (XP_028910439.1)
MCDQQKQSQTLPPPLVKGVGPSPVQVTKGPAPNPIEALVKCPPPRPTQTYVKCPPPCPTQTLKCPPPCPIQTYVKCPPPCVTQ
TYMKCAPPCGPQNIVKCPPPCQTQTCYVQCPAPCPPKCPPPCPIQKCYVQCPAPCQAPTYYIQRPRQTYYVPQRPAWRPAPQC
CPPPCPRPAAPSCCSSSSSCCNLAPRSFGIRPLRRWVRGPECCDNSCGGYGDECEDSGCCLGIIPMRASGPACCEDEC

>Oa_LCE2L1
MSCQQNQKQCPPPPQCPQQCQPPPKCPPKCPPKCPPKCPPKCPAPCPAPASCCGSSSGGCCGSSSGGCCGSSSGGCCGSSSGG
CCGSSSGGGGCCLSHHHHRRPRLFHRRRHQSPECCDDNSGCC

>Oa_LCE2L2
MSCQQNQKQCPPPPQCPQQCQPPPKCPPKCPPKCPPKCPPKCPAPCPAPASCCGSSSGGCCGSSSGGCCGSSSGGCCGSSSGG
CCGSSSGGGGCCLSHHHHRRPRLFHRRRHQSPECCDDNSGCC

>Oa_LCE2AL (XP_028910767.1)
MSCQQNQQQGPPPPQCPQQCQPPPKCPPKCPPKCPPKCPPKCPAPCPAPASCCGSSSGGCCGSSSGGCCGSSSGGGGCCLSHH
RPRRSHRLRHRSRQECEDSGSGCCHGSDSGCCHGSGSGCC

B

>Oa_CRNN
MPQLLGNIVGIIQAFNSYARTEGDCTTLSRGELKKLIEREFAEVIVKPYDPETVDTVLHLLDDDADGKVGFTEFLALVFRVAQ
ACYNLQISGPGGACGMGARAQAPTGQQTGGAVTRTQTGSAATGSSSGTTVSGRTGSSVTQVTQAQQGSGGRTVGTGSTTVTST
PSLTTTAATSTQSGTCQTGTSGVAARTSSGQTSGTVTRDQRSSNQQTGTSTTVTGGQTTTSTTTVTGSRAATSGSQVTQGSGS
TGGTTTVTATGSPSQTKTTTTTTGGQTSTGATRDPTPTTTVTTGQTASTASRNPTTTMVTGGQTTTTVTRGPTTTTATTGPTA
PTATGGQTTTTVTGRQSGTCQTAGTPVTQARQASSSQTGGPAAATVTVTRTGTPSQAQTTTTTGGPTTTTATGGQTSTTTTGR
QSSSTTTGSPSTTTATVSQTSPAATTGQTSTTATTRPATTTATGGQTTTTATAGPTTTTVTGSQTSTTGTTDQTSTTATTRPA
TTTATGGQSGTCQTGTSGSQVTPAQQGSSSQTGGPVAATVTVTRTGTPSQTQTTGGQTSTAVTGGRTSTAATGGQTTTTATGG
PTSTTATVSQTSTTATTGQTSTTATTRPATTTATGGQSSTTATPAPTTTTATGSQTSTTATRGTTTTATGGQGGTCQTGTSES
QVTQTQQGSSSQTGGPTAATVTVTRTGTPSQTQSTATSTGGQTSTAVTGGRTTTAATGGQTTTTATGGQTATTATGGQTTTTA
TGTPTTTTATGSQTSTTGTTGQTSTTTTTTRPATTTATGGQASTSGSQVTQTQQGSSSQTGGPTAATVTVTRTGTPSQTQSTAT
STGGQTSTAVTRGRTTTAATGGQTTTTATEGQTATTATGGQTTTTATAGPTTTTVTGSQTSTTGTTGQTSTTATTRPATTTAT
GGQTSTSGSQVTQTQQGSSSQTGGPTAATVTVTRTGTPSQTQSTATSTGGQTSTAVTGGRTTTAATGGQTTTTATGGQTATTA
TGGQTTTTATGTPTTTTATGSQTSTTGTTGQTSTTTTTTRPATTTATGGQASTSGSQVTQTQQGSSSQTGGPTAATVTVTRTGT
PSQTQSTATSTGGQTSTAVTRGQTSTAATGGQTTTSATGGQSTTLPTSTSGSQVTQTQSGSSSQTGGTGTATVTVSRTGTPSQ
TQSTTTSAGGQTSSAVTGGQSTTTVTTGQSSDTRQTSSCGQSGASSSPRSNWRTPEATAQTTTTVTTDGSRPSGCSVTGGEVE
AAAKGAVPAPKEKQGITARGLYSYFKGQKQ

>Oa_SFTP1
MSQLLKSIVTVIDIFYHYTQGDGDCETLSKGELKELLEKEFRPILKHPNDPDTVDVIMQILDRDHDRRVDFTEFLLMVFKLTQ
ACNKVVSKDFCPASGSKQKSGRHTWGQGHPNELKGEDEEGEEGEEEEEDGEEEAGKGDGQSSWSAGEEWGSEPSGGTVKHGHK
GGPEGHTARHGHQGSSGKQKHKSGGKHVGEKQGSKKRGSSRSRTGSLTRQDSEHSEGCGGQGVSWGFGGEECPSGSSQSGACG
KQGGQSGKGQSTSCGGHGSSSGGQSGGCGGQGHGSGAGRRSGGCGGQGHGSGAGRRSGGCGGQGHGSGAGGQSGACGGQGHQS
GSGGHSEYEEQSSSSGRQASGHGGRGFSSGGQSGSSGVKGSGSRGGATRGGSGGQSTYRGGHGFGSRGQSGYEEHWSSSGGQS
TGYGGQSSGSGGQSSYGGHGSGSGGEFTRHGGYGTSSGGQSTSYGGQGSGSGGQSSYGGHGSGSGGKFTSQGGHGTSSGGQST
SYGGKGSGSGGQSSHGGHGSGSGGEFTSQGGYGTSSGGQSTSHGGQGSGSGGQSSHGGQASGSGGKFTSQGGYGTSSGGQSTS
HGGQGSGSGGQSSHGGQASGSGGKFTSQGGHGTSSGGQSTSYGGKGSGSGGQSSYGGHRSSSGGEFTSYGGHGTSSGGQATSH
GGQGSSSGGHSGGCGGQESTSGGQSGGHWTTRSWHSWSDGSDGGRIHNQSWTSESEGTQSQSGSESDPEGQAGKSWTQTQGSG
RGHSGSSTTGRQGHGHYQTWDSRGQGSSQSQAGSTHGQTGSTHGQTTDSYGQQGTSYEQTGYSHGQTGSSHGQTGSSYGQTSG
NYGQTGISHGQSSFSTGERAGRHGSGQSESGRSWYSTTGSRDSSPGQYEGEETHYASSYGQTGTSHGQTADSYGQTGSRHRQT
GSSHRQSGSSHGQSRDSYGQTGTSHGQTGYTHGQTGSSQGQTGDSYRQTGSSHRQTGSSHGQTGSSYGQTTDSYGQQGTSHRQ
SGYSHGQTGSSHGQTGSSHGQTGDSYGQAGTSQGQTGSTHRQTTDSYGQQGTRQGQTGSSHGQAGSSHGQTRDTYRQTGSSHG
QTGSSHGQTGSSYGQTTDSYGQQGTSHRQSGYSQGQTGSSHGQTGSSHEQTGGRYGQRGTSQGQTGSTHGQTTDSYGQQGSSQ
GQTGSSHRQAGSSHGQTRDSYRQTGSSHGQIGSSHGQTGSSYGQTTDSYGQQGTSHRQSGYSQGQTGSSHGQTGSSHGQTGDS
YGQAGTSQGQTGSTHGQTTDSYGQQGTRQGQTGSSHGQAGSSHGQTRDTYRQTGSSHGQTGSSHGQTGSSYGQTTDSYGQQGT
SHRQSGYSQGQTGSSHGQTGSSHEQTGGRYGQRGTSQGQTGSTHGQTTDSYGQQGSSQGQTGSSHGQAGSSHGQTRDSYRQTG
SSHGQTGSSHGQTGSSYGQTTDSYGQQGTSHRQSGYSQGQTGSSHGQTGSSHEQARGSYGQRGTSQGQTGSTHGQTTDSYGQQ
GTRQGQTGSSHGQAGSSHGQSRDSYRQTGSSHGQIGSSHGQTGSSYGQTTDSYGQQGTSHRQSGYSQGQTGSSHGQTGSSHEQ
ARGSYGQRGTSQGQTGSTHGQTTDSYGQQGTRQGQTGSSQGQAGSSHGQTRDSYRQTGSSHGQTESSHGQTGSTHGQTTDSYG
QQGTSHSQSGYSHGQTGSSHGQIGSSHGQTRDSYGQAGTSQGQTGSTHGQTTDSYGQQGSSQGQTGSSHEQAGSRHGQTRDSY
RQTGSSRGQTESSHGQTGSTHGQTTDSYGQQGTSHSQSGYSHGQAGSSHGQTRGSYGQAGTSHGQSGSTHGQTTDSYRQQGTS
QGQTGSSHGQAGSSHGQSRDSYRQTGSSHGQTGSSHGQTGSSYGQTTGSYGQTGISHGERTGRHGSGQSESGRSGYSTTRSRD
SSPGQYEGEETHYASSYGQTGTSHGQTTDSYGQQGTSHSQSGSSHGQNRDSYGQTGTSHSQTGSSHGQTRGTYGQQGTSHGQS
GSSHGQNRDSYGQQGTSHGQSGSSHGQNRDSYGQQGTSHGQSGSSHGQTRDSYGQQGTSHGQTGSSHGQTRDTYGQQGTSHGQ
SGSSHGQTRDSYGQQGTTHGQTGSSHGQNRDSYGQTGTSHSQTGSSHGQTRGTYGQQGTSHGQSGSSHGQNRDSYGQTGTSHS
QTGSSHGQTRGTYGQQGTSHGQSGSSHGQTRDSYGQQGTSHGQSGSSHGQTRDSYGQQGTSHGQSGSSHGQTRDSYGQQGTSH
GQTGSSHGQTTDSYGQQGTSHSQRGYSQGHNGSSHGQTRDSYGQTGTSHGQTGSSHGQSSFSTGERAGRHGSGQSESGTSWYS
TTGSRDSSPGQYEGEETHYASSYGQTGTSHGQTADSYGQTGSRNRQTGSSHRQSGSSHGQSGDSYGQTGTSQGQSGSSHEQAR
DSYGQTRTSHGQTGSSYGQTDSTHGQSGHSQGQTGSTHGQTGSSHRQTGSGYGQSGSSYGQTSYTPGQSSDSWGQSGTSYVQT
GGRGRQRQRSGQSWRHGSYGNLAYDYGESGFGQSGHSRTGSRNSSPGQSGSGRTGRQGSSHGQSSDTYGQSGSSTTGNQGSSY
GQSSDTYGQHGSSTTGNQGASYGQSSDTYGQSGSSTNGKQGSSYGQSTDTYGQSGSSNTGKQGSSHGQSTDTYGQSGSSNTGK

QGSGHGQSTDTNGQSGASESGKQGSSHGQSSDTSGQSWSGHWQSSQSWSQGSFPGQTESSSGWTGTRPSQTEHQAEFSILSRG
SRSSTRTPTSQSGGRRRQESSSSLPASGEGSTPLLLIAGPQRRGSESGNPYIRVRTVSGSFYPSSSTPLYEYIQEQRGLY

>Oa_TCHHL1
MPRLLKSVTDVMEVFHKYAQEDGGQAVLTKDGLRQLLRSELGDILQRSPDGRYVGSEVDLLDANHDGVIDFNEFILLVFGLLN
ACYLDIRSLVRSKSAHQRDPEEPKGGKLGEGNRRRPLYREEGEEEEDEEGRGWYRQQSDAASRNWLTEKEGKAYSASLDPQGM
AQKSNPVAHPPQKMMGKAQSPFICPQRGNNSSPRH

C

>Oa_PGLYRP3
MLRLVVFLLAVGVRFSASVPPAMTSHAPQPSTAHPNATDTLARFETLLGCFRDVFQDPPTITPRAEWGAQAPRCTAPLKTPTP
YLLVHHIAGTDCGAQGSSWCLRQLQDHHTLTNGWCDIAYNFLIMESGEVFEGTGWTVQGHHTAGYNEVALGFAFFTNMTDRAP
SQAALASAQHLISFAVQKRHLSPNYIQPFLFRGEDCLQAPGSTPSGVLGCPTIIPRADWGAKGSMANCRKLDRPAKYVIIIHT
AGQPCTELDSCKELVRGIQDFHVNGRKFCDVGYNFLVGEDGNVYEGVGWDTEGAHTYGYNDIALGVAFLGLFEDKPPNAAALM
AAQRLIRCSVDRDYLDPNYLLVAHSDVINSISPGRATHDIIKTWPHFKG

>Oa_S100A9 (XP_028911801.1)
MEKSLENIINVFHQYSVRVGNPDTLTKRELRQLINKELPNFLKDQQCPADVAKILEDLDSNQDSQLSFEEFVVLITRLTVASH
NKMHENAGSGPGHSHGPGLGESGHGHGHSHGPGHGHSHKH

>Oa_S100A11 (XP_028911439.1)
MAKVILNPTETERCIESLIAVFQRYAGQEGNNCTLSKTEFLKFMNTELAAFSKNQKDPGVLDRMMKKLDLNCDGQLDFQEFLN
LIGGLAQACHTSFTAAPPTAHHQHKKI

Supplementary Figure S1. Amino acid sequences of proteins encoded by EDC genes of platypus. (A) Amino acid sequences of proteins encoded by SEDC genes of platypus. **(B)** Amino acid sequences of platypus SFTPs. **(C)** Amino acid sequences of proteins encoded by other EDC genes of the platypus. To show the peculiar amino acid compositions of SEDCs and SFTPs and the importance for protein cross-linking the following amino acid residues are highlighted: lysine (K) and glutamine (Q) as potential sites of transglutamination; cysteine residues (C) as potential sites of disulfide bonds; glycine (G), proline (P) and serine (S) are highly abundant residues not directly involved in cross-linking. When available, the GenBank accession number is shown behind the protein name. Only the S100A proteins whose genes are flanking *PGLYRP3* and *TCHHL1* are included here. SEDC, single coding exon epidermal differentiation complex; SFTP, S100 fused-type protein; Oa, *Ornithorhynchus anatinus*.

A

>Ta_LOR
MSYQQHQTTQPTPLPPVVCGGGSVGGGGGGVGVCGGGSGGGVYYPSQQPSQPQGIPSGGGSSGCGGGVWSGGSGSGGGGKSIG
GGVSSGGCGGGGWSGGSGGEGKSTGGGGVSSGGCGGGGWSGGSGGGGGGKSIGGGGSSGGCGGGSWSGGSGGGGGGKSIGGGG
SSGGCGGGGWFGGSSSGGGKSIGGGGSSGGCGGGGWSGGSSSGGGKSIGGGGSSGGCGTGGSSGGGGGFYPQQPTYVPVQPSG
GGCLTTGGGSSSGGCFGGGSISGGGSSSGGCFGGGSAPQTQQTQPPSWPTK

>Ta_PRR9
MPFHSQQHKQPCLPPPCLQQQQERLKSQKACPQLACQEQGPAKCQDLCPPVCQEQGPAKCPPACPEQGPVKCQDLCPPVCQKQ
GPAKRQDLCPPPCQEQHPAKCQDLCPPPCQAQGPTKCQDLCPPPCQEQGPAKCQDLCPPPCQEQGPAKCQDLCPPVCLEQGPA
KCQDLCPPEFKEQGPVKCQDLCPPKCQGK

>Ta_SPRR2HL (XP_038624405.1)
MAEDKDHSSCPPKPICPATPKCSEPSKVSNCLRKLLGCSAPSPPKCPPKDPPKCPPKCPPKEPPKCPPKEPPKCLPKCPPKEP
PKCLPKCQPKEPPKCPPKDPPICLPPDKAENP

>Ta_SPRR2BL1
MSYQNQQQVKQPCPPPPPIPPPCPPPVKCPQPCPPPVKCPQPCPPPVKCPQPCPPPVKYLPKCQEPCPPPPCQQ

>Ta_SPRR2BL2
MSYQDQQQVKQPCPPPPPIPPPCPPPVKCPQPCPPPVKCPQPCPPPVKCPQPCPPPVKYLPKCQEPCPPPPCQQ

>Ta_SPRR2BL3
MSYQDQQQVKQPCPPPPPIPQPCPPPVKCPQPCPPPVKCPQPCPPPVKYLPKCQEPCPPPPCQQ

>Ta_SPRR2BL4
MSYQNQQQVKQPCPPPPPIPQPCPPPAKCPQPIPPSVQCPQPFPPPVQCPQPCPPACQEPPLPCQQ

>Ta_SPRR3L
MSFQQNHFKQVCQPPPPNQPKCPRRQPEPYHPQYPEPSYPKLPEPCQPRYPESFPPRSPNLRYPEPCHPRHPGPCPPRSPGPC
PPRHPEPCHPQYPEQYPPRSLEPCHPRHPEPCPPRSLEPYPPRHPEPCHPQHPEPCPPGSPGPCTPRHPEPCHPQYPQLYPPR
SPKPCHPRHPEACPPRSPGPCPPRHPEPCHPQYPELYPPRSPEPCHPRHPESCPPRSPGPCSPRHPKPCHTQYPELHPPRSPE
PCHPRHPEPCPPRSPGPCPPRHPEPCHPQYPEPCPPRSPEPCPPRQAEPCHLQYPELYPPRSTGPCPPRHPEPCYPQYPELYP
PRSPEPWPPRHPEPYPPRSSVPCQPHFPEPYPPRSPEPCSPGRNRRHPKNPALCPEPCPPQCPEPRPSQPQQWDAPLIVQPSP
CCRGKFPREYK

>Ta_SPRR2_1
MSQQYQQQTKQPCQPPPIVKEPCPPPPVVKEPCLPPPVVKEPCLPPPVVKEPFLPKTSTPCPPPSQQKLPPAQQVPKTKQK

>Ta_SPRR2_2
MSQQYQEQTKQPCQPPPIVKEPCPPPPVVKEPCPPPPVVKEPCLPPVVVKEPCPPPVVVKEPFPPKTSTPCPPPSQQKVPPAQ
KVPQTKQK

>Ta_SPRR2_3
MSQLQAKQTTQVPPKEPCKLKIPTPTLCHPKTPKLSQPKPCLLCPAPAQQKAPPAQQVPKSKQK

>Ta_SPRR2_4
MSQQHQQSCPPPQQTKQQCQRPPKCPEPCQPKCLPPAQQKCPEPCQPKCPPPAQQKCPGPCQPKCPLPK

>Ta_IVL
MSQPVQEPCQQQCKQPTVLPPALRPEPGPPPQMEPQLKVPQKPPTELPGKTPQVKSPIPVQVSSDPQPDQKEGEKEDQTDDPS
PQPDQEEGKKEDQSDDSSPQPEQTEGKKEDQMDDQHLEKEKQLLEKELEEKKEHLKKQLEEQLEEKLKLKDPEDDQNLEKEKQ
LLEKELEEKKEHLEKELEQEEPKDDQNLEKEKQLLEKELEDKKEQLKKQLEEGLEKELEQKEPKDDQHLEKEKQLLEKELEEK
KQQLE

>Ta_SPRRL
MADQQQRKQQNLPPPVSCPEPQPQPKCPEPQPKYPEPQPKYPEPQPKCPEPQPKYPEPQPNYPEPQPKCPSAPGGHSKRPQ

>Ta_LCE1L
MSYQQNQQQCPPLPKCLPPKVPTPCPAPCPAPVPAPTSSCCGSGSGGCCGDSNSRGCCGSGGCCGSSSGGCCLTSPFHRRRRR
RQRRSGCCGGQRDQECEESGSGCCC

>Ta_KPRP
MCDHQQLQPCPPVPVCCVKGLGVGPSVDQGKIFPCPAPGPAQAQAGFTLCQSSSQVSSAQSQQVSLTSGQDSSVGYQCPAQAG
SVICQSSSQVSSTQGQDSSAWCQPPCPTQASYGTCQSSSSAATFDIQDSALIQTFQGSGPALASYAQCSPVYYKETYYVPCPA
PCPVHTYYVECPVQTSYVQRPVLCQSQTSYVRCPAPCQPQVSVGSHAALCQPQAPAGSRPALYQTEAPARSRPALDQPQVPPR
RCPGLRPAQTCQWCPPRPPRSASAALPAPAPGTSLGLCGPPRCALQGPWRRCGPKYRAEICSPNYPQQVAPRRPPVRIPPIRR

SVSCSDRQFPVLDTGFSNCRVPSPPPRGSRPACWDLGAGDTSDLCPKARGFGFCQESGGSGCGREEFQGSGYSGCGQRGEFRG
SGNSGWGSGRECQGSWNFGCSRREEVQGSKNSGGGCGGEGQGSGNSGCSRGGKLQGSGNSGGGSGGEFQGSENFSCGCKEAPQ
RLGNSGGSISRGEDCLIPGNSECLPEDCKEPAKTNCEEQRP

>Ta_KPLCE (XP_038624254.1)
MCDQQKQPQTFPAPVAQVKGVGPGPVQVTKCPAPNPIEALVKCPPPCPAQTYVKCPPPCPTQTFVKCPPPCPTQTYVKCPPPC
PTETLVKCPPPCPTQTYVKCPPPCPTQTYVKCPPPCVTQNIVKCPPPCQTQTCYVQCPAPCPPKCPPPCPIQKYYVQCPPPCQ
VPTCYIQRPSQTYYVPQRPAWRPAPQYCPPPCPRPCPRPCPRPCPRPCPRPRPAAPSCCPSSSNCCNLAPCSFGIRPLRRWVR
GPECCDNSCGGYGDECEDSGCCLGIIPMRASGPACCGDEC

>Ta_LCE2DL1 (XP_038624268.1)
MSWQQNQKQFPPTPQFPQQCQPPPKCPPKCPAPCPAPCPASSSCCGPSSGGCCGPSSGDCCGPSSGGCCLSHHHHRRPRLFHR
RQHQSPDCCDNGSSGCC

>Ta_LCE2DL2 (XP_038624210.1)
MSCQQNQKQCPPPPQFPQKCQPPPKCPPKCPAPCPAPSSCCGPSSGGCCGSSSGGCCLSHHHHHRPHLFHHRRHQSPECCDDN
SDCC

>Ta_LCE2DL3 (XP_038624207.1)
MSWQQNQQQCPPPPQCPQKCQPPPKCPPKCPPKCPPKCPAPCPTLSSCCGPSSGGGCGPSSGGGCCLSHHHHHRPHLFHRHRH
QSPECCDDNSDCC

>Ta_LCE2DL4 (XP_038624208.1)
MSWQQNQQQCPPPPQCPQKCQPPPKCPPKCPPKCPPKCPAPCPTLSSCCGPSSGGGCGPSSGGGCCLSHHHHHRPHLFHRHRH
QSPECCDDNSDCC

>Ta_LCE2DL5 (XP_038624205.1)
MSWQQNQKQFPPTPQFPQQCQPPPKCPPKCPPKCPPKCPAPCPTLSSCCGPSSGGCCGPSSGGGCGPSSGGGCCLSHHHHHRP
HLFHRHRHQSPECCDDNSDCC

>Ta_LCE2DL6 (XP_038624209.1)
MSWQQNQKQFPPTPQFPQQCQPPPKCPPKCPPKCPPKCPAPCPTLSSCCGPSSGGCCGPSSGGGCGPSSGGGCGPSSGGGCCL
SHHHHHRPHLFHRHRHQSPECCDDNSDCC

>Ta_LCE2DL7 (XP_038624206.1)
MSWQQNQKQFPPTPQFPQQCQPPPKCPPKCPPKCPPKCPAPCPTLSSCCGPSSGGCCGPSSGGGCGPSSGGGCGPSSGGGCCL
SHHHHRRPHLFHRHRHQSPECCDDNSDCC

>Ta_LCE2DL8 (XP_038624262.1)
MSWQQNQKQFPPTPQFPQQCQPPPKCPPKCPPKCPPKCPAPCPTLSSCCGPSSGGCCGPSSGGGCGPSSGGGCCLSHHHHHRP
HLFHRHRHQSPESCNDNSDCC

B

>Ta_SFTP1
MSQLLESIATIIDVFYHYTQGDGHCETLSKRELKELLKKEFRPILKNPNDPDKVDAIMQMLDQDHNRRVNFTEFLLLVFKLTQ
ACNKMVSKDFCSASGSKQKSGRHPWGQGHPSQPEGEGEEDEEGEEGEEEEEDGEAEAGRGASQSSWSAGEEWGSEPSGETVRH
GHKARHEHQGGSGKQKHKLGGQYVGEKQGSKKRGSSRFRTESLTKQGSGQSGGCGGQGVSWGFGGEECPSGSNQSGACGRQGG
QSGKGQSTSYIGRGSSTGGQSGRSAGQGHGSGAGDHSDYEQQWSNSGSQTTGHGGHGSISGGQSGSSGGNRSGSRGGATKDGQ
TLDSYGQQETSHGQSGSSHGQTTDSYGQQVTSHSQTGSAHGQTTDSYGQQGTSYGQSGSSHGQTRDSYGQHGTSHGHSGSSHG
QTRGTYGQTGTRQGQTGYSHGQTGSSHGQTGSSQGQTGDSYRQTGSNHGQTGSSHGQTESSYGQATDSHGQQGTSHGQTGYSH
GQTGYSYGQNGSGHGQTRDSYRQGGSSHGQTGSSYGQNTGSYGQQGTSHGQKGYSQGQTGSSHGQTGSSYGQTTDNYGQTGTS
HGQSSYSTGERAGRHGSGQSESGRSGYNTTGSRDSSPGQYEGEETHYGSSYGQTGTNHGQTTDSYGQQGTSHGQSGSRHGQTR
DSYGQQGTSHGQTGSSHGQTRGTYGQTGTRQGQTGSSHGQNTDSYGQTGTRQGQTGSTHGQNTESYRQQGTSHGQSGSAHGQN
TDSYGHQGTSHGQTESSHGQTRDSYGQQGTSHGQSGSRHGQNTDSYGQQGTSHGQTGSSHGQTRGNYGQQGTSHGQSGYSHGQ
NTDSYGQQGTSHGQTESSHGQITDSYGQQGTSHGQSGSSHGQTRDSYGQQGTSHGQTGYTYGQSTDSYGQQGTSHGQSGYSHG
QNTDSYGQQGTSHGQTESSHGQITDSHGQQGTSHGLSGSSHGQTRDSYGQQGTSHGQTGSRHGQTRGYYGQQGTSHGQSGYSH
GQNTDSYGHQGTSHGQSGYSHGQNTDSYGHQGTSHGQIQSSHGQVTDSYGQQGTSHGQSGYSHGQNTDSYGHQGTSHGQIESS
HGQVTDSYGQQGTSHGQSGYSHGQNTDSYGHQGTSHGQIESSHGQVTDSYGQQGTSHGQSGSSHGQTTDSYGQQGTSHGQTGS
SHGQTRGNYGQQGNSHGQSGSNRGQTTDSYGQQGTSHGQTGYTYGQTTDSYGQQGTSHGQSGYSHGQTRDSYGQQGTSHGQTG
SSHGQTRGNYGQQGTSHGQSGYSHGQTRDSYGQQGTSHGQTGSSHGQTRGNYGQQGTSHGQSGSSHGQTRDSYGQQGTSHGQT
GSSHGQTRGNYGQQGTSHGQSGYSHGQNTDIYGQQGTSHGQTGSRHGQTRGNYGQQGTSHGQSGYSHGQNTDSYGHQGTSHGQ
LGYSHGQNTDSYGQQGTSHGQSGYSHGQNTDSYGHQGTSHGQSGYSHGQNMDSYGHQGTSHGQIQSSHGQVTDSYGQQGTSHG
QSGYSHGQNTDSYGQQGTSHGQIESSHGQVTDSYGQQGTSHGQSGYSHGQNTDSYGHQGTSHGQIESSHGQVTDSYGQQGTSH
GQSGSSHGQTRDSYGQQGTSHGQTGSSHGQTRGNYGQQGNSHGQSGSNRGQTTDSYGQQGTSHGQTGYTYGQTTDSYGQQGTS
HGQSGYSHGQTRDSYGQQGTSHGQTGSSHGQTRGNYGQQGTSHGQSGYSHGQTRDSYGQQGTSHGQTGSSHGQTRGNYGQQGT
SHGQSGSSHGQTRDSYGQQGTSHGQTGSSHGQTRGNYGQQGTSHGQSGSIHGQTRDSYGQQGTSHGQTGSRHGQTRGNYGQQG

TSHGQSGYSHGQNTDSYGQQGTSHGQSGYSHGQNTDSYGQQGTSHGQSGSSHGQTRDSYGQQGTSHGQTGSSHGQNRGNYGQQ
GTSHGQSGYSHGQNTDSYGHQGTSHGQSGYSHGQNTDSYGQQGTSHGQSGSSHGQTRDSYGQQGTSHGQTGSSHGQNRGNYGQ
QGTSHGQSGYSHGQNTDIYGHQGTSHGQTESSHGRITDSYGQQGTSHGQSGSSHGQTRDSYGQQGTSHGQTGSSHGQTRGYYG
QQGTSNGQSGYSHGQNTDSYGQQGTSHGQSGYSHGQNTDSYGQQGTSHGQIESSHGQVTDSYGQQGTSHGQSGYSHGQNTDSY
GHQGTSHGQIESSHGQVTDSYGQQGTSHGQSGSSHGQTRDSYGQQGTSHGQTGSSHGQTRGNYGQQGNSHGQSGSNRGQTTDS
YGQQGTSHGQTGYTYGQTTDSYGQQGTSHGQSGYSHGQTRDSYGQQGTSHGQTGSSHGQTRGNYGQQGTSHGQSGYSHGQTRD
SYGQQGTSHGQTGSSHGQTRGNYGQQGTSHGQSGSSHGQTRDSYGQQGTSHGQTGSSHGQTRGNYGQQGTSHGQSGYSHGQNT
DIYGHQGTSHGQTESSHGRITDSYGQQGTSHGQSGSSHGQTRDSYGQQGTSHGQTGSSHGQTRGYYGQQGTSHGQSGYSHGQN
TDSYGQQGTSHGQSGYSHGQNTDSYGHQGTSHGQIESSHGQVTDSYGQQGTSHGQSGSSHGQTTDSYGQQGTSHGQTGSSHGQ
TRGNYGQQGNSHGQSGSNRGQTTDSYGQQGTSHGQTGYTYGQTTDSYGQQGTSHGQSGYSHGQTRDSYGQQGTSHGQTGYTYG
QTTDSYGQQGTSHGQSGYSHGQTRDSYGQQGTSHGQTGSSHGQTRGNYGQQGTSHGQSGSSHGQTRDSYGQQGTSHGQTGSSH
GQTRGNYGQQGTSHGQSGSSHGQTRDSYGQQGTSHGQTGSRHGQTRGNYGQQGTSHGQSGYSHGQNTDSYGHQGTSHGQSGYS
HGQNTDSYGQQGTSHGQSGSSHGQTRDSYGQQGTSHGQTGSSHGQNRGNYGQQGTSHGQSGYSHGQNTDSYGQQGTSHGQSGY
SHGQNTDSYGQQGTSHGQTESSPGQITDSYGQQGTSHGQSGSSHGQTRDSYGQQGTSHGQTGSSHGQTRGNYGQQGTSHGQSG
YSHGQNTDSYGHQGTSHGQSGYSHGQNTDSYGQQGTSHGQTESSLGQVTDSYGQQGTSHGQSGSSHGQTRDSYGQQGTSHGQT
GSSHGQTRGNYGQQGNSHGQSGSNHRQTTDSYGQQGTSHGQTGYTYGQTTDSYGQQGTSHGQSGYSHGQNTDSYGQQGTNHGQ
TESSHGQITDSYGQQGTSHGQSGASHGQTRDSYGQQGTSLGQTGSRHGQTRGNYGQQGTSHGQSGYSHGQNTDSYGQQGTSHG
QSGYSHGQNTDSYGQQGTSHGQSGSSHGQTRDSYGQQGTSHGQTGSNHGQNRGNYGQQGTSHGQSGYSHGQNTDSYGQQGTSH
GQSGYSHGQNTDSYGHQGTSHGQTESSHGQITDSYGQQGTSHGQSGSSHGQTRDSYGQQGTSHGQTGSSHGQTRGNYGQQGTS
HGQSGYSHGQNTENYGHQGTSHGQSGYSHGQNTDSYGHQGTSHGQTESSHGQVADSYGQQGTSHGQSGSSHGQTRDSYGQQGT
SRGQTGSSHGQTRGNYGQQGNSHGQSGSNHRQTTDSYGQQGTSHGQTGYTYGQTTDSYGQQGTSHGQTGSSHGQITDSYGQQG
TSHGQSGSSHGQTRDSYGQQGTSHGQTGSSHGQNRGNYGQQGTSHGQSGYSHGQNTDSYGQQGTSHGQTESSHGQITDSYGEQ
GTSHGQSGSSHGQTRDSYGQQGTSHGQTGSSHGQTRGNYGQQGTSHGQSGYSHGQNTDSYGQQGTSHGQSGYSHGQNTDSYGH
LGISHGQIESSHGQVTDSYGQQGTSHGQSGSSHGQTTDSYGQQGTSHGQTGSSLGQTRGNYGQQGNSHGQSGSRHGQTRDSYG
QTGTSHGQTGSSHGQSRGTYGQTGTRQGQTGSSHGQTTDSYGQQGTSHGQSGSSHGQTRDSYGQQGTGHGQTGSSHGQNRGNY
GQQGTSHGQSGYSHGQNTDSYGHQGTSHGQTESSHGQITDSYGQQGTSHGQSGSSHGQTRDSYGQQGTSHGQTGSSHGQTRGN
YGQQGTSHGQSGYSHGQNTDSYGQQGTSHGQSGYSHGQNTDSYGHQGISHGQIESSHGQVTDSYGQQGTSHGQSGSSHGQTTD
SYGQQGTSHGQTGSSHGQTRGNYGQQGTSHGQSGYSHGQNTENYGHQGTSHGQSGYSHGQNTDSYGHQGTSHGQTESSHGQVT
DSYGQQGTSHGQSGSSHGQTRDSYGQQGTSHGQTGSSHGQTRGNYGQQGNSHGQSGSNHRQTTDSYGQQGTSHGQTGYTYGQT
TDSYGQQGTSHGQTGSSHGQITDSYGQQGTSHGQSRSSHGQTRDSYGQQGTSHGQTGSSHGQNRGNYGQQGTSHGQSGYSHGQ
NTDSYGQQGTSHGQSGYSHGQNTDSYGHQGISHGQIESSHGQVTDSYGQQGTSHGQSGSSHGQTTDSYGQQGTSHGQTGSSLG
QTRGNYGQQGNSHGQSGSRHGQTRDSYGQTGTSHGQTGSSHGQSRGTYGQTGTRQGQTGSSHGQTTDSYGQQGTSHGQSGSSH
GQTRDSYGQQGTSHGQTGSSHGQNRGNYGQQGTSHGQSGYSHGQNTDSYGHQGTRHGQTESSHGQITDSYGQQGTSHGQSGSS
HGQTRDSYGQQGTSHGQTGSSHGQTRGNYGQQGTSHGQSGYSHGQNTDSYGQQGTSHGQSGYSHGQNTDSYGHQGISHGQIES
SHGQVTDSYGQQGTSHGQSGSSHGQTTDSYGQQGTSHGQTGSSLGQTRGNYGQQGNSHGQSGSRHGQTRDSYGQTGTSHGQTG
SSHGQSRGTYGQTGTRQGQTGSSHGQTTDSYGQQGTSHGQSGYSHGQNTGSYGHQGTSHGQSGSSHGQTRDSYGQQGTSHGQT
GSSHGQTRGDYGHQGTSHGQIESSHGQVTDSYGQQGTSHGQSGYSHGQNTGSYGHQGTSHGQSGSRHGQTRDSYGQQRTSHDQ
SGSSHGQITDSYGQQGTSHGQTGSSKGQTRGTYGQTGTRQGQTGSSHGQNTDSYGQTGTSHGQSSYSTGERTGRHGSGQSESG
RSGYSATGSRDSSPGQYEGEETHYGSSYGQTGTSHGQTTDRYGQTGSSHRQTGSSVGQSGYSHGQTRDSYGQTGASHGQTGSS
DGQTGSTHGQTGHSQGQIGSSHGQTRDIYGQTGSSHRQTETSHGQTGSNQGQSGTSQGQTGSGYGQTGSSYGQTSYSQRQISD
SWAQSGTSSVQTGGRGRQRQRSGQNWRHGSYGNLAYDYGEFGFGQSSYGVTVSRNSSPGQPGSGRTGTQGSSYGQSSDTYGQQ
GSSTPGKQGSSYGQSSDTYGQQGYSYGQSSDTYEQQGSSTPGNQGSSYGQSSDTYGQQGSSTPGNQGSRYGQSSDTYGQQGSS
YGQSSDTHRQSGSGTTGEQGSIHGQSTDTYGQPGSSETGKQGSSHGQSSDTYGQSWPGHWQSSQSSGPSAGSPASPQRQGSAS
GTVSGSFYPSSSTPLYEYIQEQRGLY

>Ta_SFTP2
MSQLLKSIATIIDVFYHYTQRDGDYETLSKRELKELLKKEFHPILKHPNDPDTVDIIMQMLDQDHDRRVNFAEFLLMVFKLTQ
ACNKMVSKDFRSASGSKQKSGRHPWGQGHSSQPEGEGEEGEEDEEGEEGEEEEEDGEEEAGRGAGQSSWSAGEERGSEPSGET
VKHGHQGDPDGHKAGHGHQGGSGKQKHKLGGKYVGEKQGSKKRGSSRSRTESLTKQGSGQSGGCGGQGVSWGFGGEECPSGSN
QSGACGRQGGQSGKGQSTSYVGRGSSTGGQSGRSAGQGHGSGAGDHSDYEQQWSNSGSQTTGHGGHGSISGGQSGSSGGNRSG
SRGGATKDGQTLDSYGQQETSHGQTGSSHGQTRGTYGQTGTRQEQTGSSHGQTTDSYGQQGTSHGQSGYSHGQNTGSYGHQGT
RHGQIESSHGQITDSYGQQGTSHGQSGSSHGQTRDSYGQQGTSHGQSGSSHGQTRGNYGQQGNSRGQSGSNHGQTTDSYGQQG
TSHGQTGYTYGQTTDSYGQQGATHGQSGYSHGQNTDSYGHQGTSHGQTESSHGQTRDSYGQQGTSHGQSGSSHGQTRDSYGQQ
GTSHGQSGSSHGQTRGNYGQQGNSRGQSGSNHGQTTDSYGQQGTSHGQTGYTYGQTTDSYGQQGAIHGQSGYSHGQNTDSYGH
QGTSHGQTESSHGQTRDSYGQQGTSHGQSGSSHGQTRDSYGQQGTSHGQTGSSHGQTRGNYGQQGTSHGQSGYSHEQNTDSYG
HQGTSHGQIESSHGQITDSYGQQGTSHGQSGSSHGQTRDSYGQQGTSHGQTGSSHGQTRGNYGQQGNSHGQSGSNHGQTTDSY
GQQGTSHGQTGYTYGQTTDSYGQQGATHGQSGYSHGQNTDSYGHQGTSHGQTESSHGQTRDSYGQQGTSHGQSGSSHGQTRDS
YGQQGTSHGQSGSSHGQTRGNYGQQGNSRGQSGSNHGQTTDSYGQQGTSHGQTGYTYGQTTDSYGQQGAIHGQSGYSHGQNTD
SYGHQGTSHGQTESSHGQTRDSYGQQGTSHGQSGSSHGQTRDSYGQQGTSHGQTGSSHGQTRGNYGQQGTSHGQSGYSHEQNT
DSYGHQGTSHGQIESSHGQITDSYGQQGTSHGQSGSSHGQTRDSYDGQQGTSHGQTGSSHGQTRGNYGQQGNSHGQTGPSHGQ
TRGTYGQTGTRQGQTGSSHGQNTDSYGQQGTSHGQSGYSHGQNTDSYGHQGTSHGQSGYSHGQNTDSYGHQGTSHGQTESSHG
QVTDSYGQQGTSHGQSGSSHGQTRDSYGQQGTSHGQTGSSHGQTRGNYGQQGTSHGQSGYSHGQNTDSYGQQGTSHGQSGYSH
GQNTDSYGHQGISHGQIESSHGQVTDSYGQQGTSHGQSGSSHGQTTDSYGQQGTSHGQTGSSLGQTRGNYGQQGNSHGQSGSR
HGQTRDSSGQTGTSHGQTGSSHGQSRGTYGQTGTRQGQTGSSHGQTTDSYGQQGTSHGQSGSSHGQTRDSYGQQGTSHGQTGS
SHGQNRGNYGQQGTSHGQSGYSHGQNTDSYGHQGTSHGQTESSHGQITDSYGQQGTSHGQSGSSHGQTRDSYGQQGTSHGQTG
SSHGQTRGNYGQQGTSHGQSGYSHGQNTDSYGHQGTRHGQIESSHGQITDSYGQQGTSHGQSGSSHGQTRDSYGQQGTSHGQS
GSSHGQTRGNYGQQGNSRGQSGSNHGQTTDSYGQQGTSHGQTGYTYGQTTDSYGQQGATHGQSGYSHGQNTDSYGHQGTSHGQ
TESSHGQTRDSYGQQGTSHGQSGSSHGQTRGNYGQQGNSRGQSGSNHGQTTDSYGQQGTSHG
QTGYTYGQTTDSYGQQGAIHGQSGYSHGQNTDSYGHQGTSHGQTESSHGQTRDSYGQQGTSHGQSGSSHGQTRDSYGQQGTSH
GQTGSSHGQTRGNYGQQGTSHGQSGYSHEQNTDSYGHQGTSHGQIESSHGQITDSYGQQGTSHGQSGSSHGQTRDSYGQQGTS

HGQTGSSHGQTRGNYGQQGNSHGQSGSNHGQTTDSYGQQGTSHGQTGYTYGQTTDSYGQQGATHGQSGYSHGQNTDSYGHQGT
SHGQTESSHGQTRDSYGQQGTSHGQSGSSHGQTRDSYGQQGTSHGQSGSSHGQTRGNYGQQGNSRGQSGSNHGQTTDSYGQQG
TSHGQTGYTYGQTTDSYGQQGAIHGQSGYSHGQNTDSYGHQGTSHGQTESSHGQTRDSYGQQGTSHGQSGSSHGQTRDSYGQQ
GTSHGQTGSSHGQTRGNYGQQGTSHGQSGYSHEQNTDSYGHQGTSHGQIESSHGQITDSYGQQGTSHGQSGSSHGQTRDSYDG
QQGTSHGQTGSSHGQTRGNYGQQGNSHGQTGSSHGQTRGTYGQTGTRQGQTGSSHGQNTDSYGQQGTSHGQSGYSHGQNTDSY
GHQGTSHGQSGYSHGQNTDSYGHQGTSHGQTESSHGQVTDSYGQQGTSHGQSGSSHGQTRDSYGQQGTSHGQTGSSHGQTRGN
YGQQGTSHGQSGYSHGQNTDSYGQQGTSHGQSGYSHGQNTDSYGHQGISHGQIESSHGQVTDSYGQQGTSHGQSGSSHGQTTD
SYGQQGTSHGQTGSSLGQTRGNYGQQGNSHGQSGSRHGQTRDSSGQTGTSHGQTGSSHGQSRGTYGQTGTRQGQTGSSHGQTT
DSYGQQGTSHGQSGSSHGQTRDSYGQQGTSHGQTGSSHGQNRGNYGQQGTSHGQSGYSHGQNTDSYGHQGTSHGQTESSHGQI
TDSYGQQGTSHGQSGSSHGQTRDSYGQQGTSHGQTGSSHGQTRGNYGQQGTSHGQSGYSHGQNTDSYGQQGTSHGQSGYSHGQ
NTDSYGHLGISHGQIESSHGQVTDSYGQQGTSHGQSGSSHGQTTDSYGQQGTSHGQTGSSLGQTRGNYGQQGNSHGQSGSRHG
QTRDSYGQTGTSHGQTGSSHGQSRGTYGQTGTRQGQTGSSHGQTTDSYGQQGTSHGQSGYSHGQNTGSYGHQGTSHGQSGSSH
GQTRDSYGQQGTSHGQTGSSHGQTRGDYGQQGNSHGQTGSSHGQNADSYGQQGTSHGQSGYSHGQNTGSYGHQGTSHGQSGSR
HGQTRDSYGQQRTSHGQSGSSHGQITDSYGQQGTSHGQTGSSKGQTRGTYGQTGTRQGQTGSSHGQNTDSYGQTGTSHGQSSY
STGERTGRHGSGQSESGRSGYSATGSRDSSPGQYEGEETHYGSSYGQTGTSHGQTTDRYGQTGSSHRQTGSSVGQSGYSHGQT
RDSYGQTGASHGQTGSSHGQTGSTHGQTGHSQGQIGSSHGQTRDIYGQTGSSHRQTETSHGQTGSNQGQSGTSQGQTGSGYGQ
TGSSYGQTSYSQRQISDSWGQSGTSSVQAGGRGRQRQRSGQNWRHGSYGNLAYDYGEFGFGQSSYGVTVSRNSSPGQPGSGRT
GTQGSSYGQSSDTYGQQGSRTPGKQGSSYGQSSDTYGQQGSSYGQSSDTYGQQGSSTPGNQGSSYGQSSDTYGQQGSSTPGNQ
GSSYGQSSDTYGQQGSSTPGNQGSSYGQSSDTYGQQGSSTPGNQGSRYGQSSDTYGQQGSSYGQSSDTHRQSGSGTTGEQGSI
HGQSTDTYGQPGSSETGKQGSSHGQSSDTYGQSWPGHWQSSQSSGPSAGSPASPQRQGSASGTVSGSFYPSSSTPLYEYIQEQ
RGLY

>Ta_SFTP3
MSQLLKSISTIIDVFYHYTQRDGDYETLSKRELKELLKKEFRPILKHPNDPDTVDVIMQMLDQDHDRRVNFAEFLLMVFKLTQ
ACNKMVSKDFRSASGSKQKSGRHPWGQGHPSQPEGEGEEGEEDEEGEEGEEEEEDGEEEAGRGAGQSSWSAGEERGSEPSGET
VKHGHQGDPDGHKAGHGHQGGSGKQKHKLGGKYVGEKQGSKKRGSSRSRTESLTKQGSGQSGGCGGQGVSWGFGGEECPSGSN
QSGACGRQGGQSGKGHSTSYVGRGSSTGGQSGRSAGQGHGSAGDHSDYAQQWSNSGSQTTGHGGHGSISGGQSGSSGGNRSG
SRGGATKDGQTLDSYGQQETSHGQSGSSHGQTRGTYGQTGTRQGQTGSSHGQNTDSYGQQGTSHGQSGSAHGQNTDSYGHQGT
SHGQTESSHGQITDSYGQQGTSHGQSGSSHGQTRDSYGQQGTSHGQTGSSHGQTRGNYGQQGTSHGQSGYSHGQNTDSYGHQG
TSHGQTVSSHGQITDSYGQQGTSHGQSGSRHGQTRDSYGQTGTSHGQTGSSHGQTRGTYGQTGTRQGQTGSSHGQNTDSYGQQ
GTSHGQSGYSHGQNTDSYGQQGTSHGQSGYSHGQNTDSYGHQGTSHGQTESSHGQITDSYGQQGTSHGQPGSSHGQTRDSYGQ
QGTSHGQTGSSHGQTRGDYGQQGNSHGQSGSNHGQTTDSYGQQGTSHGQTGYTYGQTTDSYGQQGTSHGQSGYSHGQNTDSYG
HQGTSHGQTESSHGQTRDSYGHQGVSHGQSGSRHGQTRDSYGQQGTSHGQTGSSHGQTRGNYGQQGNSHGQSGYSHGQNTDSY
GHQGTSHGQTESSHGQITDSYGQQGTSHGQSGYSHGQNTDSYGHQGTSHGQTESSHGQITDSYGQQGTSHGQPGSSHGQTRDS
YGQQGTSHGQTGSSHGQTRGDYGQQGNSHGQSGSNHGQTTDSYGQQGTSHGQTGYTYGQTTDSYGQQGTSHGQSGYSHGQNTD
SYGHQGTSHGQTESSHGQTRDSYGHQGVSHGQSGSRHGQTRDSYGQQGTSHGQTGSSHGQTRGNYGQQGNSHGQSGYSHGQNT
DSYGHQGTSHGQTESSHGQITDSYGQQGTSHGQSGSSHGQTRDSYGQQGTSHGQTGSSHGQTRGNYGQQGTSHGQSGYSHGQN
TDSYGHQGTSHGQTESSHGQVTDSYGQQGTSHGQSGSSHGQQRDSYGQQGTSHGQTGSSHGQTRGDYGQQGNSHGQSGSNHGQ
NTDSYGQQGTSHGQTGYTYGQTTDSYGQQGTSHGQSGYSHGQNTDSYGQQGTSHGQSGSRHGQTRDSYGQTGTSHGQTGSSHG
QTRGTYGQTGTRQGQTGSSHGQTTDSYGQQGTRHGQSGYSHGQNTGSYGQQGTSHGQSGSSHGQTRDSYGQQGTSHGQTGSSH
GQTRGDYGQQGNSHGQSGSNHGQTTDSYGQQGTSHGQTGYTYGQTTDSYGHQGTSHGQSGSRHGQTRDSYGQTGTSHGQTGSS
HGQTRGTYGQTGTRQGQTGSSHGQNTDSYGQQGTSHGQSGYSHGQNTDSYGQQGTSHGQSGSSHGQTRDSYGQQGTSHGQTGS
SHGQTRGDYGQQGNSHGQSGSNHGQTTDSYGQQGTSHGQTGYTYGQTTDSYGHQGTSHGQSGSRHGQTRDSYGQTGTSHGQTG
SSHGQTRGTYGQTGTRQGQTGSSHGQNTDSYGQQGTSHGQSGYSHGQNTDSYGQQGTSHGQSGSSHGQTRDSYGQQGTSHGQT
GSSHGQTRGNYGQQGTSHGQSGYSHGQNTDSYGQQGTSHGQSGYSHGQNTDSYGHQGTSHGQTESSHGQITDSYGQQGASHGQ
SGSSHGQTRDSYGQQGTSHGQTGSSHGQTRDKYGQQGNSHGQSGSNHGQTTDSYGQQGTSHGQTGYTSGQTTDSYGQQGTSHS
QSGYSHGQNTDSYGQQGTSHGQTESSHGQVTDSYGQQGTSHGQSGSSHGQQRDSYEQQGTSHGQTGSSHGQTRGDYGQQGNSH
GQSGSNHGQNTDSYGQQGTSHGQTGYTYGQTMDSYGQQGTSHGQSGYSHGQNTDSYGQQGTSHGQSGSRHGRTRDSYGQTGTS
HGQTGSSHGQTRGTYGQTGTRQGQTGSSHGQTTDSYGQQGTSHGQSGYSHGQNTGSYGHQGTSHGQSGSSHGQTRDSYGQQGT
SHGQTGSSHGQTRGDYGQQGNSHGQTGSSHGQNADSYGQQGTSHGQSGYSHGQNTGSYGHQGTSHGQSGSRHGQTRDSYGQQR
TSHGQSGSSHGQITDSYGQQGTSHGQTGSRKGQTRGTYGQTGTRQGQTGSSHGQNTDSYGQTGTSHGQSSYSTGERTGRHGSG
QSESGRSGYSATGSRDSSPGQYEGEETHYGSSYGQTGTSHGQTTDRYGQTGSSHHQTGSSVGQSGYSHGQTRDSYGQTGASHG
QTGSSDGQTGSTHGQTGHSQGQIGSSHGQTRDIYGQTGSSHRQTETSHGQTGSNQGQSGTSQGQTGSGYGQTGSSYGQTSYSQ
RQISDSWGQSGTSSVQTGGRGRQRQRSGQNWRHGSYGNLAYDYGEFGFGQSSYGVTVSRNSSPGQPGSGRTGTQGSSYGQSSD
TYGQQGSSTPGKQGSSYGQSSDTYGQQGSSYGQSSDTYGQQGSSTPGNQGSSYGQSSHTYGQQGSSTPGNQGSSYGQSSDTYG
QQGSSTPGNQGSSYGQSSDTYGQQGSSTPGNQGSSYGQSSDTYGQQGSSYGQSSDTYRQSGSGTTGKQGSIHGQSTDIYGQPG
SSETGKQGSSHGQSSDTYGQSWPGHWQSSQSSGPSAGSPASPQRQGSASGTVSGSFYPSSSTPLYEYIQEQRGLY

>Ta_RPTN (XP_038624401.1)
MSQLINSILRIIEVFQNYANADDNCVSLTKTELSELLQAEFGNLLRRPRDPKTVDTILQLLDRDRDGVVGFNEFLLLVFKMAQ
ACHQALGNDACGRGRDQGPEEEEGEGKRPGEAEAQRRRTGQTEVTGRRREDTPRGQAGGQRRESSSGQSERQRSDFQDNHSES
PRWTSSYRQSERRDSRSGQSERQRSDFRDGQSESPRWTSSCGQSERRDSHSGQSERQRSDFRDGQYESPRRTSSYGQSKRRDS
RSGQSERQRSDSRDDGQSESPRRTSSYDHSERRDSRSGQSERWGQGSNSGQYESPSRTTGSGRSEKQWSDSQESQPESPRRAS
SYGQSERRDSCSGQSESRGERERQKRVVQDGGREQSGHPLGQWDPETEAQSRRKKLTSQPATSLCHQDPSWQSYESLRAHRAG
RTGQGRDEGQSHQPQDGYTDDEEQSGQAHGQRRQSRGRQVEEEDQTTQAGGGQRCQSLGRQQSPSQDWQTDEEESSRQTRDGQ
RRQSRAANMEEEDQSSQTRDGQWRQSRGGYPEDEDQSRQARDGQRRQARGGYSEEEEHSREKKRTSQSATSLCHQGPTWQSCE
FLRARRGGRTGQRRDEGQSRQPQGRYTDDEEQSRQARDGQRCQSQGGQAEEENQSVQTRQSRGRQQGLSQDRQTDDEEPIRQT
LDRQRRQARGEYTEEEEQSHQTQDGQWRQSRAANTADEGQSRQAWDGQRRQSRGGYTEEEEQSREKKRSSQPVTSLCHQGPTW
QSCEFLRGRRTGRTGQGRDEGQSRQPQDRYTEDEEQSRQAGGGQRRQSRGRQQFPSQDWQTDDEEPSRRTQDGQRCQSRPANM
EDEDQSHQAQDGQRRQSRGGYTEDEDQSRQARDGQRPQSRAADMEDEDQSPQARGGYTEEEEQSREKKRTYQPATSLCHQGPT

WQSCEFLRARREGRTGQKRDEGQSRQPQDRYTDDEDQRRQAWGGQQSQSRGGQAEKENQSVHAGGEQRRQPRDRQQNPSQDWQ
TDDEEQSRQTRDGQRRQSRGGYPKDEEKSRQAQDGQRRQARGGQAEDEDHSVQAGGGQRRRSPSELARSQAQCSCPDPRAPST
TRSTVGNSAWKETPQGRGGARGRREPKASESDSNADSHLSVPQYPLYEYVHEQSVHHYQ

>Ta_TCHH
MSPLLKSIIDISEIFNYYATCDGDDVKLNKGELRTLLQREFRDVLRRPQDPQTVDLVLQLLDRDRDGSVDFNEFLLLVFKVAQ
ACYSALSQVAGSTKREKGRDFSQERQSRQEEDQRCRGQRDGERHQQQEQDGELQRERRCEKEEEEEEEEQRQKQQEERREQLC
NEEAPRERQRQQLCEEERRQRQHERQREQRSEEEEQRERRREQLCDEEEQRERFSEQRFEEEEQFLGQRKQRCEEEEEQRLRQ
RLRRREEKRRLKQQEELCEEDRRQRQSQRDEALEEAKHRPVSWRWQVEREGQARQNKLYSQPRVPERVRRTARDQRLQGAEES
RRREKERRHTEEEEQFRRLDQDQRLREEEKDEEERSTRDLEQNQNLNWERQVEEEKAQHRNKLYSKPRSQEETRREKQERELR
EELEELRQQERDRQSRGEDHVRRQEQERKLREELEELRQQESDRQSRGEDQVRRQERERNLREELEELRQQERVRQCWGEDQV
RRQERERKLREELEELRQQERDRQSWGEDQVRRQERERKLREELEELRQQGRDRQSRGVNQFRRQERERKLREELEELRQERD
RQSRGEDQVRRQERERKLREELEELRQQERNRQSRGEDQVRRQERERKVREELEELRQQGRDSQSRGEDQVRRQERERKLREE
LEELRQQERDRQSRGEDQVRRQERERKLREELEELRQERDRQSQGEDQVRRQERERKLREELEELRQERDRQSRGEDQVRRQE
RERKLREELEELRQQERDRQSRGEDQVRRQERERKLREELEELRQQERDRQSRGEDQVRRQERERKLREELEELRQQARDSQS
RGEDQVRRQERERKLREELEELRQQERDRQSRDEDQVHRQERERKLTEELEELRQQGRDLQSRGEDQFRRQERERKLREELEE
LRQQERDRQSRGENQVRRQERERKLREELEELRQQERDRQSRGEDQVRRQERERKLREELEELRQQERDRQCWGEDQVRRQER
ERKLREELEELRQQGRDRQSQGEDQVRRQERERKLREELEELRQQERDRQSRGEDQVRRQERERKLREELEELRQQERDRQSR
GEDQVRRQERETQLREELEELRQQERDRQSRGEDQVRRQERERKLREELDELRQQGRDRQSRGEDQVRRQERERKLREELDEL
RQERDRQSRDEDQVRRQERERKLREELEELRQERDRQCWGEDQVRRQERERKLREELEELRQKGRDRQCLGEDRVRRQEEERE
LREELEELQQRETGLKRYEEDQNRPLKSQRQPERENVVRNKEVFSKAYEVDEAEQLQSQDLPSTEEGYCGCEVLSRETLEEER
RRQARDQQFYRDAQNHRESQEEERRLLWERERKFREEERRCREEEEEEVEQRRRQERERELQEEELEEEVQELQRLRRDRDRKL
REEERLRREEEEEEVEEELEELRRERNRKLREEEQLRREEEVEQELEELCWERNRKLREEEQLLQEEREEEKRRRQERERKLRE
EEEGQRFRRERDRKFREEEHLLGEEGEEEQRFRRERERKLREEEQLRKEEREEEKKRRQGRERKFQEEEQDLKRLRRERDRKL
REEEQLRKEEEEVEQELKKLRRGRDRRVREEKREQNRFQIQDRQTPDPRSRPLSNPTARSSPLYEYIQEQRSQYCP

C

>Ta_PGLYRP3
MLRRVVLLLAVGMRSSDSSPPAVTSHAPQPPTAYSNATEALARFKILLGCFRDIFQEPPKIIPRAEWGAQAPHCTVPLKTPTP
YLLFHHIAGMDCGAQGSSWCVRQLQDHHTHTNGWCDIAYNFLIMESGEVFEGTGWTVQGHHTAGYNEVALGFALLTNMTDQAP
SQAALASAQHLISFAVQKRHLSPNYIQPFLVHGEDCLQVSPGSTPSGVLGCPTIIPRADWGAMGSMANCRKLDRPTKYVIIIH
TTGQPCTESDSCKELVRGIQDFHMNGRKFCDVGYNFLVGEDGNVYEGVGWGTEGAHTYGYNDIALGVAFLGLFEEKPPNAVAL
LAAQRLIRCSVDQGYLDPNYLLVAHSDIINSISPGRATYDIIKTWPHFKG

>Ta_S100A9 (XP_038624912.1)
MEKALGDVIDVFHQYSVRVGNPDTLTKRELRQLINKELPNFLKDQQCPAEVAKILEDLDSNQDFELSFEEFVVLITRLTVASH
NKMHENAGSGPGHSHGPGLGESGHGHGHSHGPGHGHSHKH

>Ta_S100A11 (XP_038624799.1)
MAKVIVNPTETERCIESLIAVFQRYAGQEGNNCTLSKTEFLKFMNTELAAFSKSQKDPGVLDRMMKKLDLNCDGQLDFQEFLN
LIGGLAQACHASFTAAPPATHLQHKKI

Supplementary Figure S2. Amino acid sequences of proteins encoded by EDC genes of echidna. (A) Amino acid sequences of proteins encoded by SEDC genes of echidna. **(B)** Amino acid sequences of echidna SFTPs. **(C)** Amino acid sequences of proteins encoded by other EDC genes of the echidna. To show the peculiar amino acid compositions of SEDCs and SFTPs and the importance for protein cross-linking the following amino acid residues are highlighted: lysine (K) and glutamine (Q) as potential sites of transglutamination; cysteine residues (C) as potential sites of disulfide bonds; glycine (G), proline (P) and serine (S) are highly abundant residues not directly involved in cross-linking. When available, the GenBank accession number is shown behind the protein name. Only the S100A proteins whose genes are flanking *PGLYRP3* and *TCHH* are included here. SEDC, single coding exon epidermal differentiation complex; SFTP, S100 fused-type protein; Ta, *Tachyglossus aculeatus*.

A

>Md_LOR

MSYQKTQPTPQPPVGCVKVSGGGGGGGGGGSGGGCGGGSGGSGSGCGGGSSGGGGGGYCYSSGGGGGGGGGGCYSSGGGGGGG
CYSSGGGGGGGCYSSGGGGGGGCYSSGGSGGGGCYSSGGGSSGGCGGGSSGGGSSGGCGGGSSGGGSSGGGGYYQSHQKCSGS
SGGGGSGGSGGSSCGGGSGGGGGGGCGGGSGGSYYPSGGCGGGSGGGSSCGGGSGGGSSCGGGSGGGSSCGGGSGGSYYPSGG
CGGGSSGGGGGGSSSCGGGSGGSGGYYSGGSSQQCAQAPSQQSSGGSYGGGSGCGGGGYSGGGSSCGGGSYSGGGGSSCGGGG
SSGGGYSCGGGGSSGGGYSCGGGGSSGGGYSCGGGGSSGGGSGCGGGGYSGGGSGCGGGGYSGGGSSGCGG
GSGGGSSGGGSSGGGKGVPVAHQTQQKQKSCWPTK

>Md_PRR9

MSFNEHQCKQASLPPPCLQKTQEKCPVQVPDPCPPPCQEKCPVKAQEPCQEISPQQCQDPCAPQCQDPCEPQCQDPCAPQCQD
PCAPQCQDPCAPQCQDPCAPQCQDPCAPQCQDPCAPQCQDQVLEPQCQEPSQIKCKETVPQKCALPSQGK

>Md_LELP1 (XP_007481977.1)

MSSEDDKNKPCCENKCQPSCLKKLLQRCSEKCNCPPKCPPPCPPPCPKPCPFKCPPPCPPPPPCPPKCPCPPKCPPPCPPPCP
PPCPPPCPPPE

>Md_SPRR2EL

MSYQYDQQCKQPCQPPPICLKPCEPTCQDPCLTKCYESCIPKCPKPCIPKCPEPCVPNCYKPCVPKYPDPCIPKCPEPCVSKC
PESCIPNCYEPCIPKCPEPCIPNCYEPCVPKCPEPCIPKCPEPCVPNCYEPCVPKCPDPCIPKCPEPCVSKCPESCIPNCYEP
CIPKCPEPCVPNCYEPCVPKCPEPCIPKCPEPCIPKCPEPCIPKCPEPCVPNCYQPCIPKCPEAYPSPHCPKRCSPSPSPCQQ
QYPPKGK

>Md_SPRR1AL

MSYQYDQQCKQPCQTPPICLKSCESTCRTSCLPKCPESCIPKCPEPCIPKCPEPCIPKCYEPCIPKCPEPCIPKCPKPCIPKC
PEPCIPRCPEPCIPNYSKPCVPKCYEPCIPKCPEPCIPKCPEACIPKCYEPCIPKCPESCFPKCPESCSPPQCLKGCPPCPAP
CQLQYIPKGK

>Md_SPRR2L1

MSYQYDQQCKQPCQPPPNCIKSCESMCRTSCLPKCSETCVRKCYEPRVPKCYEPCVPKCYEPCIPQML

>Md_SPRR2L2

MSYQHDQQCKQPCQAPPMCTQPCQPIYWEPCVPQGPESCISKCLEPSLPKCYKPYIPNCHEPCIPKCSEPCVPKCLEPCLPKC
YKPCIPKCPEPCIPYCSEPCVPKCPEPCLPKCYEPCIAKCPEPCPLPQWKCHPCPPSCQQQCPSRWK

>Md_SPRR2L3

MSYQCDQQSKQPCQAPPMCPQPCQPICWEPCVPQCPEPCIPKCPASYILKCPKPCVPKCLDPCLPKCYKPCIPKCPEPCNPKC
PEPCISKCPEPCLPKCYVPLTAKCPEPCPLPQWKCHPCPLSCQQQCPSRWK

>Md_SPRR2L4

MSYQYDQQCKQPCQAPPKCPQPCQPICWEPCVPKCTQPCISKYPEPCVPKCYKRCIPYCPEPCIQKCPEPCIPKCPVPCLSKC
YEPCIVKCPEPCPPPQWKCHPCPPSCQQQCPPRWK

>Md_SPRR2L5

MSYQYDQQCKQPCQAPPICPKSCQPMCWEPCVPQCPEPYIPKSPASCIPKCPDPCVSKCLEPCAPKCYKPCIPHCPEPCIPKC
PELCPEPCLPKCYEPCIAKCPEPCPSPQRKCYQCPPSCQQ

>Md_SPRR2AL2

MSYQHDHQHRQPCQLPPICLQPCQHTYQDSCLPKCPEPCNPYCPEPCIPKCSDPCIPKCPETCLPKRPEPCNPYCPEPCIPKC
PETCLPKCPEPCNPYCPEPCITKCPEPCNLCCSEPCIPKYPEPCLPKCPEPCVSKCPEPCLWKCHPCLAPSQKQCSPKGL

>Md_SPRR1BL (XP_016285842.1)

MASQQQKQPCTLPPQESCASQTKEPCHPKVPEPCHPKMPEPCHPKVPEPCHPKVPEPCHPKVPEPCHPKVPEPCITTPSLAQE
KCPQVPKTKQK

>Md_SPRR1AL3 (XP_016285841.1)

MSSHQHKQPNTIPPQLHQQQVKQPSQPPPQEPCNPKGPQPGTTKLPQPNYPKVPQPGTTKLPEPCQPTVPGQHPTKKPEPYYP
VVSVPTQSKVPEPGTTKVPGSYPPPAQEKYPQVPKTKQK

>Md_SPRR1L (XP_001371801.1)

MSSHQQKQPNIIPPQLQQHQVKQPCQPPPQEPCDPKGPQPGTTKLPQPNYPKVPEPGTTKLPEPCHPIVPEPGTTKLPEPCQP
TVPGQHPTKKPEPYYPVVSVPTQPKIPEPGTTKVPGSYPPPAQEKYPQVPNTKQK

>Md_SPRR1BL (XP_007481975.2)

MSSQQQKMPCTAPPQVNQQQVKQPCQPPPQEPCQPKVPEPCHTKVPEPCHPKVPEPCHTKVPEPCHPKVPEPCHTKVPEPCNP
KVPEPCHPKVPEPCTTKVTPPPAQEKCPQVPKTKQK

>Md_SPRR4 (XP_007481974.1)
MSSQQQQQQEQCPSQQPQQQQVKQPCQPPPVKCQDPCAPSTKDPCPPKGTVVPAQKCPPAQQKCPPAQQKCPPAQQPKQK

>Md_IVL (XP_007481973.1)
MSQHQYKQPVSLPPVFSQEQCKQPLPQIPDPVPQEQVKQPTPVPPPCPVPEQVLDVQEETTIPVKIVPTLTPQLEQKGGQELE
EQLEQQLEHKQEQQLDHKQVQQLEEQLEHKQEQQLGEQREHKHEQQLDHKQVQQLEEQLEHKQEQHLGEQLDQQLEEQLEHKQ
EQQLEHKQEQQLEEQLEHKQEQHLGEKLDQQLGEQLEHKQEQQLEHKQVQQLGEQLDQPLGEQLEHKHEQQLEHKQEQHLGEQ
LDQQLGEQLEHKQEQQLGHKQVQQLEEQLEKQEEDLKQQLEQQLEEKEEEMKQQLEQQLEEKEEELKQQLEEKEEQQ

>Md_LCE1F1 (XP_007481972.1)
MSCQQSQQQCQPPPKCSTPKCPPKCPPPVSSCCGSSSGGCCGSSCGGGGGGCCLGSHRRSRRRQRQSSGCCGSSGGHSSGGGG
CCGSGHSSGGSGCCSSGHSSGGSGCCSSGHSSGGSGCCSSGHSSGGGCCSSGHGSSGSGGGCCSSGGGSSQQSGGCGGCC

>Md_LCE1F2 (XP_056670630.1)
MSCQQSQQQCQAPPKCQNPKCPPQVSAPTLAPCPPPESSCCEPSSGCCCVTGSGACCASTSGGCCNSGAGTGGGFSLFPHRPW
RSYRFWNLRSSSDCCGSGGNQPGSGGCC

>Md_KPRP (XP_016285840.2)
MCDQQQQQCCVKGSSFVPSAAGAPVNGDNIYSGNGQASGQSQICYVQCQAPSQAQGSYIEYQVGCPVQTYVQSSPACCTETCY
VQCPAPCPTEVYIPVPAPQPVQTYVEYYPQRQSQGYYNSCAPQRQSQGYYSSCAPQRQSQGYYSSCVSQRQSQGYYSSCAPRR
QSQYYDQYQGSYSGCSPQNQCRQSYSRSQAPCMSRPSYRSYSPPCGPPRVSQPAYRSCSPPCGPPRMSRPTYRSCSPPCGPPR
MSRPTYRSCSPPCGPPRMSRPTYRSCSPPCGPPRMSRPTYRSCSPPRMSRPTYRSCSPPRMSRPTYRSCSPPYSPPRMSRPTY
RSCSPPHRSWPTCTYCTPPRQSQPCYDSCLPRRPASGSSQRCGPKYRVEISSSNCPAQVPPIRRPVQIPPCRRPCQGSSTQYS
CSAPCPRPCPEPYPRRGSHSSSEQGPRPCPLPAPRSCRKPCPEPCPAPRPEPCPAPRPEPCPAPQPSLEPYPEPCCPEPPNME
SCGRSSPCNYSKPCPSPPPCPSFSGCNESDPRHLDIEPPLCGPAGYNEGGACGSGGCSGPQDMGGSGTKGGAFCGVKGCYF

>Md_LCE3L1
MSSQQNQQPYGPPPKGQTPKDLPKCPPQTPAPAFSCCSSNTGGGYKSSSGGPYSPFSQWRRKSIRRRVHSSDYRGRGNGQSQK
STECGGYGGSNGGFSWLSPQEQH

>Md_KPLCE (XP_016285838.1)
MCDQQKQPQFLPCCVKGSALGSIETAQGPASSQVDVSCEYGPEPCQSKSSFIKSTSSFFQKGQSKCAPVKCPPPCQTTYVKCP
PPCQTTYVKCAPPCQTTFVKCPAPCQTTFVKCPPPCQTTYVTCPPPCQTTFVKCSPPPCQTTCVKCPAPCQTTSVKCPAPCQT
TSVKCPAPCQTTCVKCPAPCQTTSVQCQAPCQVQTPCQTQTCYVQSASPCQTQTYYVQTPSVQYLAPSSYYTSSSYGTTCCPA
APMSFGVRPLRRWIRGPQCRENTGCCEDSGCCSSCCCSSGCCEGGCCCLGILPMRSRGPVCCANDDDCCC

>Md_LCE5AL1 (XP_007485557.1)
MSCQQSQQQCQAPKCPPKCPPKCQTPKCPPKCPPKCPPKCPPKCPPQAPCPPPSCCGSSSGGCCSSGGCCGSSSGGCCSSGSG
GGCCLFSHHRRSHRRRHQRSDCCDSGSGRSQQSGGCCGSSGGCCGSSGGCC

>Md_LCE1EL1 (XP_007485556.1)
MSCQQSQQQCQAPKCPPKCPPKCQTPKCPPKCPPKCPPKCPPKCPPQAPCPPPSCCGSSSGGCCSSGGCCGSSGGCCSSGSGG
GCCLFSHHHRRSHRHRRQKSDCCDSGSGHSQQSGGCCGSSGGCCGSSGGCCGSSGGCC

>Md_LCE1EL2 (XP_007485555.1)
MSCQQSQQQCQAPKCPPKCPPKCQTPKCPPKCQTPKCPPKCPPKCPPQAPCPPPSCCGSSSGGCCSSGGCCGSSGGCCSSGSG
GGCCLFSHHHRRSHRHRRPKSDCCDSGSGRSQQSGGCCGSSGGCCGSSGGCCGSSGGCC

>Md_LCE1EL3 (XP_056670844.1)
MSCQQSQQQCQAPKCPPKCPPKCQTPKCPPKCPPKCPPKCPPKCPPQAPCPPPSCCGSSSGGCCSSGGCCGSSGGCCSSGSGG
GCCLFSHHHRRSHRHRRPKSDCCDSGSGRSQQSGGCCGSSGGCCGSSGGCCGGSGDCC

>Md_LCE2AL1 (XP_007485554.2)
MSCQQSQQQCQAPKCPPKCPPKCQTPKCPPKCPPKCPPKCPPKCPPQAPCPPPSCCGSSSGGCCSSGGCCGSSSGGCCSSGSG
GGCCLFSHHHRRSHRHRRPKSDCCDSGSGRSQQSGGCCGSSGGCCGSSGGCCGSSGGCCGSSGGCC

>Md_LCE2DL (XP_016285194.1)
MSCQQSQQQCQAPKCPPKCPPKCQTPKCPPKCPPKCPPKCPPQAPCPPPVSSCCGSSSGGCCSSGGCCGSSSGGCCSSGSGGG
CCLFSHHRRSHRRRHQRSDCCDSGSGRSQQSGGCCGSSGGCCGSSGGCCGGSGGCC

>Md_LCE2CL (XP_016285193.2)
MSCQQSQQQCQAPKCPPKCPPKCQTPKCPPKCPPKCPPKCPPQAPCPPPVSSCCGSSSGGCCSSGGCCGSSSGGCCSSGSGGD
CCLFSHHRRSHRRRHQRSDCCDSGSGRSQQSGGCCGSSGGCCGSSGGCCGGSGGCC

>Md_LCE5AL3 (XP_016284707.2)
MSCQQSQQQCQAPKCPPKGPPKCQTPKCPPKCPPKCPPQAPCPPPVSSCCGSSSGGCCSSGGCCGSSSGGCCSSGSGGGCCLF
SHHRRSHRRRHQRSDCCDSGSGRSQQSGGCCGSSGGCCGSSGGCC

>Md_LCE1EL4 (XP_016284705.2)
MSCQQSQQQCQAPKCPPKCPPKCQTPKCPPKCPPKCPTQAPCPPPVSSCCGSSSGGCCSSGGCCGSSSGGCCSSGSGGGCCLF

SHHRRSHRRRHQRSDCCDSGSGRSQQSGGCCGSSGGSCGSSGGCC

>Md_LCE2AL2 (XP_007485553.1)
MSCQQNQQQCQPPPKCQAPKCPPKCPPQAPCPAPASSCCGSSSGGCCGSSSGGCHSSSSGGGCCLFSHHHRRSHRHRHQRSNC
CDSGSGHSQQSGDCCGNSGGCC

>Md_LCE3L2
MSCQQNQQQCQPPPKCQTPKCPPKSPQAPCTPPVSSCGASSSVGCHSSGSGAGCGSGSEGGCCLFSHHHRRSHRRNRQHSDCC
GSGSGHSQQSGDSCGNSGGCC

>Md_LCE3L3
MSCQQNQQQCQPPPKCQTPKCPPKSPQAPCTPPVSSCGASSSVGCHSSGSGAGCGSGSEGGCCLFSHHHRRSHRRNRQHSDCC
GSGSGHSQQSGDSCGNSGGCC

>Md_LCE3L4
MSCQQNQQQCLPPPKCQAPKCPPQAPCSPPISSCSGSSFGGGCSSGSEIGCSSGSEGGCSFFPHHNRRSHRHGSQGSDCFDSG
SGHSQQFEDSCESSGNCC

>Md_LCE3L5
MSCQQNQQQCQPPPKCQTPKCPPKSPQAPCTPPVSSCGASSSVGCHSSGSGAGCGSGSESGCCLFSHHHRRSHRRNRQHSDCC
GSGSGHSQQSGDSCGNSGGCC

>Md_LCE3L6
MSCQQNQQQCLPPPKCQAPKCPPQAPCSPPISSCSGSSFGGGCSSGSGIGCSSGSEGGCRLFPHHHRRSHRHGHQGSDCFDNG
SGHSQQFGDSCESSGNCC

>Md_LCE3L7
MSCQQNQQQCQPPPKCQTPKCPPKSPQAPCTPPVSSCGASSSVGCHSSGSGAGCGSGSEGGCCLFSHHHRRSHRRNRQHSDCC
GSGSGHSQQSGDSCGNSGGCC

>Md_LCE3CL (XP_007485552.2)
MSCQQNQQQCQPPPKCQTPKILPQAPCSPPISSCSGSSSGTCSGSSSGRGCSSSSEDGCCLFSHHHRRSHRCRRQSSDHCDGG
SGHSQQSGGSCGSSGGCC

B

>Md_CRNN (XP_007485551.1)
MPQLLGNIDGIIQAFSRYAKTEGDCTTLTKGELKKLLEQELADVIVKPHDPATVDQVFHLLDEDSKGTVDFKEFLVLVFKVAQ
ACYKTLNESSQGACGTQKPGSQHSGSTQQRNDGDQRSHTEIGRVEREQSHTRHGGEEQTSTGQNRSNLTTGTQTQGQDVTSTQ
ICDQDRWPKTQGQERGTQQVRDKIEGQRQPETGTTQTREQGRTHQTSETVTGGQSQIHTGTTQSREQGWTHQKTDKVIGSQSQ
VHTGTTQTREQDGSHHQTRDRVTGGQSQIHTGTTQTREQDGSHHQTRDRVTGGQSQIHTGTTQTVEQDRSRHQTRDRVTGGQS
QIHTGTTQTREQGRTHQTSETVTGGQSQIHTGTTQSREQGWTHQKTDKVIGSQSQVHTGTTQTREQDGSHHQTRDRVTGGQSQ
IHTGTTQTREQDRSHHQTRDRVTGGQSQIHTGTTQTVEQDRSHHQTRDRVTGGQSQIHTGTTQTVEQDRSHHQTRDRVTGGQS
QIHTGTTQTVEQDRSHHQTRDRVTGGQSQIHTGTTQTVEQDRSHHQTRDRVTGGQSQIHTGTTQTVEQDGSHHQTRDRVTGGQ
SQIHTGTTQTVEQDGSHHQTRDRVTGGQSQIHTDTTQTVEQDGSHHQTRDRVTGGQSQIHTDTTQTVEQDGSHHQTRDRVPGG
QTQIHTGTTQVRGQGRTHQTSVTVTRGQSQTGITQTTNNQTQQLQTGYGQTRRPETQGQGQTHTQIGGGTQTQTGCSQGWGQE
RSQQTREDMVEGHGHTQVGSTLPGGQDWSDSQRSCSVTGGLQENDYCQTQEPSFGGQEWSRHQTRETVAQQQDHSRESEIHCQ
GSTQLEGAGQIQGGKRGLTARGLYSYFKGNKQ

>Md_FLG
MSHLLSSILSIIEVYYKYTSQDQDCNTLCKRELKKLLENEFRPILKNPDDPDTVEIFMQMLDRDHDKKVDFIEFLLMIFKLTM
ACNQSIGKEYCQASGSQKQHPHHHRQEQSKTKEEEEEEETDSSNSSWSAGEEPRSHTGRSKKIRHRSKSNSRGHGKLDRSSSS
GYKDIFGRKQHESKPRHSKRSENMENRSSSSDLEKMRPKSSISPSRIYGGEKHEFSSDWSGSSGRKIRVYSSEGLSYGIKSMG
YGSNSTQSKECGEQRQRCKSPQTRNCRRDHHEHACAQSDKCGKQRKRSSSCQSDESCREQAYSKMCSENYEGNVTNSCCQSQN
SRGKGHWSCSSQSASYGQGSDSGECSISGQSRSDQCQCPSSGQQRSASSRGRDVPSRRQQQDSSRQSGSSQRQSSDRRSTSRR
RSSRSQQDDISRQSGSGQRESSDSRSRGRGESSRRQQQDAPRGSGTSDRQARDASSRGRDITSRRQQQDSSRRSGSGQRQSSD
SRRSRTRGSSSRQQPESSTHSTSGQRQSSDSRSRSRGGSHRRQQNDSSRQSGSGQRETRDASSRGRGVPSRRQQQDSSRQSGS
SQRQSSDRRSTSRRRSSRSQQDDISRQSGSGQRESSDSRSRGRGESSRRQQQDAPRGSSTSDRQARDASSRGRDTTSRREQQD
SSRRSGSGQRQSSDSRRSSIQGSSSRQQPESSTHSTFGQRQSSDSRSRSRGGSRRRQQHDSSRQSGSGQRETSDSRSRDRAES
GRIQEQDSCSQSGYGQRQSSDSRSRGRGEADRRQQPDSSRQSVSSQRQTTDASSRGRDVPRRRQQQDSSRQSGSSQRQSSDRR
STSRRRSSRSQQDDISRQSGSGQRESSDSRSRGRGESSRRQQQDAPRGSSTSDRQARDASSRGRDITSRRQQQDSSRRSGSGQ
RQSSDSRRSRTQGSSSRQQPESSTHSTSGQRQSSDSRSRSRGGSRRRQQNDSSRQSGSDQRETRDASSRGRGVPSRRQQQDSS
RQSGSSQRQSSDRRSTSRRRSSRSQQDDISRQSGSGQRESSDSRSRGRGESSRRQQQDAPRGSSTSDRQARDASSRGRDITSR
REQQDSSRWSGSGQRQSSDSRRSSIQGSSSRQQPESSTHSTFGQRQSSDSRSRSRGGSRRRQQHDSSRQSGSGQRETSDSRSR
DRAESGRIQEQDSCSQSGYGQRQSSDSRSRGRGEADRRQQPDSSRQSGSSQRQTRDASSRGRDVPSRRQQQDSSRQSGSSQRQ
SSDRRSTSRRRSSRSQQDDISRQSGSGQRESSDSRSRGRGESSRRQQQDAPRGSSTSDRQARDASSRGRDITSRRQQQDSSRR
SGSGQRQSSDSRRSRTQGSSSRQQPESSTHSTSGQRQSSDSRSRSRGGSRRRQQNDSSRQSGSGERETRDASSRGRGVPSRRQ
QQDSSRQSGSSQRQSSDRRSTSRRRSSRSQQDDTSRQSGSGQRESSDSRSRGRGESSRRQQQDAPRGSSTSDRQARDASSRGR

DITSRREQQDSSRWSGSGQRQSSDSRRSSIQGSSSRQQPESSTHSTFGQRQSSDFRSRSRGGSRRRQQHDSSRQSGSGQRETS
DSRSRDRAESGRIQEQDSCSQSGYGQRQSSDSRSRGRGEAGRRQQPDSSRQSGSSQRQTRDASSRGRDVPSRRQQQDSSRQSG
SSQRQSSDRRSTSRRRSSRSQQDDISRQSGSGQRESSDSRSRGRGESSRRQQQDAPRGSSTSDRQARDASSRGRDITSRRQQQ
DSSRRSGSCQRQSSDSRRSSIQGSSSRQQPESSTHSTFGQRQSSDSRSRSRGGSRRRQQHDSSRQSGSGQRETSDSRSRDRAE
SGRIQEQDSCSQSGYGQRQSSDSLSRGKGEAGRRQEYESFRQSDCDSRESIGFISLGIEASGHGENQYSSGHSGYPIGQSNHL
IRSSQASDSPGAFVAPHRHPVGRAQQKQESGRHWVHGSYGSAEYNYGQTGFRHSGFSRTSIHNSSSMGSEHVSLSHLSLLGHS
CLRNDYSDSDMSTGFQEVFDESIEQSGEISPYSGLSTPEMSRDRFSRILGSQESSHQQAEEVPSQVKNESKSNNKQSGKKRNT
PTKSTCSQSRSRKNGIHGPICDQSSDRNSQSGSSHQITYEHTYNKNKSNSNQSFDSFDQPGTREPRHNNPSSTGRKRSVSSSS
VPGDSGNCKQDIVGSQRLEVFHIRERTNSGSYYPSSITPLYEYMQEQRCNYY

>Md_FLG2 (XP_016285131.1)
MSVLLQSIVNIIDIFYQYAEDDGECETLSKKEMKELLEKELSSIMSNPKDPQIIEAIFHILDQDHDDKVNFAEFLLMVFKLAM
SFNKSVSKEYCEASGSKHKSQDHHHKEEKSVTEEEEDETGSDTDAEEQQCSFGHSQKKGKYRNQSRSNSPWKRRRSSSSNSEH
KKDSRKRHHKSRKNEKEKNGLSSKKNGRRYKSSTHEKHGSTGRGRELSDGQTSDSEGQTRDQSRHKVSEQGQSSFTHSGSSSR
GRKESSCGQSSGSERQSKHRESSSTKRNSDHTRGRQKSNCNQESDSEGQIIDHSRESVSVHEKNKSSDSGAISSHRERCSCGQ
PSNSESQTEDSSGESRLGEQKTSSKEKKKGNQRSRQGQAEDTSAHLGSGKGKSKDRSKTRRQTSSHHSSSENKGQSTERSSHS
GSGHQQSSSSQGHRGSSQRGRQQSTSSQVSDSEGSTREHTRESVSGHRQSSSPSSGSSTSRRQGSSHGSSSHRQRQQSDRSSH
SGSGHQQSSSSQRQHGSSQRGRQQSTSSQVSDSEGSTREHTRESVSGHRQSSTPSSGSRSSRRQGSSHGSSSHRQGQQSDRSS
HSGSGHQQSSSSQRQHGSSQRGRQQSTSSQVSDREGSTREHTRESVSGHRQSSSPSSGSSSSRRQGSSHGSSSHRQRTTIRQI
QPLRIWSSTILIISETT

>Md_HRNRL1 (XP_016284680.1)
MSQLLRSIVTVIDVFYNYCGQDEECDTMCKKELKELLYKEMGPLLKNPNNPDTVDIFMQILDRDHDRRVDFTEYLLMVFKLTM
ACNKSVGKEYCHASGSKRKQQGRHHQEKQSETEDQEELKGHESGSSHSSWSSGEEYGSSRSGRSQGSKQHGHKYSSTGWRKKD
YSGNGEGSGKNYHRSSSSYSRSSSQEKHGFKDNEQWGRRRNSSISPSRKSGGEEYESGTDQSGRWRRQRHGSSNSQTGGFGVQ
EEGSKRGRGREQGHWSRSHQSSSYERPESGANQSSTYEYGQHGSSSEQYSNYGQNESGSGQSSSHGKCECGSGQSSNQSSSFG
SGSGQSSRERRHGSSSSGQSGSWGGQGYSSGSNQSSRHRQHGSGTGQSTRSGQQGSGSGQSSSYGQHGSGSSGHSGSCEGQGH
YSSSHQSSSYGNHESGSGQPSSYGQHGSGSGQSSSYGQHGSGSGQSSGYGQHGSGSGQSSEYGQHGSGSGQSSSHGQYGSGSG
QSSRYGQHGSGSGQSSSHGQYGSGSGQSSRYGQHGSGSGQSSGYGQHGSGSGQSSSYGHHGSGSSGHSERCGGQEQYSSSQKS
SSHGYHGSGSGQSSNYGQHGSGSGQSSGYGQRGSGSSQSSGYGQHGSGSGQSSSYGQHGSGSGQSSSYGQHGSGSGQSSSYGQ
HGSGSGQSSGYGQHGSGSGQSSIYGQHGSGSSGNSESCGGQEHYSSSQKSSSHGYHGSGSGQSSGYGQHGSGSGQSSSYGQHG
SGSGQSSGYGQHGSGSGQSSGYGQHGSGSGQSSSYGQHGSGSSQSSGYGQHGSGSGQSSGYGQHGSGSGQSSSYGQHGSGSSG
HSECCGGSEHYSSSQKSSSHGHHGSGSGQSSGYGQRGSGSSQSSGYGQHGSGSGQSSSYGQHGSGSGQSSSYGQHGSGSGQSS
SYGQHGSGSGQSSGYGQHASGSSQSSGYGQHGSGSGQSSSYGQHGSGSGQSSSYGQHGSGSSGHSECCGGSEHYSSSQQSSSH
GHHGSGSGQSSGYGQHGSGSGQSSGYGQHGSNSGQSSSYGQHGSGSGESSGYGQHGSGSGQSSSHSQHGSGSGQSSSYGEHGS
GSGQSSGYGQHGSGSGQSSSYGQSSSHSQHGSGSGQSSSCGQHGSGSGQSSAYGQHGSGSGQSSSYGQSSSHGQHGSGSGQSS
SYGQHGSGSGQSSSHSQHGSGSGQSPSYGQHGSSSGQSSGYGQHGSSSGQSSSHSQHGSGSGQSSGYDQHGSNSGQSSSYGQH
GSGSGESSGYGQHGSGSGQSSSHSQHGSGSGQSSSYGEHGSGSGQSSGYGQHGSGSGQSSSYGQSSSHSQHGSGSGQSSSCGQ
HGSGSGQSSAYGQHGSGSGQSSSYGQSSSHGQHGSGSGQSSSYGQHGSSSGQSSSHSQHGSGSGQSSGYGQHGSGSGQSSSHG
QHGSGSGQSSSHSQHGSGSSQSSGYEQHGSGSGQSSGYGQHGSGSGQSSSHSQHGSGSGQSSGYGQHGSGSGQSSSHGQHGSG
SGQSSSHSQHGSGSGQSSGYGQHGSGSGQSSSHGQHGSGSGQSSSHSQHGSGSGQSSSYGQHGSSSGQSSGYGQHGSSSSQSS
SHSQHGSGSGQSSSHGQHGSCSGQSSSYGQCGSSSGGQSESCGPQRHSSYGQCGSSQGQSSSYGQHGSNYGQSSRCGSGSSQS
SSQRRCRSSSGSRSESCGGRRYSSTSRRSSSYGRCGSSPCRSSSYGQQRSRYGQSSSCGSGSSQSSSQRRYRYSSGGCQGRSS
SSHRSSSYGRHGSCSSQSPSFGQHGSYSGQSSSQRQYGSCSGGRSQSCGRQGRSSSSHQSSSNRRCRSGSGQSSSYEQYGSGY
GQSSSCGSGSGQSCSQDQYGSSSGGQSMNCGQQGHSSSSYQSSSYGQCGSGCDQSYSQYGCQSSSSGPQGSGAAQYCSSGSCE
SCVTEHTPDWGRHKFSSNSICGEFGKQTVGSTFIQYEDINNESQVDRNHENKEVCSLYSQGVDDSLKVRGNYGRVRTHSGSYY
PCSITPLYEYCQEQRFYHYK

>Md_HRNRL2
MSQLLTRIATIIDVFNQYCGQDKECDMISQQELKEFLENELQFIVQNSEDSEAVDVIMLNMDLDQSGRGHLTDLVFQIIKKIL
LMKKCNKKESSQGNSYHMEEIEKEEKRWRKDLMRSNWNQKEEYGPRRSKYSLETEKVRYRLHGEEERKDSVSSSDEEKDSEKK
NYTSNSSKAGNNKGDEKTVSRKQGWRIKKESERPSKKSGGEKRETDYERSSKQEGERKKQDMKSTVGNNESNKRGQTVRFESE
EKQRTCQEKTNIYRRNYAMGKNENCDIETNDSNKFSESKQIGSGSGKSYNSKRHGTTSGKESETCETKKGSSSGHQSSKSSQY
SFPYINGTYSDSGSDSECGTCSDYRNDTGTSKSSSSNQYGSQSGHSSSTKGHKSSSGKKASSMGGRGYNSSSPSAQTHSSGPG
SGSGSDSGSGTDSDSGSGSESCSCSGSESGSGHSSGSKRQGSRSGYSSRSGRHGSSSSQSSHRGHRSSSGEESVSSGRKGSSS
GSSHRSSSNTQYGSQSGRSSSSKGHGSSSGKQSGSSGAKGGSSSSHQSSSSGQYGSGSKSSSSGSGSCSGSGSGSGSGSRSCS
SSDTSSSSSQYGSRSGRSSSSKGHGSSSGKHSGSSGAKGGSSSSHQSSSSGQYGSGPKSSGSGSGSCSGSGSGSGSNKSSSSG
QYGSGSGHSSGSKGHGSSSGGKSSSSGGRGHSSSSSSSQRHRSGSASGSGSGSESCSCSGSESGSGHSSGSKGQGSRSGHSSR
SGRHGSSSSQSSHRGHRSSSGEESVSSGRKGSSSGSSHQSSSSSQYGSRSGRSSSSKGHGSSSGKQSGSSGAKGGSSSSHQSS
SSGQYGSGSKSSGSGSGSCSGSGSGSGSGSGSGSNKSSSSGQYGSGSGHSSGSKGHGSSSGKQSGSSGAKGGSSSSHQSSSSG
QYGSGSKSSGSGSGSCSGSGSGSGSGSNKSSSSGQYGSGSGHSSGSKGHGSSSGGKSSSSGGRGHSSSSSSSQRHRSGSASGS
GSGSESCSCSGSESGSGHSSGSKGQGSRSGHSSRSGRHGSSSSQSSHRGHRSSSGEESVSSGRKGSSSGSSHQSSSSSQYGSR
SGRSSSSKGHGSSSGKQSGSSGAKGGSSSSHQSSSSGQYGSGSKSSGSGSGSCSGSGSGSGSGSGSNKSSSSGQYSSGSGHSS
GSKGHGSSSGGKSSSSGGRGHSSSSSSSQRHRSGSASASGSGSGSESCSCSGSESGSGHSSGSKGQGSRSGHSSRSGRHGSSS
SQSSHRGHRSSSGEESVSSGRKGSSSGSSHQSSSSSQYGSRSGRSSSSKGHGSSSGKHSGSSGAKGGSSSSHQSSSSGQYGSG
SKSSGSGSGSCSGSGSGSGSGSNKSSSSGQYGSGSGHSSGSKGHGSSSGKQSGSSGAKGDSSSSHQSSSSGQYGSGSKSSGSG
SGSCSGSGSGSGSGSGSGSGSNKSSSSGQYGSGSGHSSGSKGHGSSSGKQSGSSGAKGGSSSSHQSSSSGQYGSGSKSSGSGSGS
CSGSGSGSGSNKSSSSGQYGSGSGHSSGSKGHGSSSGGKSSSSGGRGHSSSSSSSQRHRSGSASGSGSGSESCSCSGSESGSG
HSSGSKGQGSRSGHSSRSGRHGSSSSQSSQRGHRSSSGEESVSSGRKGSSSGSSHQSSSSSQYGSRSGRSSSSKGHGSSSGKQ

SGSSGAKGGSSSSHQSSSSGQYGSGSKSSGSGSGSCSGSGSGSGSGSNKSSSSGQYGSGSGHSSGSKGHGSSSGGKSSSSG
GRGHSSSSSSSQRHRSGSASGSGSGSESCSCSGSESGSGHSSGSKGQGSRSGHSSRSGRHGSSSSQSSQRGHRSSSGEESVSS
GRKGSSSGSSHQSSSSSQYGSRSGRSSSSKGHGSSSGKQSGSSGAKGGSSSSHQSSSSGQYGSGSKSSGSGSGSCSGSGSGSG
SGSNKSSSSGQYGSGSGHSSGSKGHGSSSGGKSSSSGGRGHSSSSSSSQRHRSGSASGSGSGSESCSCSGSESGSGHSSGSKG
QGSRSGHSSRSGRHGSSSSQSSHRGHRSSSGEESVSSGRKGSSSGSSHQSSSSSQYGSRSGRSSSSKGHGSSSGKQSGSSGAK
GGSSSSHQSSSSGQYGSGSKSSGSGSGSCSGSGSGSGSGSGSGSNKSSSSGQYGSGSGHSSGSKGHGSSSGKQSGSSGAKGGS
SSSHQSSSSGQYGSGSKSSGSGSGSCSGSGSGSGSNKSSSSGQYGSGSGHSSGSKGHGSSSGGKSSSSGGRGHSSSSSSSQRH
RSGSASGSGSGSESCSCSGSESGSGHSSGSKGQGSRSGHSSRSGRHGSSSSQSSHRGHRSSSGEESVSSGRKGSSSGSSHQSS
SSSQYGSRSGRSSSSKGHGSSSGKQSGSSGAKGGSSSSHQSSSSGQYGSGSKSSGSGSGSCSGSGSGSGSGSGSGSNKSSS
SGQYGSGSGHSSGSKGHGSSSGKQSGRSGAKGGSSSSHQSSSSGQYGSGSKSSGSGSGSCSGSGSGSGSGSNKSSSSGQYGSG
SGHSSGSKGHGSSSGGKSSSSGGRGHSSSSSSSQRHRSGSASGSGSGSESCSCSGSESGSGHSSGSKGQGSRSGHSSRSGRHG
SSSSQSSHRGHRSSSGEESVSSGRKGSSSGSSHQSSSSSQYGSRSGRSSSSKGHGSSSGKQSGSSGAKGGSSSSHQSSSSGQY
GSGSKSSGSGSGSCSGSGSGSGSGSNKSSSSGQYGSGSGHSSGSKGHGSSSGGKSSSSGGRGHSSSSSSSQRHRSGSASASGS
GSGSESCSCSGSESGSGHSSGSKGQGSRSGHSSRSGRHGSSSSQSSQRGHRSSSGEESVSSGRKGSSSGSSHQSSSSSQYGSR
SGRSSSSKGHGSSSGKQSGSSGAKGGSSSSHQSSSSGQYGSGSKSSGSGSGSCSGSGSGSGSGSNKSSSSGQYGSGSGHSSGS
KGHGSSSGKQSGSSGAKGDSSSSHQSSSSGQYGSGSKSSGSGSGSCSGSISSSGSGSNKSSSSGQYGSGSGHSSGSKGHGSSS
GGKSSSSGGRGHSSSSSSSQRHRSGSASGSGSGSESCSCSGSESGSGHSSGSKGQGSRSGHSSRSGRHGSSSSQSSQRGHRSS
SGEESVSSGQKGSSSGSSHQSSSSSQYGSRSGRSSSSKGHGSSSGKQSGSSGAKGGSSSSHQSSSSGQYGSGSKSSGSGSGSC
SGSGSGSGSGSNKSSSSGQYGSGSGHSSSSKGHGSSSVGKSSSMGGRGYNSSSSSSQRHRSGSASGSGSGSESCSCSGSESGS
GHSSGSKRQGSRSGYSSRSGRHGSSSSQSSHRGHRSSSGEESVSSGRKGSSSGSSHQSSSSSQYGSRSGRSSSSKGHGSSSGK
QSGSSGAKGGSSSSHQSSSSGQYGSGSKSSGSGSGSCSGSGSGSGSGSGSNKSSSSGQYGSGSGHSSGSKGHGSSSGGKSSSS
GGRGHSSSSSSSQRHSSGSGCGSFSGSGSESE

>Md_HRNRL3 (XP_007485533.1)
MSQLLTSITTILDVFYQYCGEDEECDTMSQSELKKFLENELRFILKNPEDPDTVDVIMLNMDQDRDKRVDFPEFLLLIFKITM
SFNKALKRKYMQSKNNGEDENESEEEQEEEEEGKGWDTDSSNSTWNSEKEYGTFRTGQSKKKQKVRHRESVHRREDSQGLGKR
KSSEKKHHRTESSNTGRNDEDEYRIIPRKSKGKRHNSEKSPSKRLGREESETDSEKSTQKSGKRYKSNKAPKKGFNTEENECT
KKCHFVTFERQKGIYDFSGSSRKGKDSALPESGSESDSGSGKQGSSSGQSSSQRRQGSSSRRKSDSSGGKGHSSGSQQSSSSG
KDGSGSGRSSSSHGHGSSSGGKSSSSGGRGHSSSSQQSTSLGQHGSHSGYGSDSGSASGSGSGSGSDSGSEYVSDHGSGQSSG
SRQGGSRSGHSSSSGKHGSKSGQSSSHRRQGSSSGRKSDSSGGKGHSSGSQQSSSSGKDGAGSGRSSSSHGHGSSSGGKSSSS
GGRGHSSSSQQSTSLGQHGSHSGYGSDSGSGSGSGSGSDSGSEYGSDYGSGQSSGSRQGGSRSGHSSSSGKHGSKSGQSSSHR
RQGSSSGRKSDSSGGKGHSSGSQQSSSSGKDGSGSGQSSSSHGHGTSSGGKSSSSKGQGQKSTSEWSSSFASVRSSSIGRTKS
PSRTKSGSSGSKGGSSSSHQSSSSVKDRSGSGRSSSSHGHGSSSSGKSSSSRGRGHSSSSQQSTRLGQHGSHSGYGSDSGSAS
ASASGSGSESDFDSDSEYVSDHGSGQSSGSRQRGSRSGHSSSSGKHGSKSGQSSSHRGQGSSSGRKSDSSGGKGHSSGSQQSS
SSGKDGSGSGRSSGSHGHGSSSGGKSSSSGGRGHSSSSQQSTSLGQHGSHSGYGSDSGSGSGSGSESGSEYVSDHGSGQSSGS
RQGGSRSGHSSSSGKHGSKSGQSSSHRGQGSSSGRKSDSSGGKGHSSGSQQSSSSGKDGAGSGRSSSSHGHGSSSGGKSSSSG
GRGHSSSSQQSTSLGQHGSHSGYGSDSGSASGSGSGSGSESGSEYVSDHGSGQSSGSRQGGSRSGHSSSSGKHGSKSGQSSSH
RGQGSSSGRKSDSSGGKGHSSGSQQSSSSGKDGAGSGRSSSSHGHGSSSGGKSSSSGGRGHSSSSQQSTSLGQHGSHSGYGSD
SGSGSGSGSGSDSGSEYGSDHGSGQSSGSRQGGSRSGHSSSSGKHGSKSGQSSSHRRQGSSSGRKSDSSGGKGHSSGSQQSSS
SGKDGSGSGRSSSSHGHGTSSGGKSSSSKGQGQKSTSEWSSSFASVRSSSIGRTKSPSRTKSGSSGSKGGSSSSHQSSSSVKD
RSGSGRSSSSHGHGSSSSGKSSSSRGRGHSSSSQQSTRLGQHGSHSGYGSDSGSASASASGSGSGSGSDFDSDSEYVSDHGSG
QSSGSRQRGSRSGHSSSSGKHGSKSGQSSSHRRQGSSSGRKSDSSGGKGHSSGSQQSSSSGKDGSGSGRSSSSHGHGTSSGGK
SSSSRGRGHRSSSQQSTSLGQHGSHSGYGSDSGSGSGSGSGSDSGSEYVSDHGSGQSSGSRQGDSRSRHSSSSGKHGSKSGQS
SSHRRQGSSSGRKSDIYGGKGHSPGSQLSSSSGKDGSGSGRSSSSHGHETSSGGKSSSSGGRGHSSSSQQSSSLGQHGSHSGY
GSDSGSGFGSGSGSDFDSDSEYVSDHGSGQSSGSRQRGSRSGHSSSSGKQGSKSGQSSSHRGQGSSSGRKSDSSGGKGHSSGS
QKSSMSGKSIVFF

>Md_HRNR (XP_007485531.1)
MSQLLTSMATIMDIFYTYCGKDEECDTMNQSELKEFLEKELQFILKNPDDPETIDIMMLNMDLDHNKKIDFPEFLLLVFKLIM
ILNKTFKKEYCQSHTEEQSEKEEEEESDSSKSSRNSKQGRKKQNSRTRHSRRHRGKEYESDSENSSQHRGKKCRSKKNQEDDL
KKQKSSESDSDHSPSSKQQGSRTSHSSSSGRHSSGSSQSSKSGKHESGSGQSSSSKGHGSSSSGKSNSSGGRGHSSSSHQSSS
SGSLGSGSGSGSDSGSGSESDSQCGSDHSSGSKQQRSRSGHSSSSGKHGSSSGQSSSQRRHRTQSGEESESSGGKRSSSASHK
SSSSGKCGSGSGSSQSSKSGKHESGSGHSSSSKGHGSSSGGKSSSSGGRGHSSSSSSSQRHRSGSASGSGSSSGSESCSCSGS
ESGSGHSSGSKQQGSRSGHSSRSGRHGSTSGQSSSQRRHRSSSGEESVSSGQKGHSSSSSHQSSSSSQYGSGSGRSSSSKEHG
SSSGKHSGSSGAKGGSSSSHQSSSSGQYGSGSKSSGSGSCSGSGSGSGSGSGSSKSSSSGQYGSGSKSSGSGSCSGSGSGSGS
GSGSGSGSGSGSGSGSSKSSMSGQYGSGSKSSGSGSCSGSGLGSGSGSGSGSGSGSGSGSSKSSISGQYGSGSKSSGSG
SGSSSGSGSGSGSGSSKSSISGQYGSSSKSSGSGSCSGSGSGSGSGSGSGSGSNKSSSSGQYGSGSGHSSSSKGHGSSSGGKS
SSSGGRGHSSSSSTSQRHHSGSASGSGSGSCSCSGSESASESGSGHSSGSKQQGSRSGHSSRSGRHGSSSSQSSQRGHRSSSG
EESVSSGQKGHSSSSSHQSSSSSQYGSGSGHSSGSKGHGSSSGKQSGSSGAKGGSSSSHQSSSSGQYGSGSGSGSCSGSGSGS
SSSKSPSSGQCGSGSDQSSSSKGHRSSSKAKIGIPAREGDSSSSHQSSSSGQHSSGTGSDYGYGYGSSQSSISGQCGSGSDQS
SSSKGHGSSSGKESESCGSKGERFESY

>Md_RPTN (XP_001372240.1)
MAPLLNSILTVIEVFHEYAKENDDCTSLGKKELKQLLLKEFQEILRRPHDPQTVDTILQLLDRDHDEHIDFNEFLLLVFKLAQ
ACYLTQSKKLCRDKTGSHQERGREKTRDHHLPEEGMGKQQKQKHRGQRQDSHRGQSERQDRDSRYGQSERQDRDSRYGQSERQ
DRDSRYGQSERQDRDSHYGQYEGQDEDSHYGQHEGHDHHCHHDHSGKDQHSHHGQDERQDHHSHHDQSERQDNTSRCGQSKRL
GQDSHHTNSSRPGQDIHSDQSKRRGQEAGCGQSERQSYVSCLGLNKRQSFCGSKPEGQDHGSHYGQSQRLGQESNCGQTDRQR
LNTGNSHSGRQGQEYGCGQTDRQGLDSHCGQSGRQGLDSHYGQSRGQELGTQCGQSGRQGLDSHYGQSGSQGLDSHYGQSGDF
GQESSYGQSGREGLDSHYGQSGRQGLDSHYGQSGRQGLDSHYGQSGRQGLDSHYGQSGRQGLDSHYGQSGRQGLDSHYGQSGR
QGLDSHYGQSGRQGLDSHYGQSGRQGLDSHYGQSGRQGLDSHYGQSGRQGLDSHYGQSGRQGLDSHYGQSGRQGLDSHYGQYG

GFGQDSSYGQTHRQGLDTGYGYSEVQGQNSQFQNSGRRGQAEQDLSRNQDHVSGEDRQGHFHQGLWQPERESESRQHKVYSQS
QQERTLCHKGREWQKDGTEQTGGQAQDRRGCEEKKSYQRYDQQTHKEEQSHQTRDRQGHEEQGTSSQRQNRQSCGNEQSHWTQ
DNQTQNRQTRDNEKYHQTRDRQDHQDCSNEQSHQRQDRQTRGEEESSCQTQSREAHEGQRQQKSQNQRYQGNQQNHPQTRGSS
THESHHVRHQIQGSQRRSGDQSSYPTQNHGNQRGGASNGRHTTNSAVASNPLYDYVQEQKAQGHHN

>Md_TCHH
MPQLLKSIVNIVEVYNQYAKSDCDGTALSKKALKNLLQREFGDILRRPHDPQTVDLVLQLLDRDCNGVVDFNEFLLLLFKVAQ
ACYYALSQATGNEGKRRVKLEEKRERSQDFLREENQRRRDTRERQLQEEEELQQAEQQERERQRSDARSLFERQRQERVRQSA
SEEEQLQRQEQERRLQERERQSLEDEQLQRPRKERQKERQIREEEQRQRQELEQERRRREQRQESAEGYKRQEQERQRREQQR
ERQEESLQERADQSLEEEQQLRRQEERKVREKGESRSLRLQWQLEREVEGRQNKVYSKPRRQERQFLEEESSLRQQREEERRR
QQERERQLLEEEALQRQDRFGRLRDEDFEDSFQEERARQLREEEQIRRRQIRDRQLQEEERRRDLKIQWLPEEETERRRNRLY
AKPSEQQQERERRRVQRERQLREEEERELQQLERRRPQEQLYREEERLEQEEEQLRRRQEREEKRQRQERERQLREEEALQQQ
DRFRRLRDGDLDDSFQEERARQLLEEEQIPRQQIRGRQLQEEELGRDLKIQWLPEEETERRRNRLYAKPSEQQQERKRREQLE
RQRKEEEELQQEEEQLQRLEQELEKKREELEKRRRQEQRERQYREEEQLQEEEQLQRLEQELENKLEELEKRRRQEQREKTLP
W

>Md_TCHHL1 (XP_007485516.1)
MPQLLQSVICVMEAFQKYAKEEGDCWTLNSGQLKRLLLGEIGEFLKPFDILTAGTSLHFLDRDGDGSISFDEFILLIFDLLNI
CYQDIHSFLNQEPKPKSNTEEKFSGDTEACEASEDYQKVAGPDQYEQRLTGTESPSLVNPIEKVPDTVSKEDPQDDLESPKLL
GKEVEHNHPKSQYYGEEAKQSQERSQYVQATGADGIPPEEKKPPKEFVKRTCSQKDERIVSEEHEVPGEQVDKDPRDHPSEQE
KEQNVEIQSVHMEETAQRTSESPEGTIIKDVGEHTDTQGLASQEDHERKLGTTDLPAEKAEEKLSETERLPEPRDDDGTSDIQ
EPLQIPSEKQEQGKEYETTKIPAKGNGSRVPETEVFIDEKKEKRKPETLDTVGKKDGEKTKQMETLTEQEAEGKEKQLEGLEE
KGEIRKDSGIPDFKSVDDKNHYEHKGPAAPEEIKVSEISESEYQGDKTPETRDRVQVSGSQDDQSKGKNRRIIQILDQPIEQY
DRHQVENQDDGTLSETYSNPEDGKSGSETRDLPAQKKSQGQVDAQEQPDQGDCKNYLGTQESLVPGDKSRTPEKEVLIDKDNE
NITEEQEKLAKEKKSDTAEESQSQPESQESMKQKATETSPKIEIKNPTDDIDEQLSIVPSAEKEDHRKDPEAHGPEAEEGKGE
ESESQEAPLEGQGPETQGLALDARPDTLNPIPREDKSAQKLAGEGSELQYAVNKEDGSSPGLSELIKKKARDTKLCSSSGDEI
QSNPIYENIQKTPEQPDLLDTEEHLDQTDTSQTSSPELSVEREIQKSECSDFPALLDPLYDDTQELRNQTGDPESDEEYGNQQ
EILAYQSQEDIHGQPQQEDQQQLRDDSSRKY

>Md_TCHHL2 (XP_007485517.1)
MPQLLKGIITVIDVFYKNAWTDGGCQRLSKQELKQLLQQEFGEALQKPGHSETTDKILQLLDKDGDGTVDFSEFVFLVFSVVK
ACYACIQPLLCPGLLEGGRRYETQEPQKGRTEDIPLGSNELPATTDNHRDDENNQPQTPQNAAQCRVTAGQKESSEETRIHIR
DENTATRTSEGHHIGGETSTFRSNERKDPVNEDGGKYNVRDRFSRDDERDEVKEQTPEQRESGKRHFIETIPMPSDNRRPNVI
EEVPTQMNGRMYHDREQFPAQGDDERLQLRDEIPSPRDEERYTVREKSKEVRYNRRYQMSGQIPGALEKRRNQIKVQISSAER

C

>Md_PGLYRP3
MKMFVGFLFFSILCLGCCDGVPDVLSPDGNETESLPFNFQDLINNITLLIEKVKTPYGGSFQMVSRSEWGKGPSGCNIQLRTP
VPYLIIHHILGQECHEKATCRQRVKGLQELHIKINGWCDVAYNFLIGEDGNVYEGLGWTLEGTHTMGYNRKSLGFAFVGSAAG
SSPSAAALTAAENLISFAVYNGYLSPKYIQPLFVQSESCLACYQKGMSKKECPDIVPRSSWGAQDTDCSKLPGPAKYVVIIHT
GGRNCNETEECQIALRYIQSYHIEKMKFCDIAYNFLVGEDGKAYEGVGWDTEGAHTYGYNDIGLGIAFMGLFTDNPPNDAALK
AAQDLIQCSVDKGYLDPDYLLVGHSDVVNTLSPAQALYDQIKTCPHFKH

>Md_S100A9 (XP_001372115.3)
MENCTMEKALDIIVNTFHHYSTRVGNPDTLVKGEMKQLITKELPNFIKNAKDLQDVKHLMQELDTNQNGQVDFKEFSMMMARL
TMATHEKMHENAPDKDHHSHGPGLEGKGGSSCGSGHGHGHSH

>Md_S100A11 (XP_001366952.1)
MAKKVYPTEIERCIESLIAVFQRYAGQEKTNSSTTLSKTEFLRFMNTELASFTQNQNDPGVLDRMMKKLDLNCDGQLDFQEFL
NLIGGLAQACHKSFTDSQNIKNK

Supplementary Figure S3. Amino acid sequences of proteins encoded by EDC genes of opossum. (A) Amino acid sequences of proteins encoded by SEDC genes of opossum. **(B)** Amino acid sequences of opossum SFTPs. **(C)** Amino acid sequences of proteins encoded by other EDC genes of the opossum. To show the peculiar amino acid compositions of SEDCs and SFTPs and the importance for protein cross-linking the following amino acid residues are highlighted: lysine (K) and glutamine (Q) as potential sites of transglutamination; cysteine residues (C) as potential sites of disulfide bonds; glycine (G), proline (P) and serine (S) are highly abundant residues not directly involved in cross-linking. When available, the GenBank accession number is shown. Only the S100A proteins whose genes are flanking *PGLYRP3* and *TCHH* are included here. SEDC, single coding exon epidermal differentiation complex; SFTP, S100 fused-type protein; Md, *Monodelphis domestica*.

```
             1                                                                                     87
Hs_CRNN      M-PQLLQNINGIIEAFRRYARTEGNCTALTRGELKRLLEQEFADVIVKPHDPATVDEVLRLLDEDHTGTVEFKEFLVLVFKVAQACF
Bt_CRNN      M-PQLLRNINGIIEAFRRYARMEGDCAVLERGELKRLLEKEFADVIVKPHDPATVDEVLRLLDEDDTGTVEFKEFLVLVFKVAQACF
Em_CRNN      M-PQLLRNIHGIIEAFGRYAKTEGNCMVLTRGELKRLLEHEFADVIVKPHDPVTVDEVLRLLDEDDTGTVEFKEFLVLVFKVAQACF
Md_CRNN      M-PQLLGNIDGIIQAFSRYAKTEGDCTTLTKGELKKLLEQELADVIVKPHDPATVDQVFHLLDEDSKGTVDFKEFLVLVFKVAQACY
Oa_CRNN      M-PQLLGNIVGIIQAFNSYARTEGDCTTLSRGELKKLIEREFAEVIVKPYDPETVDTVLHLLDDDADGKVGFTEFLALVFRVAQACY
Hs_FLG2      M-TDLLRSVVTVIDVFYKYTKQDGECGTLSKGELKELLEKELHPVLKNPDDPDTVDVIMHMLDRDHDRRLDFTEFLLMIFKLTMACN
Em_FLG2      M-TDLLRSVVTIIDIFYKYTRQDGECGTLSKEELKELLEKEFHPILKNPDDPDTVDVIMHMLDRDHDRRLDFTEFLLMVFKLAMACN
Bt_FLG2      M-TNLLRSVVTVIDTFYKYTKQDGECGTLSKDELKELLEKEFRPILKNPDDPDTVDVIMHILDRDHDRRLDFTEFLLMVFKLAMACN
Md_FLG2      M-SVLLQSIVNIIDIFYQYAEDDGECETLSKKEMKELLEKELSSIMSNPKDPQIIEAIFHILDQDHDDKVNFAEFLLMVFKLAMSFN
Hs_FLG       M-STLLENIFAIINLFKQYSKKDKNTDTLSKKELKELLEKEFRQILKNPDDPDMVDVFMDHLDIDHNKKIDFTEFLLMVFKLAQAYY
Bt_FLG       M-STLLENINDIIKIFHKYSKTDKETDTLSEKELKELVEVEFRPILKNPGDPDTAEVFMYNLDRDHNNKIDFTEFFLMVFKVAQVYY
Em_FLG       M-STLLENITAIIDLFQQYSSNDKENDTLSKKELEELLETEFQPILENPNDPDTADDFMHILDLDHNKKVDFTEFFLMVFKLAQAYY
Md_FLG       M-SHLLSSILSIIEVYYKYTSQDQDCNTLCKRELKKLLENEFRPILKNPDDPDTVEIFMQMLDRDHDKKVDFIEFLLMIFKLTMACN
Oa_SFTP1     M-SQLLKSIVTVIDIFYHYTQGDGDCETLSKGELKELLEKEFRPILKHPNDPDTVDVIMQILDRDHDRRVDFTEFLLMVFKLTQACN
Ta_SFTP1     M-SQLLESIATIIDVFYHYTQGDGHCETLSKRELKELLKKEFRPILKNPNDPDKVDAIMQMLDQDHNRRVNFTEFLLLVFKLTQACN
Ta_SFTP2     M-SQLLKSIATIIDVFYHYTQRDGDYETLSKRELKELLKKEFHPILKHPNDPDTVDIIMQMLDQDHDRRVNFAEFLLMVFKLTQACN
Ta_SFTP3     M-SQLLKSISTIIDVFYHYTQRDGDYETLSKRELKELLKKEFRPILKHPNDPDTVDVIMQMLDQDHDRRVNFAEFLLMVFKLTQACN
Hs_HRNR      M-PKLLQGVITVIDVFYQYATQHGEYDTLNKAELKELLENEFHQILKNPNDPDTVDIILQSLDRDHNKKVDFTEYLLMIFKLVQARN
Bt_HRNR      M-PKLLQSIVTVIDIFYQYANQAGECDMLNKAELKELLENEFGQILKNPDDPDTVDIIMQNLDHDRNKKVEFTEYLLMIFKLAQACN
Em_HRNR      M-PKLLPSIVSVIEIYYQYATEEGECNSLNRAELKELLENEFRQILKNPDDPDTVDIIMQSLDRDHNKKVDFTEYLVMIFKLAQACN
Md_HRNR      M-SQLLTSMATIMDIFYTYCGKDEECDTMNQSELKEFLEKELQFILKNPDDPETIDIMMLNMDLDHNKKIDFPEFLLLVFKLIMILN
Md_HRNRL1    M-SQLLRSIVTVIDVFYNYCGQDEECDTMCKKELKELLYKEMGPLLKNPNNPDTVDIFMQILDRDHDRRVDFTEYLLMVFKLTMACN
Md_HRNRL2    M-SQLLTRIATIIDVFNQYCGQDKECDMISQQELKEFLENELQFIVQNSEDSEAVDVIMLNMDLDQSGRGHLTDLVFQIIKKILLMK
Md_HRNRL3    M-SQLLTSITTILDVFYQYCGEDEECDTMSQSELKKFLENELRFILKNPEDPDTVDVIMLNMDQDRDKRVDFPEFLLLIFKITMSFN
Md_TCHHL1    M-PQLLQSVICVMEAFQKYAKEEGDCWTLNSGQLKRLLLGEIGEFLKPF-DILTAGTSLHFLDRDGDGSISFDEFILLIFDLLNICY
Hs_RPTN      M-AQLLNSILSVIDVFHKYAKGNGDCALLCKEELKQLLLAEFGDILQRPNDPETVETILNLLDQDRDGHIDFHEYLLLVFQLVQACY
Bt_RPTN      M-TELLNSILTVIRVFQKYAKENGDSTSLCKEELKQLLLAEFGDILRRPNDPETVETILSLLDRNRNEHVDFHEYLLMVFQLAQACY
Em_RPTN      M-AQLLSSILTVIKVFQKHASENGDCTSLCKKELKQLLLAEFGDILWRPNDPETVETILTILDRDSNGHIDFHEYLLLVFQLAQACY
Ta_RPTN      M-SQLINSILRIIEVFQNYANADDNCVSLTKTELSELLQAEFGNLLRRPRDPKTVDTILQLLDRDRDGVVGFNEFLLLVFKMAQACH
Md_RPTN      M-APLLNSILTVIEVFHEYAKENDDCTSLGKKELKQLLLKEFQEILRRPHDPQTVDTILQLLDRDHDEHIDFNEFLLLVFKLAQACY
Hs_TCHH      M-SPLLRSICDITEIFNQYVSHDCDGAALTKKDLKNLLEREFGAVLRRPHDPKTVDLILELLDLDSNGRVDFNEFLLFIFKVAQACY
Bt_TCHH      M-SPLLRSIFNITKIFNQYASHDCDGTTLSKKDLKNLLEREFGDILRRPHDPETVDLVLELQDRDRDGLIDFHEFLAIVFKVAAACY
Em_TCHH      M-PALLRSICDITEIFNQYASNDCDRAALCKKDLKELLEREFGDVLRRPHDPETVDLVLELLDRDHNGLIDFHEYLLLIFRMAQACY
Md_TCHH      M-PQLLKSIVNIVEVYNQYAKSDCDGTALSKKALKNLLQREFGDILRRPHDPQTVDLVLQLLDRDCNGVVDFNEFLLLLFKVAQACY
Ta_TCHH      M-SPLLKSIIDISEIFNYYATCDGDDVKLNKGELRTLLQREFRDVLRRPQDPQTVDLVLQLLDRDRDGSVDFNEFLLLVFKVAQACY
Hs_TCHHL1    M-PQLLRNVLCVIETFHKYASEDSNGATLTGRELKQLIQGEFGDFFQPC-VLHAVEKNSNLLNIDSNGIISFDEFVLAIFNLLNLCY
Bt_TCHHL1    M-PQLLRDILCVIETFHKYAREDA--ATLTCTELKQLIQSEFEDIFQPC-AIHAVERNLNLLNIDSNGAISFDEFVLAIFSFLNVCY
Em_TCHHL1    M-PRLLRRVLCVIETFHKYAREDGDGVTLTHRELKQLLQGEFGDILQPH-VMHAVEKNVNLLDIGSDGTIRFDQFVLATCNLLNHCY
Oa_TCHHL1    M-PRLLKSVTDVMEVFHKYAQEDGGQAVLTKDGLRQLLRSELGDILQRSPDGRYVGSEVDLLDANHDGVIDFNEFILLVFGLLNACY
Bt_TCHHL2    MTHRLLRSIISIIDAFLPNAKSDGDCQSLNKTELKKLLQEEFGNALEESNNSETTGKILQQLDQDGDQTIDFSELILLMFAVTTAYY
Md_TCHHL2    M-PQLLKGIITVIDVFYKNAWTDGGCQRLSKQELKQLLQQEFGEALQKPGHSETTDKILQLLDKDGDGTVDFSEFVFLVFSVVKACY
```

Supplementary Figure S4. Amino acid sequence alignment of S100 domains of SFTPs. Amino acid sequence alignment of SFTP proteins of human, cattle, elephant, opossum, platypus and echidna. Amino acid residues conserved in all species are colored in red. Amino acid residues and highly similar residues (D/N, D/E/Q, L/M, I/V, F/Y) conserved in >50% of all investigated species are colored in blue. Species: Human (*Homo sapiens*), cattle (*Bos taurus*), elephant (*Elephas maximus indicus*), opossum (*Monodelphis domestica*), platypus (*Ornithorhynchus anatinus*) and echidna (*Tachyglossus aculeatus*). Accession numbers: human CRNN, NP_057274.1; human FLG2, NP_001014364.1; human FLG, NP_002007.1; human HRNR, NP_001009931.1; human RPTN, NP_001116437.1; human TCHH, NP_009044.2; human TCHHL1, NP_001008536.1; cattle CRNN, NP_001178232.1; cattle RPTN, XP_010801377.1; cattle TCHH, XP_002686080.3; cattle TCHHL1, NP_001193524.1; cattle TCHHL2, XP_024845739.1.

A

```
                    M  A  Q  L  L  N  S  I  L  S  V  I  D  V  F  H  K  Y  A  K  G   N  G  D  C  A  L  L  C  K  E   E  L
Human RPTN         ATGGCTCAACTCCTGAATAGCATACTCAGTGTGATTGACGTATTCCACAAATATGCCAAAGG-GAATGGGGACTGTGCCTTACTATGCAAGGAA-GAGTT
Platypus RPTN      ATGTCTCAGCTCGTCAGAAGCATCCTCAGGGTCATCGAGGTTTTCCAAAGCTATGCCAATGCCGGACGACAACTGCGTCTCGCTGGACCAAGACCGAGCT
                    M  S  Q  L  V  R  S  I  L  R  V  I  E  V  F  Q  S  Y  A  N  A  G  R  Q  L  R  L  A  G  P  R  P  S
                                                                   frameshift                      frameshift


                     K  Q  L  L  L  A  E  F  G  D  I  L
Human RPTN         GAAACAACTGCTCTTGGCTGAGTTTGGAGACATCCTCC
Platypus RPTN      CAAGGAACTTCTGCAAGCAGAGTTCGGCAACCTTCTGA
                   S  R  N  F  C  K  Q  S  S  A  T  F  -
                                                      stop
```

B

```
                    M  S  P  L  L  R  S  I   C  D  I  T  E  I  F  N  Q  Y  V  S  H  D  C  D  G
Human TCHH         ATGTCTCCACTTCTGAGAAGCATC-TGTGACATCACTGAAATTTTCAATCAGTATGTCTCTCATGATTGTGATGG
Platypus TCHH      ATGTCTCCCCTCCTGAAGAGCATCATCCGACATTTCCGAGATCTTCAACCACTACGCCACGTGCGACGGCGATGA
                    M  S  P  L  L  K  S  I  I  R  H  F  R  D  L  Q  P  L  R  H  V  R  R  R  -
                                     frameshift                                     stop
```

Supplementary Figure S5. *RPTN* and *TCHH* are pseudogenes in the platypus. (A) Nucleotide sequence alignment of human and platypus (*Ornithorhynchus anatinus*) *RPTN* until the first in-frame stop codon of the platypus *RPTN* pseudogene. Nucleotide insertions leading to frameshifts are marked by red shading. The corresponding amino acid sequences are shown above and below the nucleotide sequences. **(B)** Nucleotide sequence alignment of human and platypus *TCHH* until the first in-frame stop codon of the platypus *TCHH* pseudogene. A nucleotide insertion leading to a frameshift is marked by red shading. The corresponding amino acid sequences are shown above and below the nucleotide sequences. Note that the premature stop codons in platypus *RPTN* and *TCHH* are located in coding exon 1. Supplementary Table S1 provides information about accession numbers and the positions of the platypus *RPTN* and *TCHH* pseudogenes from the start codon to the first in-frame stop codon.

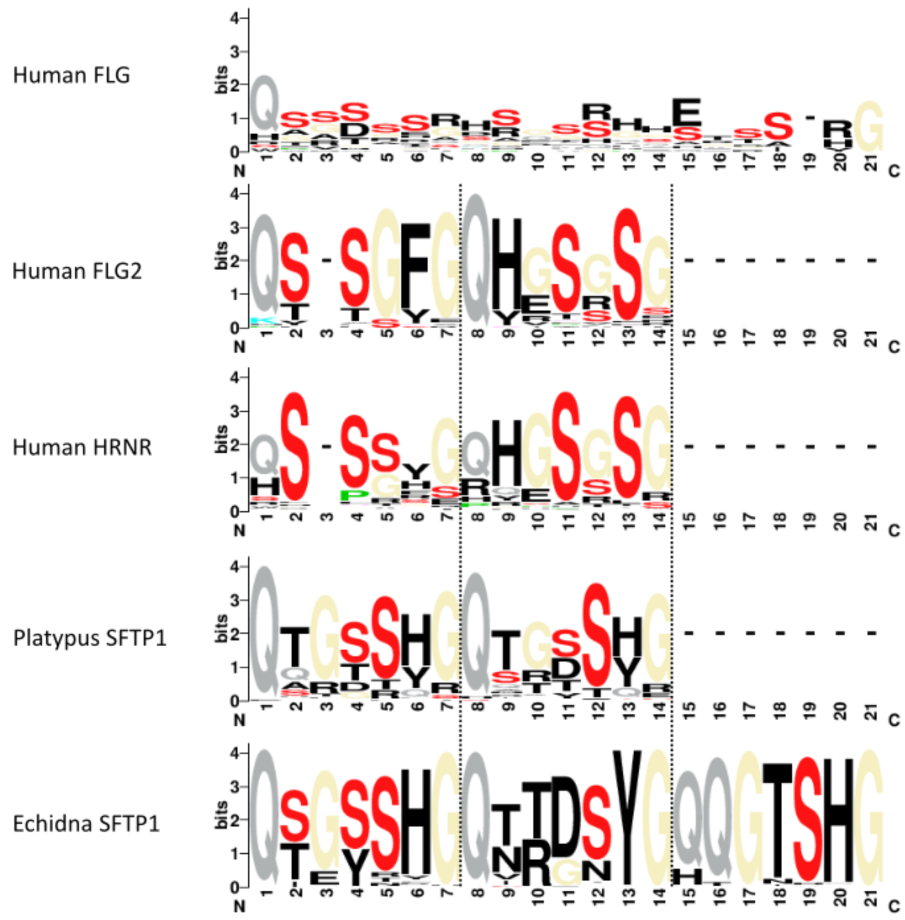

Supplementary Figure S6. The sequence repeats in the carboxy-terminal domain of platypus and echidna SFTP1 proteins are similar to those of human FLG2 and HRNR. Comparison of sequence repeat motifs of human, platypus and echidna SFTPs. Vertical dotted lines mark the borders of segments of similar sequence within longer repeats. Amino acid residues are presente in the one-letter code. Dashes mark gaps that were introduced to optimize the alignment. Note that human FLG does not contain sequence repeats of a length similar to those of the other SFTPs shown here, resulting in a sequence motif with low conservation (small height of letters) at all positions. Species: Human (*Homo sapiens*), platypus (*Ornithorhynchus anatinus*), echidna (*Tachyglossus aculeatus*). GenBank accession numbers: human *FLG*, NP_002007.1; human *FLG2*, NP_001014364.1; human *HRNR*, NP_001009931.1. SFTP, S100 fused-type protein.

A

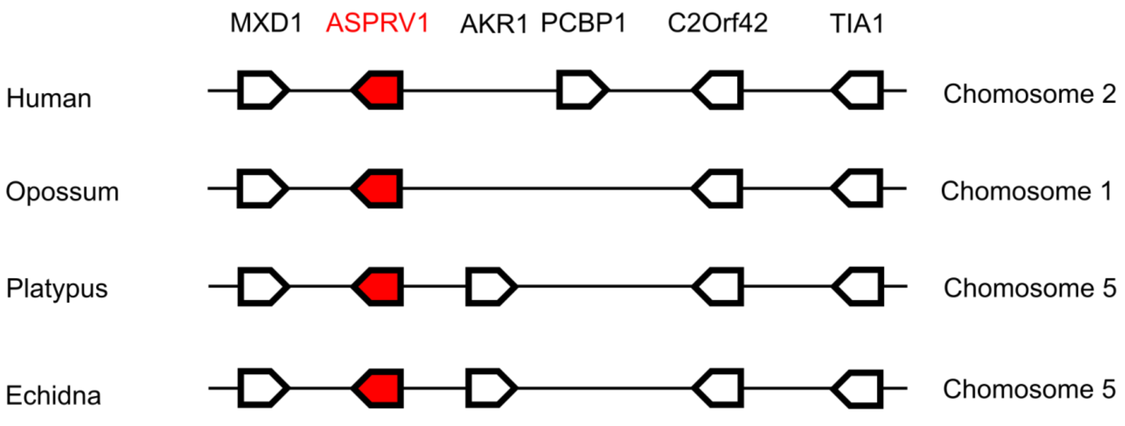

B

```
                                                                                                                 100
Human    ASPRV1  MAGSGAR---SEEGRRQHAFVPEPFDGANVVPNLWLHSFEVINDLNHWDHITKLRFLKESLRGEALGVYNRLSPQDQGDYGTVKEALLKAFGVPGA---A
Opossum  ASPRV1  MAGREVR---SQSGRT-HAFTPEHFDGHNLDVYRWLHQFNVISDLNNWDDSTQMKFLTQSLKGDALDVYTGLSPEAKGNCQSVKDALFEAFRSESDFEHK
Platypus ASPRV1  MAEAGDREAGSRGPAREHAFLPEPFDGSRGDAGRWLHQLEVIADLNHWDEATKLRFLSSALRGGPLDLFRGLGPEARRSYPAVRDALLKAFG-------G
Echidna  ASPRV1  MAEAG-----SRELPREHAFLPEPFDGSRGDAGRWLHQLEVVADLNNWDEATKLRFLGSALRGGPLDLYGGLGPEARHNYPAVRAALLGAFG-------G

                                                                                                                 200
Human    ASPRV1  PSHLPKEIVFANSMGKGYYLKGKIGKVPVRFLVDSGAQVSVVHPNLWEEVTDGDLDTLQPFENVVKVANGAEMKILGVWDTAVSLGKLKLKAQFLVANAS
Opossum  ASPRV1  QSHGPREIVFANSMGKGYYLKGKIEGIPVRFLVDSGAQVSVVSPDLWEQATDGDVDTLRPFENVVKVANGAEMKILGIWDTKVTLGKFEMEAEFLVANAS
Platypus ASPRV1  PVPGPREILLARSMGKGYYLKGSIEGVPVRFLVDSGAQVSVAHPDLWEQATDGDPSTLRPFENVVKVANGAELKILGIWDTVITLGKLEMNAQFLVADEA
Echidna  ASPRV1  P--GPREILLALSMGKGYYLKGSIEGVPVRFLVDSGAQVSVAHPDLWERATDGDPDTLRPFENVVRVANGAELKILGVWDTVVTLGKLEMDAQFLVAGEA

                                                                  265
Human    ASPRV1  AEEAIIGTDVLQDHNAILDFEHRTCTLKGKKFRLLPVGGSLEDEFDLELIEEDPSSEEGRQELSH
Opossum  ASPRV1  TEEAIIGTDVLQDHGAILDFKHRTCTLRGKKFRLLPVGGSIEDEFDLELIEEETSDH--------
Platypus ASPRV1  TEEAIIGTDVLQDHGAILDFKHRTCTLKGKKFRLLPVGGSLEEEFDLELIEERPCSP--------
Echidna  ASPRV1  AEEAIIGTDLLQDHGAILDFKHHTCTLKGKKFRLLPVGGSLEEEFDLELIEERP-----------
```

Supplementary Figure S7. ASPRV1 is conserved in monotremes and therian mammals. (**A**) Gene locus of *ASPRV1* in human, opossum, platypus and echidna. (**B**) Amino acid sequence alignment of ASPRV1 proteins of human, opossum, platypus and echidna. Species: Human (*Homo sapiens*), opossum (*Monodelphis domestica*), platypus (*Ornithorhynchus anatinus*), echidna (*Tachyglossus aculeatus*). Accession numbers: Human ASPRV1, NP_690005.3; opossum ASPRV1, XP_007476366.1; platypus ASPRV1, XP_001519377.3; echidna ASPRV1, XP_038603102.1.

```
                  Q  F  Q  E  E  L  E  K  F  Q  Q  A  I  D  S  R  E  D  P  V  S  C  A  F  V  V  L  M  A  H  G  R  E  G  F  L  K  G
  Human CASP14    CTCCAGCAATTCCAGGAAGAGCTGGAAAAATTCCAGCAGGCCATCGATTCCCGGGAAGATCCCGTCAGTTGTGCCTTCGTGGTACTCATGGCTCACGGGAGGGAAGGCTTCCTCAAGGGA
Echidna CASP14L1  TTTTAGGGCTTTCGGGACGAAGTGGTGCATTTCAGAGAGGAGATGGAAAAGCGGACAGACCCCATAAGCTGCTGCTTCGTGGTGCTCATGGCTCACGGGAAGGAGGGACGCCTGCTAGGG
Echidna CASP14L5  TTTTAGGGCTTTTGGGATGAACTGGTGCGTTTCAGAGAGGAGATGGAAAAGCGGGCAGACCCCATAAACTGCTGCTTCGTGGTGCTCATGGCTCACGGGAAGGAGGGAGTTCTGCTAGGG
Echidna CASP14L6  ------------------------------------------------------------------------------------------------------------------------

               E  D  G  E  M  V  K  L  E  N  L  F  E  A  L  N  N  K  N  C  Q  A  L  R  A  K  P  K  V  Y  I  I  Q  A  C  R  G
  Human CASP14    GAAGATGGGGAGATGGTCAAGCTGGAGAATCTCTTCGAGGCCCTGAACAACAAGAACTGCCAGGCCCTGCGAGCTAAGCCCAAGGTGTACATCATACAGGCCTGTCGAGGAGGTGGGG
Echidna CASP14L1  GCAGATGGGCAGGTGGTGGAACTGGAGGAGCTTTATGATGTCCTGACTAACAAGACCTGCCGGGCACTCCTGGGCAAATCCAAAGTGTTCATCCTGCAGGCCTGCCGTGGGGGTCAGT
Echidna CASP14L5  GCAGATGGGCAGGTGGTGGAACTGGAGGAGCTGTATG--------------------------------------AAACCCAAAGTGTTCATCCTGCAGGCCTGCCCTGGGGGTCAGT
Echidna CASP14L6  ---------------------------------------------------------------------------------------------------------------------
```

Supplementary Figure S8. *Caspase-14* (*CASP14*) pseudogenes of the echidna contain partial or complete deletions of the exon encoding the catalytic site. Nucleotide sequence alignment of the third coding exon of the human (Hs, *Homo sapiens*) *CASP14* gene and the homologous sequences of echidna (Ta, *Tachyglossus aculeatus*) *caspase-14-like 1* (*CASP14L1*), *CASP14L5*, *CASP14L6* (Suppl. Table S5). Note that a deletion (indicated by dashes) of 38 nucleotides in Ta *CASP14L5* leads to a frame shift. This exon is entirely missing in the Ta *CASP14L6* gene, in which additional mutations have distroyed splice sites of other exons. The amino acid sequence encoded the human exon is shown above the nucleotide sequences. The cysteine within the motif QACRG (highlighted by yellow shading) at the end of the amino acid sequence is the active site of the caspase. Six nucleotides of the introns flanking the exonic sequences are included in the alignment. Splicing signals at the ends of introns are underlined.

A

LELP1 mRNA

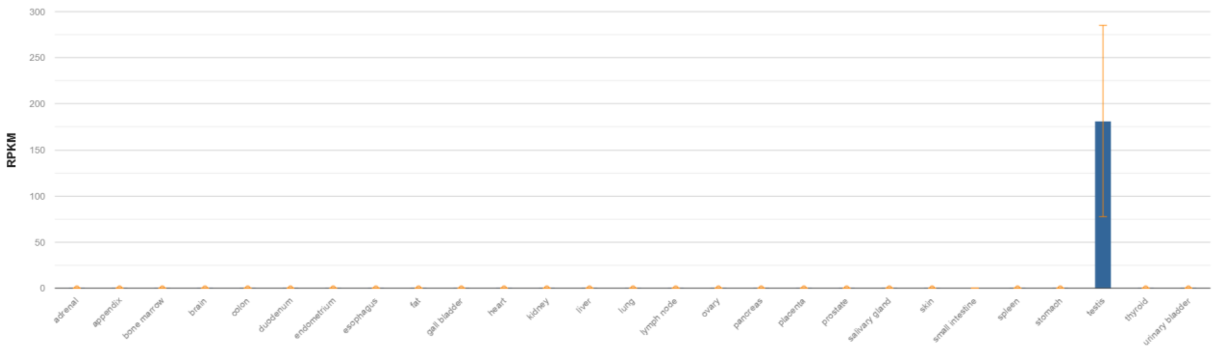

B

SMCP mRNA

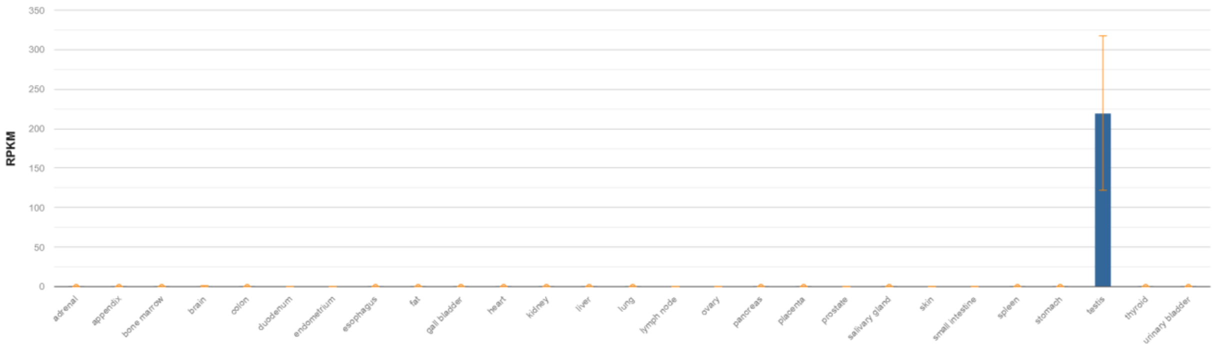

C

LELP1 protein

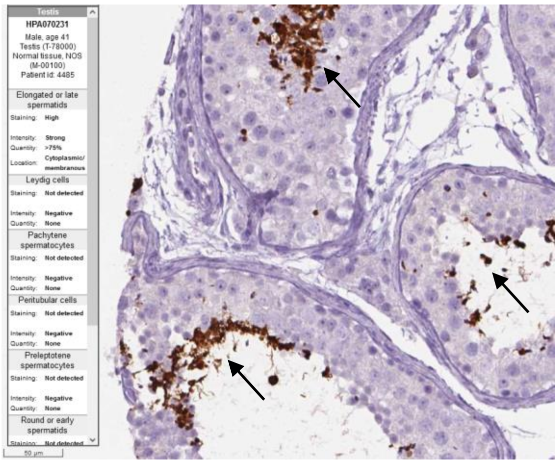

D

SMCP protein

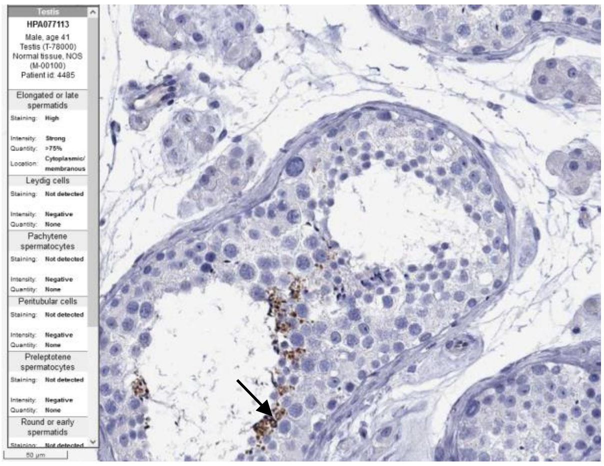

Supplementary Fig. S9 (continued on the next page)

E

CRCT1 mRNA

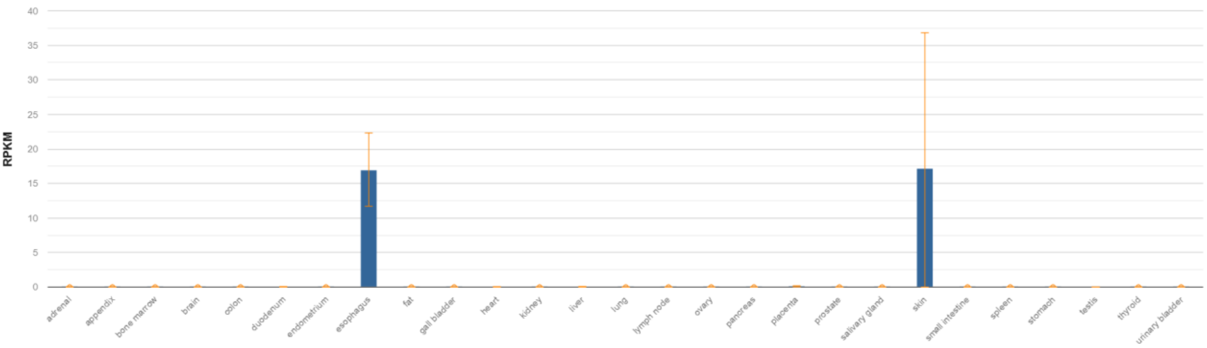

Supplementary Figure S9. Expression patterns of *LELP1*, *SMCP* and *CRCT1* in human tissues. mRNA (**A**, **B**, **E**) data were downloaded from GenBank at https://www.ncbi.nlm.nih.gov/gene/149018/?report=expression (last accessed on 4 August 2023) (**A**), https://www.ncbi.nlm.nih.gov/gene/4184/?report=expression (last accessed on 4 August 2023) (**B**) and https://www.ncbi.nlm.nih.gov/gene/54544/?report=expression (last accessed on 4 August 2023) (**E**). Protein immunolocalization data were downloaded from Protein Atlas (version 23.0, release date: 2023.06.19) at https://www.proteinatlas.org/ENSG00000203784-LELP1/tissue/testis#img (last accessed on 4 August 2023) (**C**) and https://www.proteinatlas.org/ENSG00000163206-SMCP/tissue/testis#img (last accessed on 4 August 2023) (**D**) (Uhlén M et al. 2015). Arrows point to sites of immunostaining (brown). mRNA data are publicly available without restrictions at the NCBI website. They were obtained from BioProject: PRJEB4337 (Fagerberg et al. 2014). Protein immunolocalization data were not available for CRCT1. RPKM, reads per kilobase of transcript per million mapped reads.

References:

Fagerberg L, et al. Analysis of the human tissue-specific expression by genome-wide integration of transcriptomics and antibody-based proteomics. *Mol. Cell Proteomics* **13**, 397-406 (2014).

Uhlén M, et al. Proteomics. Tissue-based map of the human proteome. *Science* **347**, 1260419 (2015).
